# Identification of a New Interesting BAG3 Modulator Able to Disrupt Cancer‐Related Pathways

**DOI:** 10.1002/cmdc.202500310

**Published:** 2025-08-11

**Authors:** Dafne Ruggiero, Eleonora Boccia, Emis Ingenito, Vincenzo Vestuto, Gilda D’Urso, Alessandra Capuano, Agostino Casapullo, Stefania Terracciano, Giuseppe Bifulco, Gianluigi Lauro, Ines Bruno

**Affiliations:** ^1^ Department of Pharmacy University of Salerno Via Giovanni Paolo II 132 84084 Fisciano Italy; ^2^ Faculty of Medicine and Dentistry and Czech Advanced Technology and Research Institute Institute of Molecular and Translational Medicine Palackу University in Olomouc Krížkovského 511/8 779 00 Olomouc Czech Republic

**Keywords:** 1,3‐dipolar Huisgen cycloaddition, BAG domain modulator, BAG3 protein, surface plasmon resonance assay, triazole scaffold

## Abstract

Continuing the research aimed at discovering new BAG3 modulators as attractive anticancer drug candidates, a screening campaign on an in‐house library is performed, including compounds featuring a large variety of scaffolds. The obtained results prompted a focus on the triazole moiety and, following a stepwise structural refinement of this scaffold guided by biophysical assays and computational studies, a very attractive compound (**2**) is identified showing a tight interaction with BAG3 and displaying a significant cytotoxic activity. The discovery of compound **2**, whose effects on apoptosis and proteostasis confirm the disruption of BAG3‐related pathways, is particularly relevant given the limited number of BAG3 modulators reported so far. Moreover, the computational data highlight the potential to further explore the triazole scaffold for the development of more potent anticancer agents.

## Graphical abstract

Compound **2**, identified through in‐house screening and derived from **SP18**, exhibits high affinity for BAG3, as demonstrated by Surface Plasmon Resonance and docking studies. It induces a dose‐dependent proapoptotic response in HeLa cells, evidenced by increased Annexin V/PI staining, underscoring the triazole scaffold as a promising platform for BAG3‐targeted anticancer drug development.

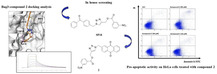

## 1 Introduction

BAG3 is an antiapoptotic protein belonging to Bcl‐2‐associated athanogene protein family, which recently gained great attention by the scientific community in virtue of its multitasking profile, being involved in key physiological processes as well as several pathologies, including cancer and neurodegeneration.^[^ ^1^ ^,^ ^2^ ^]^ BAG3 shares a conserved BAG domain with the other family members, which is located at C‐terminus, and it interacts with the ATPase pocket of HSP70, facilitating the ADP release and the nucleotide cycling. By cooperating with HSP70, BAG3 modulates the activities of this chaperone, including the delivery of client proteins to proteasome.^[^ ^3^ ^,^ ^4^ ^]^ In stressful conditions, like those occurring in cancer cells, this complex acts as a proteotoxicity sensor, detecting the increasing of misfolded proteins and assuring their clearance through autophagy and aggresome formation, thus sustaining cell protection and survival.^[^ ^5^, ^6^, ^7^ ^–^ ^8^ ^]^ A growing amount of evidence outlines the ability of the BAG3–HSP70 module to affect several pathways regulating angiogenesis and invasion, suggesting its critical role in cancer genesis and progression. Besides its cochaperone activity, BAG3 can also carry out HSP70‐independent functions through the interaction with multiple partners; indeed, owing to its multidomain architecture, it shows an extensive interactome, which leads it to play a crucial role in cancer‐promoting signaling pathways.^[^ ^9^, ^10^ ^–^ ^11^ ^]^ All this evidence, together with its overexpression in several cancer types in which it correlates with tumor grade and resistance to chemotherapy, outlines the key role of BAG3 as a promising target for cancer treatment. However, despite the great potentiality of therapeutic interventions based on the modulation of this protein, the discovery of new BAG3 binders still represents a challenging task owing to the lack of human BAG3 crystal structure, which hampers a traditional structure‐based drug design.^[^ ^12^, ^13^ ^–^ ^14^ ^]^ Continuing our research in this field and with the aim of discovering new molecular platforms able to bind and to functionally modulate the activity of this cochaperone, we undertook a screening campaign of several compounds belonging to our in‐house library to identify BAG3 ligands suitable to develop new attractive drug candidates. A Surface Plasmon Resonance (SPR) assay‐based investigation on the different scaffold types was performed, and the results highlighted that the triazole‐containing molecules showed the best binding profile for BAG3. Hence, we decided to focus on this scaffold, and following a step‐by‐step structural modification based on biophysical assays and computational studies, we identified an interesting modulator of the target of interest.

## 2 Results and Discussion

### 2.1 Screening Campaign

**Figure 1:**
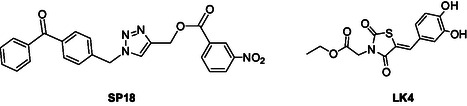
Structure of **SP18** and known inhibitor **LK4**.

To discover novel molecular platforms able to bind BAG3 and modulate its function, we undertook an extensive screening campaign using an in‐house chemical library, which included both commercially available compounds and those synthesized by our research team (see Figure S1, Supporting Information). We evaluated the binding of 60 compounds against BAG3 through SPR, ensuring a wide range of structural frameworks. Among the tested compounds, one molecule, **SP18**, which features a triazole core (**Figure** **1**), emerged as particularly noteworthy.^[^ ^13^ ^,^ ^14^ ^]^ This compound demonstrated a strong binding affinity for both the full‐length BAG3 protein and its BAG domain (**Table** **1**). Its dissociation constants indicated a binding affinity comparable to that of the well‐known inhibitor **LK4**, especially against the full‐length protein (Figure 1). This finding indicates that **SP18** could be a promising candidate for further development as a BAG3 modulator. The triazole core of **SP18** likely contributes to its effective binding, highlighting it as a unique and valuable molecule in our search for new therapeutic agents.^[^ ^15^ ^]^

**Table 1:** SPR assays of compounds **SP18** and **LK4** on BAG3 full length and BAG3‐BD. K_D_ = dissociation constant; SD = standard deviation.

Compound	BAG3 FULL K_D_ [µM] ± SD	BAG3–BD K_D_ [µM] ± SD
**SP18**	36.00 ± 0.85	43.50 ± 5.09
**LK4**	19.90 ± 6.58	0.18 ± 0.02

### 2.2 Targeted‐Limited Proteolysis Mass Spectrometry Experiment (t‐LiP‐MS)

**Figure 2:**
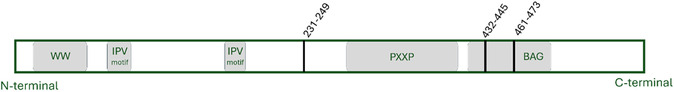
Schematic illustration of the BAG3 protein sequence. Gray boxes highlight specific domains: WW (with two conserved tryptophan residues), IPV (a tripeptide motif isoleucine‐proline‐valine), PxxP (a proline‐rich motif). Black bars indicate LiP (limited proteolysis) peptides identified as involved in binding interactions, located from amino acids 231 to 249, 432 to 445, and 461 to 473, respectively.

t‐LiP‐MS (targeted‐limited proteolysis‐mass spectrometry) is a powerful and valid proteomic approach for the analysis of ligand‐protein complexes, providing insights into the protein binding sites of the ligand. This method leverages the principle that the binding of a ligand often induces a conformational stability in its protein partner, affecting the susceptibility to enzymatic limited proteolysis.^[^ ^16^, ^17^, ^18^ ^–^ ^19^ ^]^ In the present study, t‐LiP experiments were performed to understand the site of interaction between **SP18** and BAG3. After incubating HeLa cell lysate with and without **SP18**, limited proteolysis with subtilisin was carried out for 30 min, inducing partial digestion of the protein. The presence of the ligand, altering the accessibility of the protease cleavage sites, led to differences in the resulting peptide fragments detected through mass spectrometry after extensive digestion with trypsin. This analysis was performed using liquid chromatography coupled to mass spectrometry (LC–MS) in multiple reaction monitoring mode (MRM), a selective and sensitive mode employed to detect the specific peptide transitions in a complex sample, such as a cell lysate. Specific peptides of BAG3 were monitored. Three of these peptides exhibited an increase in abundance and showed a Fold Change (Fc) value ≥1.5, thus, they were considered involved in the binding with **SP18**. Two of these peptides, ELLALDSVDPEGR [461–473] and VQGLEQAVDNFEGK [432–445], respectively, were in the BAG domain, giving crucial information for a following **SP18** derivative development provided with better BAG3 binding parameters (**Table** **2** and **Figure** **2**).

**Table 2:** Protected BAG peptides. For each peptide Q1 m/z, ID peptide sequence, fold change (Fc) values, and the p‐value were reported.

Q1 *m*/*z*	ID peptide sequence	Fc	*p* value
707.36	E [461–473] R	1.67	0.02
771.36	T [231–249] K	1.68	0.04
767.38	V [432–445] K	1.74	0.01

### 2.3 Viability Assays and Cell Cycle Analysis of SP18

**Figure 3:**
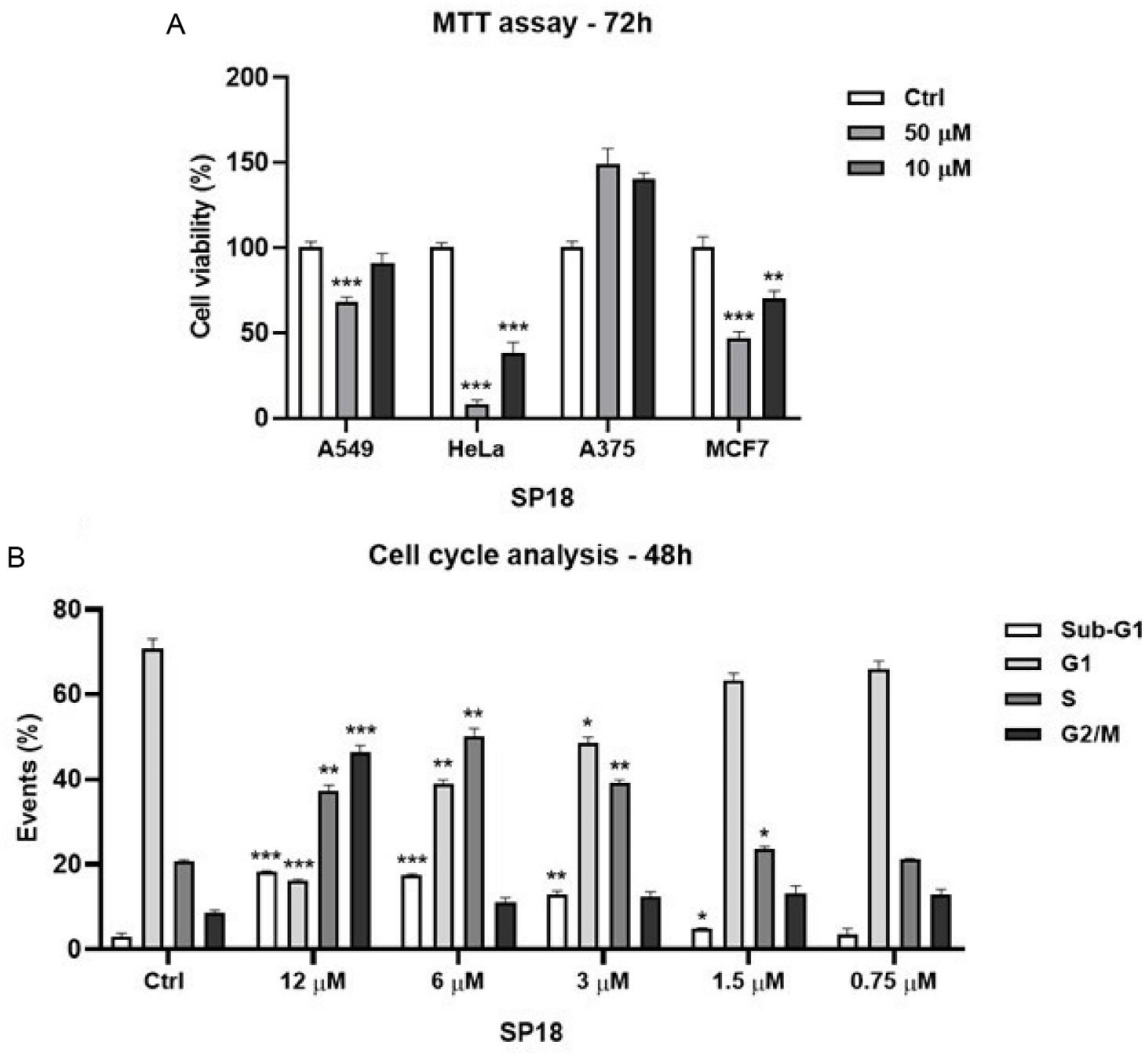
a) Antiproliferative activity of **SP18** on human lung adenocarcinoma cell line (A549), human cervical adenocarcinoma cell line (HeLa), human melanoma cell line (A375), and human breast adenocarcinoma (MCF7) after 72 h of treatment at 10 e 50 µM. b) The cell cycle analysis and the formation of hypodiploid nuclei was evaluated by flow cytometry. HeLa cells were treated with **SP18** (0.75–1.5–3–6–12 µM) and 0.1% (v/v) DMSO for 72 h. Results are shown as mean ± standard deviation (SD) from three independent experiments. *, **, *** denote respectively *p* < 0.05; *p* < 0.01 and *p* < 0.001 versus Ctrl.

After confirming the binding of **SP18** to BAG3 and determining its affinity for the BAG domain via SPR and limited proteolysis, we proceeded to evaluate its biological profile. Initially, we assessed the cytotoxic activity of **SP18** using MTT assays on four BAG3‐overexpressing cell lines: A375, HeLa, A549, and MCF7 (**Figure** **3**a).^[^ ^20^, ^21^, ^22^ ^–^ ^23^ ^]^ **SP18**, tested at 10 and 50 µM over a 72‐h period, exhibited significant cytotoxicity against HeLa cells. Consequently, we determined its IC_50_ against HeLa after 48 h of treatment, finding it to be 14.78 ± 1.34 µM. This was a promising result, especially when compared to the known inhibitor **LK4**, which was reported to have an IC_50_ greater than 50 µM in HeLa cell line.^[^ ^13^ ^]^ Next, we examined the effect of **SP18** on the cell cycle by treating HeLa cells for 48 h with five different concentrations of **SP18**, ranging from 0.75 to 12 µM. The results indicated that **SP18** induces hypodiploid nuclei formation in a concentration‐dependent manner and causes an S‐phase block in the cell cycle (Figure 3b). Encouraged by these findings, we evaluated the cytotoxic profile of **SP18** on healthy cells, as it is crucial for an anticancer drug to selectively target tumor cells without harming healthy cells. **SP18** was tested at seven different concentrations on HaCaT cells over a 48‐h period, revealing an IC_50_ of 22.10 ± 0.67 µM. Unfortunately, this result indicates that the cytotoxicity of **SP18** is comparable in both tumor and healthy cells, suggesting insufficient safety. Given these findings, we decided to halt further studies on **SP18** and focus our attention on structural modifications to improve its biological profile.

### 2.4 Step‐by‐Step Generation of a Focused Library of Derivatives through Computational Studies, Chemical Synthesis, and Binding Assessment

With the goal of optimizing the biological properties and activity of compound **SP18**, an integrated strategy combining computational studies, chemical synthesis, and biological evaluation was adopted, leading to the development of a small library of derivatives aimed at enhancing both affinity and selectivity toward the target protein.

#### 2.4.1 Step‐by‐Step Molecular Modeling Experiments and Biophysical Assays

**Figure 4:**
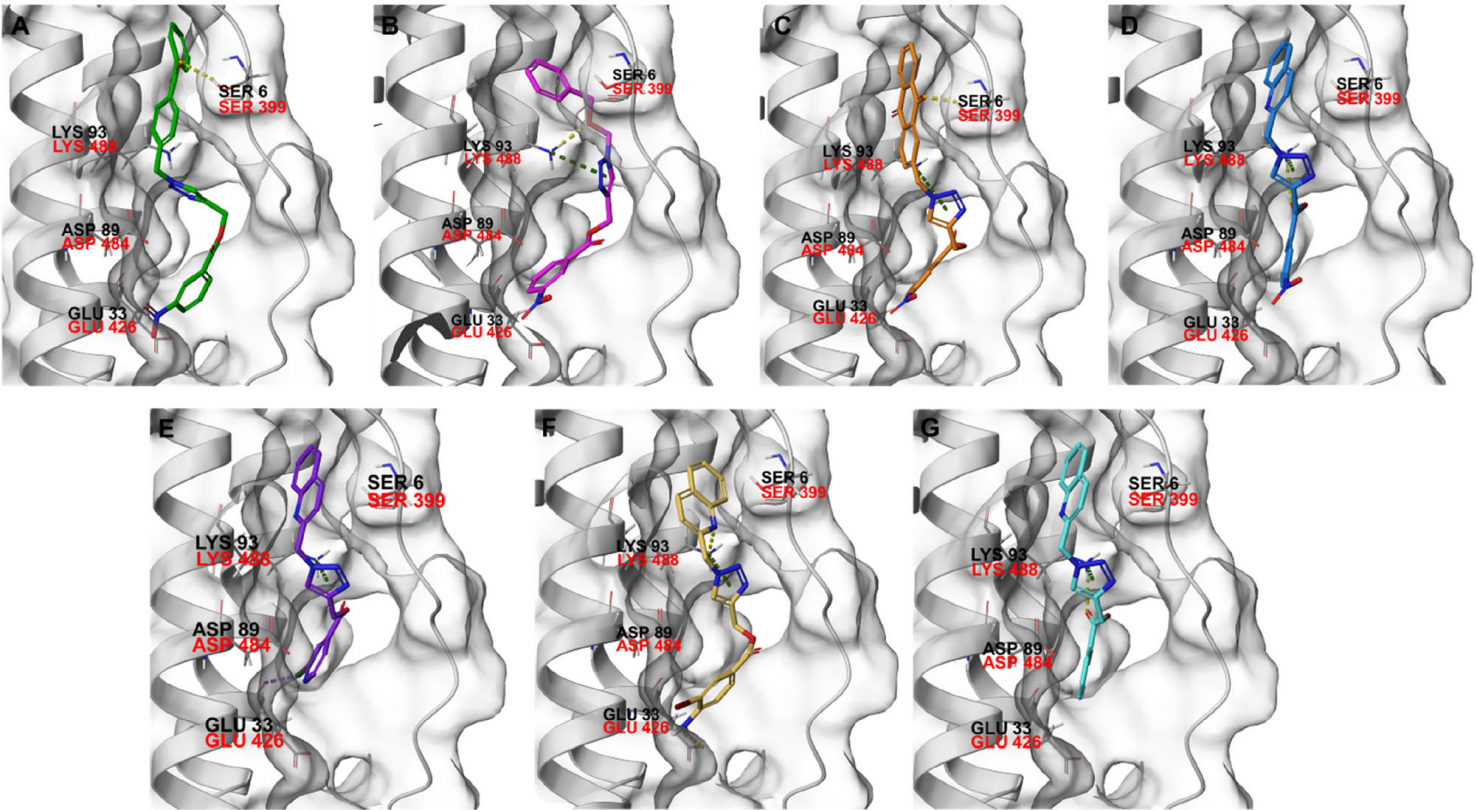
Binding mode of a) **SP18** (colored by atom type: C green, N blue, O red), b) compound **1** (colored by atom type: C faded magenta, N blue, O red), c) compound **2** (colored by atom type: C orange, N blue, O red), d) compound **5** (colored by atom type: C azure, N blue, O red), e) compound **6** (colored by atom type: C violet, N blue, O red, Cl green), f) compound **7** (colored by atom type: C yellow, N blue, O red, Br dark red), g) compound **8** (colored by atom type: C cyan, N blue, O red, I magenta). H bonds, halogen bonds, and *π* interactions are reported in yellow, violet, and green dotted lines, respectively. Residues corresponding to the sequence of murine BAG3 BD as renumbered in the Protein Data Bank (PDB code: 1UK5) and corresponding residues belonging to human BAG3 BD are reported in black and red, respectively.

**Figure 5:**
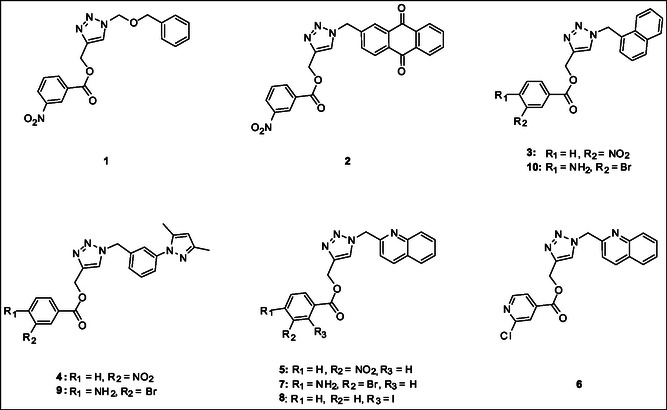
Structures of compounds **1**–**10**.

Molecular docking guided the selection of the most promising structural modifications for the synthesis and subsequent experimental evaluation of the interaction with BAG3, enabling a stepwise optimization of the compound profile leading to a set of derivatives (compounds **1–10**, **Figure** **4** and **5**).

A key step involved assessing the affinity of the synthesized derivatives for the target. To achieve this, SPR assays were conducted on both full‐length BAG3 and BAG3 BD for compounds **1–10** (Figure 5), using **LK4** as positive control. The results were highly promising: out of the ten synthesized and tested compounds, six molecules showed binding to the protein, specifically to its domain, with dissociation constants in the low micromolar range. Apart from compound **6**, all compounds demonstrated greater affinity for the full‐length protein compared to the known inhibitor. In particular, compounds **1** and **2** stood out as the most promising in terms of binding to the domain of interest (**Table** **3**).

**Table 3:** SPR assays of compounds **1**–**10** and **LK4** on BAG3 full length and BAG3‐BD. K_D_ = dissociation constant; SD = standard deviation.

Compound	Structure	BAG3 FULL K_D_ [µM] ± SD	BAG3–BD K_D_ [µM)] ± SD
**1**	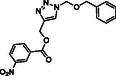	3.31 ± 0.18	1.87 ± 0.03
**2**	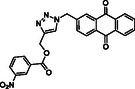	1.04 ± 0.03	0.38 ± 0.05
**3**	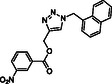	No binding	Not determined
**4**	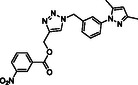	No binding	Not determined
**5**	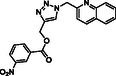	12.50 ± 2.26	8.27 ± 0.98
**6**	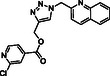	24.00 ± 4.60	2.78 ± 0.88
**7**	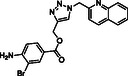	11.40 ± 2.43	6.32 ± 0.12
**8**	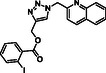	12.60 ± 1.20	2.43 ± 0.09
**9**	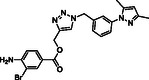	No binding	Not determined
**10**	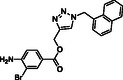	No binding	Not determined

To support the optimization process, the binding of **SP18**, used as a reference compound for developing the derivative series, was first elucidated against BAG3 domain through molecular docking calculations. Given the lack of experimental structures of the human BAG3 domain in complex with known inhibitors, the NMR solution structure of the murine BAG3 BAG domain (BAG3‐BD) (PDB: 1UK5) was employed, as it shares 93% sequence identity with the human BAG3‐BD.^[^ ^24^ ^]^ Molecular docking results revealed that **SP18** binds the BAG3‐BD by interacting with the key amino acids within the binding site (Figure 4a and S2a, Supporting Information). Specifically, **SP18** establishes an H‐bond with Ser6 (corresponding to Ser399 in the human BAG3‐BD) through the carbonyl group of the benzophenone moiety and two polar interactions with Glu33 (corresponding to Glu426 in the human BAG3 BD) and Asp89 (corresponding to Asp484 in the human BAG3‐BD) through the nitro group (Figure 4a and S2a, Supporting Information).^[^ ^25^ ^,^ ^26^ ^]^ However, its triazole core does not properly engage with the binding site of BAG3 and, also, we detected the lack of interactions with Lys93 (corresponding to Lys488 in the human BAG3‐BD) key residue, which is in accordance with the moderate affinity experimentally detected for **SP18** against BAG3‐BD. In general, the analysis of the interactions of **SP18** with BAG3‐BD from docking calculations was in line with the results of t‐LiP‐MS experiments, and, in particular, we detected the predicted binding of the ligand in a protein region close to E [461–473] R protein sequence corresponding to the human BAG3‐BD (see Paragraph 2.2). Prompted by the need for further structural refinement to obtain affine and selective BAG3 binders, we explored the possibility of replacing the benzophenone moiety while retaining the 3‐nitrobenzoate group, and based on the availability of aldehydes in our laboratory, we synthesized compounds **1**, **2**, **3**, and **4**. SPR assay highlighted that compounds **3** and **4** were not able to bind both the BAG3 full‐length and BAG3‐BD, whereas compounds **1** and **2** exhibited *K*_D_ values in the low micromolar range (Table 3). The visual inspection of the poses arising from molecular docking experiments confirmed their ability to occupy the same binding region as the reference compound **SP18**, with a conserved *π*‐cation interaction established by the triazole core with Lys93 (Figure 4b,c, S2b, S2c, Supporting Information). Beyond this shared interaction, a comprehensive analysis of the docking poses further revealed additional interactions for compound **1**, which interacted with Asp89 through the nitro group, and for compound **2**, which again interacted with Asp89 through the nitro group and, above all, with Ser6 (H‐bond through the carbonyl portion) key residue.^[^ ^25^, ^26^, ^27^ ^–^ ^28^ ^]^ Encouraged by these findings, we focused on the most promising compound **2**, evaluating the possibility of replacing the anthraquinone moiety with alternative groups to generate new derivatives with potentially improved activity and to understand if the H bond with the Ser6 is fundamental for the binding with the protein. Specifically, SPR analysis for compound **5**, in which the anthraquinone core was replaced with a quinoline moiety, revealed a weaker binding affinity (*K*_D_ BAG3 FULL = 12.50 ± 2.26 µM and *K*_D_ BAG3‐BD = 8.27 ± 0.98 µM). This result was further supported by molecular docking studies (Figure 4d and S2d, Supporting Information), which showed an H‐bond with Lys93 and a polar interaction with Asp89 but, on the contrary, the lack of the key H‐bond with Ser6. Given the outcome, we finally wondered whether the replacement of the 3‐nitrobenzoate group of **5** with other moieties could enhance the binding affinity. Accordingly, we retained the quinoline group and substituted the 3‐nitrobenzoate group with a 2‐chloropyridin‐3‐carboxylate, 4‐amino‐3‐bromobenzoate, and 2‐iodobenzoate, generating compounds **6**, **7** and **8**, respectively (Figure 4e–g and S2e–g, Supporting Information). If compared to compound **5**, only compound **7** exhibited a slight improvement in binding affinity, as disclosed by SPR analysis and in agreement with molecular docking, which highlighted two H‐bonds with Glu33 and Lys93 and, again, the lack of interactions with Ser6 (Figure 4f and **S2f**, Supporting Information). In the attempt to optimize the promising compound **7**, we replaced its quinoline moiety with a pyrazo phenyl group while maintaining the 4‐amino‐3‐bromobenzoate moiety, yielding compound **9**. However, this compound did not show the ability to bind the protein counterpart, as subsequently confirmed by molecular docking calculations, which indicated the loss of key H‐bond interactions with the fundamental amino acids. This result was further corroborated by the biological evaluation of compound **10**, synthesized as negative control, as the hydrophobic naphthalene group, replacing the quinoline, did not show a satisfactory binding, as expected in accordance to docking results.

Summarizing, attempts to modify the chemical structure of compound **2** to improve the binding affinity resulted in the loss of the crucial H‐bond with Ser6. This interaction, along with that with Lys93, appears to be essential for both target binding and biological activity (*vide infra*). The analysis of these in silico data paves the way for further deeper and systematic investigations aimed at obtaining more affine compounds.

### 2.5 Synthesis

**Scheme 1:**
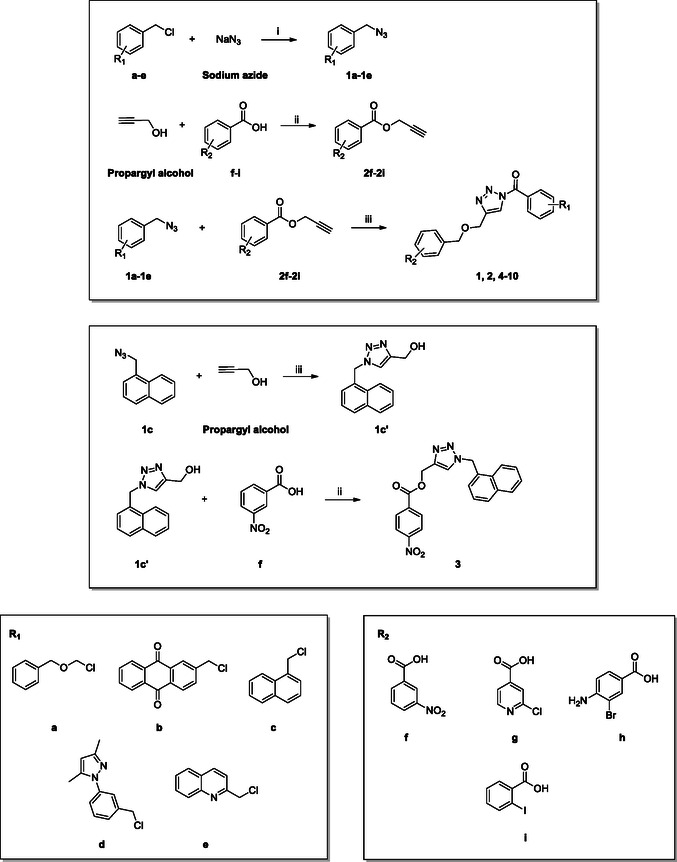
Synthesis of compounds **1**–**10**. Reagents and conditions: i) K_2_CO_3_, DMA, r.t., 4 h; ii) DIC, DMAP, DCM, r.t. 16 h; iii) CuSO_4_·5H_2_O, tBut/H_2_O (2:1), r.t.,12 h.

The synthetic strategy consisted of three main steps. First, chlorides (**a–** **e**) were converted into azides using potassium carbonate and DMA as the solvent, yielding intermediates **1a**–**1e**. Simultaneously, various carboxylic acids (**f–** **i**) were esterified with propargyl alcohol to produce intermediates **2f**–**2i**. The final step involved a 1,3‐dipolar Huisgen cycloaddition between the terminal azides and alkynes to form the final 1,2,3‐triazoles, resulting in compounds **1**, **2**, **4–10** (**Figure** **5**). This reaction was catalyzed by copper sulfate pentahydrate in a 2:1 tert‐butanol/water mixture at room temperature, left overnight.^[^ ^29^ ^,^ ^30^ ^]^ For compound **3**, a different approach was used: the azide formation was followed by the Huisgen cycloaddition, and the final step was the esterification. Reversing the reaction steps allowed us to achieve a yield of 51% for compound **3**, whereas the original sequence gave a yield of only 23% (**Scheme** **1**).

### 2.6 Viability Assays and Cell Cycle Analysis for SP18 Derivatives

**Figure 6:**
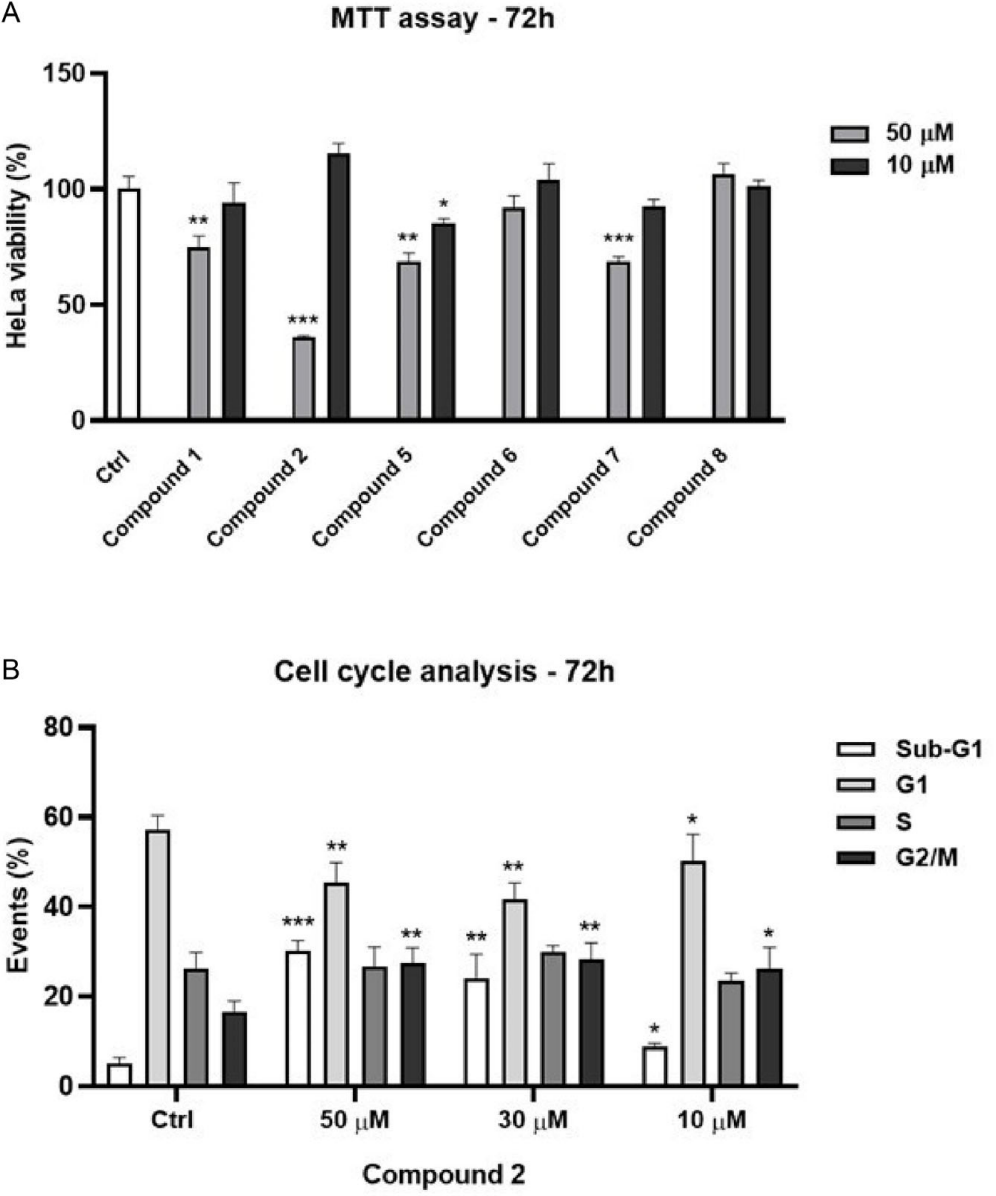
a) Antiproliferative activity of compound **1**, **2**, **5–8** on human cervical adenocarcinoma cell line (HeLa) 72 h of treatment at 10 e 50 µM. b) The cell cycle analysis and formation of hypodiploid nuclei was evaluated by flow cytometry. HeLa cells were treated with compound **2** (10, 30 and 50 µM) and 0.1% (v/v) DMSO for 72 h. Results are showed as mean ± SD from three independent experiments. *, **, *** denote respectively *p* < 0.05; *p* < 0.01 and *p* < 0.001 vs. Ctrl.

As previously mentioned, BAG3 is often up‐regulated in different cancer types, driving tumor progression, metastasis, and resistance to treatments.^[^ ^7^ ^,^ ^31^ ^]^ A promising strategy involves developing BAG3 inhibitors that selectively target cancer cells while sparing healthy ones to minimize adverse effects. Therefore, we examined the cytotoxicity of the six BAG3‐binding molecules, **1**, **2**, **5–8**, on HeLa cells. The evaluation was conducted at concentrations of 10 and 50 µM over 72 h, revealing that compound **2** induced ≈70% mortality at 50 µM (**Figure** **6**a). Subsequently, we determined the IC_50_ for compound **2** on HeLa cells after 72 h of exposure, finding it to be 40.76 ± 5.50 µM. The different incubation times used to determine the IC_50_ values for compound **2** and **SP18** reflect their distinct cytotoxicity profiles. Before proceeding with further investigations, we assessed the cytotoxicity of compound **2** on healthy HaCaT cells to ensure safety. After 72 h of treatment, no significant cytotoxicity was observed for compound **2** up to 70 µM, the highest concentration tested, indicating a favorable safety profile. Finally, we explored the impact of compound **2** on cell cycle progression in HeLa cells over 72 h at three different concentrations (10, 30, and 50 µM). Our findings revealed that compound **2** induces hypodiploid nuclei formation in a concentration‐dependent manner (cells in sub‐G0 phase), leading to cell cycle arrest, similar to the effects of the hit compound **SP18** (Figure 6b).

### 2.7 Annexin V‐FITC/PI Staining

**Figure 7:**
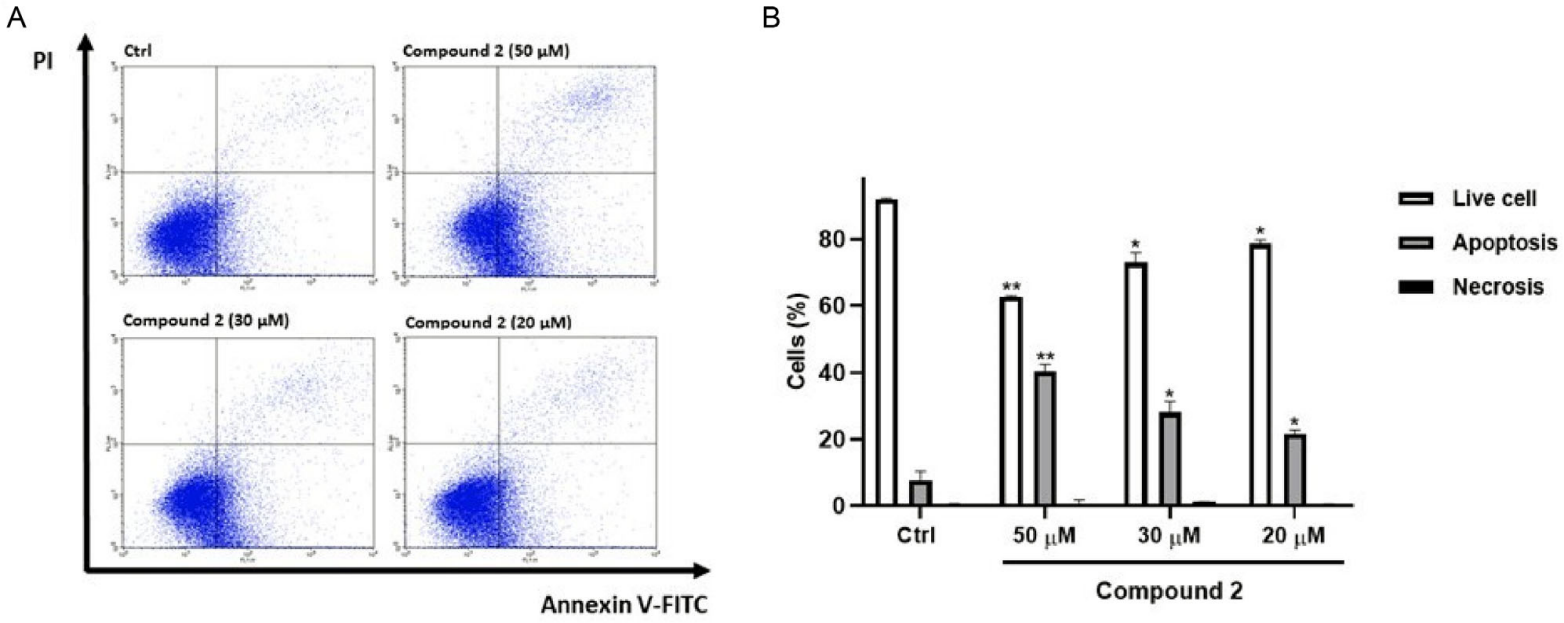
Proapoptotic activity on HeLa cells treated for 72 h with different concentrations of compound **2**. a) Representative Annexin V‐FITC/PI flow cytometry plots of HeLa cells treated with different concentrations of compound **2** (20, 30, and 50 μM). b) Related quantitative analysis is reported. Data are expressed as a percentage of live, apoptotic and necrotic cells. Results are shown as mean ± SD from three independent experiments. *, **, denote, respectively, *p* < 0.05 and *p *< 0.01 versus Ctrl.

Considering the established antiapoptotic function of BAG3, Annexin V‐FITC/PI staining was conducted to assess the impact of compound **2** on cell apoptosis. Flow cytometry analysis revealed that treatment with compound **2** induced apoptosis in HeLa cells in a dose‐dependent manner (20, 30 and 50 µM). Specifically, at a concentration of 50 µM, 40.2 ± 2.11% of cells underwent apoptosis (*p* < 0.01 vs. control), while at 30 and 20 µM, apoptosis levels were 28.0 ± 3.41% (*p* < 0.05 vs. control) and 21.6 ± 1.14% (*p* < 0.05 vs. control), respectively (**Figure** **7**).

### 2.8 Determination of Protein Misfolding

**Figure 8:**
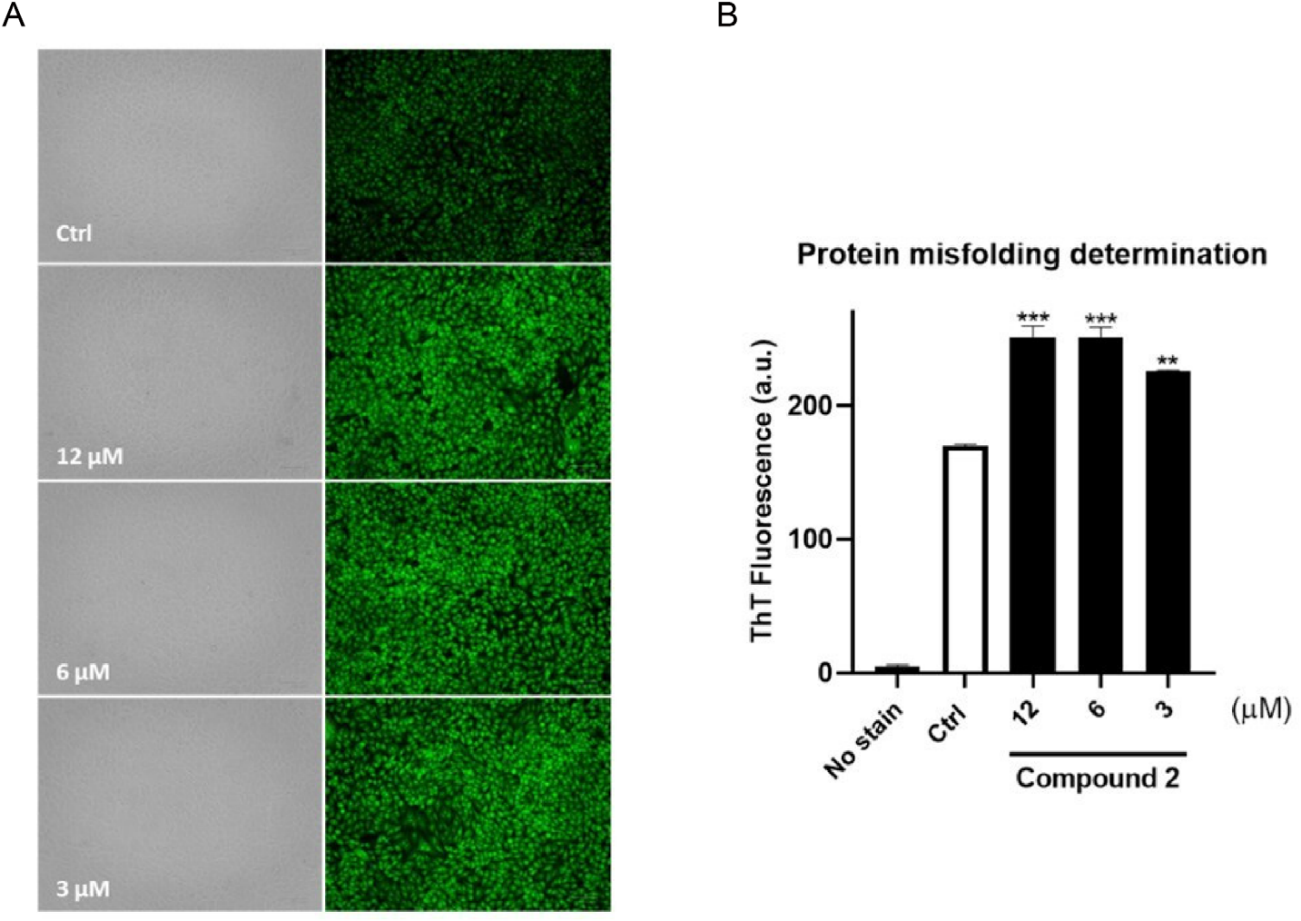
Compound **2** induced protein misfolding in HeLa cells. a) Thioflavin T (ThT) assay was performed, and live cells were observed under a fluorescence microscope. Scale bar: 100 μm. (*N* ≥ 5). Magnification 20×. b) Quantitative analysis of ThT‐positive cells was reported. Data are shown as mean ± SD of three different experiments performed in triplicate. **p < 0.01 versus Ctrl; ***p < 0.001 versus Ctrl.

The inhibition of BAG3 by compound **2** disrupts the functional partnership between BAG3 and HSP70, the critical molecular chaperone system responsible for maintaining proteostasis under both physiological and stress conditions.^[^ ^32^ ^,^ ^33^ ^]^ We hypothesized that compound **2**, by inhibiting BAG3, could induce protein misfolding in tumor cells, given the crucial role of BAG3 in maintaining proteostasis in cooperation with HSP70.^[^ ^34^ ^,^ ^35^ ^]^ Disrupting this interaction was expected to increase the accumulation of misfolded proteins and lead to proteotoxic stress.^[^ ^32^ ^,^ ^36^ ^,^ ^37^ ^]^ Using thioflavin T (ThT) staining, a specific marker for misfolded protein aggregation, we observed a significant induction of protein misfolding and aggregate formation in compound **2‐**treated cancer cells (**Figure** **8**) (6 and 12 μM; *p* < 0.001 vs. Ctrl; 3 μM; *p* < 0.01 vs. Ctrl).^[^ ^38^, ^39^ ^–^ ^40^ ^]^ These findings confirmed that compound **2** effectively impairs BAG3–HSP70 functionality, leading to proteostasis disruption in HeLa cells and highlighting its potential as a therapeutic agent against malignancies. In fact, this proteotoxic burden disrupts cellular homeostasis in tumor cells, triggering stress responses and impairing key survival pathways, activating apoptosis as previously observed (Figure 7). Consequently, the induction of misfolded protein stress by compound **2** can undermine tumor cell viability and may sensitize cancer cells to further therapeutic interventions, highlighting its potential as a targeted anticancer strategy.

## 3 Conclusion

In this study, based on a screening campaign of an in‐house library comprising compounds with a diverse range of structures, we successfully identified the triazole scaffold as a promising tool for exploring the antiapoptotic BAG3 protein. Through stepwise structural refinement, guided by biophysical analyses and computational studies, we discovered a compelling compound (**2**) capable of interacting with the BAG domain of the protein while exhibiting significant cytotoxic activity. Given the limited number of known BAG3 modulators and the challenges associated with their discovery, this finding represents a valuable contribution to expanding the toolbox for targeting this multifaceted protein, which plays a crucial role in several cancer‐related pathways and holds great therapeutic potential. Furthermore, and most importantly, the insights gained from in silico studies pave the way for more in‐depth and systematic investigations aimed at developing more potent and selective compounds as potential drug candidates.

## 4 Experimental Section

### t‐LiP‐MS (Targeted‐Limited Proteolysis‐Mass Spectrometry) Experiment on BAG3

To develop the t‐LiP‐MS experiment, MRM methods were optimized by selecting tryptic peptides for BAG3 (UniProt Accession: O95817) using the Human PeptideAtlas data repository in combination with SRMAtlas for their daughter ion identification. First, the three best transitions for each peptide were selected (obtaining a list of 57 transitions) and tested on a tryptic‐digested HeLa cell lysate, following the protocol of Capuano et al., 2024.^[^ ^41^ ^]^ For chromatographic separation, 15 µL of peptide mixture samples (dissolved in 10% formic acid (FA) to a final concentration of 2 µg µL^−1^) were injected and separated on an Aeris Peptide XB C18 column (50 × 2.1 mm, 3.6 μm, 100 Å, Phenomenex, Torrance, USA) using a gradient from 5% to 95% B in 12 min (A: 0.1% FA in H_2_O, B: 0.1% FA in CH_3_CN). QTrap parameters were as follows: curtain gas (CUR) = 30; ion‐spray voltage (IS) = 5500; temperature (TEM) = 250 °C; ion source gas 1 (GS1) = 25; ion source gas 2 (GS2) = 25; declustering potential (DP) = 80; entrance potential (EP) = 15; collision cell exit potential (CXP) = 12. Data acquisition and processing were performed using Analyst software 1.6.2 (AB Sciex, Foster City, CA, USA). At the end of the preliminary experiment, 14 best transitions were selected for the final MRM method.

For the t‐Lip‐MS experiment, HeLa cell lysate (3µ µL^−1^) was divided into aliquots and incubated for 1 hr either with DMSO (control sample) or **SP18** (100 µM in DMSO) at room temperature under shaking (Mini‐Rotator, Biosan). Samples were then first subjected to limited proteolysis with the nonspecific enzyme subtilisin (enzyme‐to‐proteins ratio of 1:1500 w/w) for 30 min, at room temperature and under continuous shaking (500 rpm, Thermomixer, Biosan) and then extensively digested with the specific protease trypsin (enzyme to proteins ratio of 1:100 w/w, overnight, room temperature, 300 RPM).^[^ ^42^ ^]^ Thus, MRM‐MS analysis was performed in triplicate using the BAG3 MRM methods already optimized (as previously described). Analyst Software was used to measure the area of each tryptic peptide peak. Fold change (Fc) (the ratio between the peak area in the treated sample and control sample) was calculated for each peptide, and only those with Fc ≥1.5 and *p*‐value ≤0.05 were considered protected.^[^ ^41^ ^]^

### Synthetic Procedure

All starting materials and solvents used in this study were sourced from Merck (Darmstadt, Germany). NMR spectra (^1^H and ^13^C) were recorded on Bruker Avance instruments at field strengths of 400, 500, and 600 MHz, at a temperature of 298 K (Bruker, Milan, Italy). The compounds were dissolved in 0.5 mL of CD_3_OD, CDCl_3_, or (CD_3_)_2_SO (Merck, 99.8 Atom %D). Coupling constants (*J*) were reported in Hertz, and chemical shifts were given in parts per million (ppm) on the delta (*δ*) scale, using the solvent peak as the internal reference. Multiplicities are denoted as follows: s (singlet), d (doublet), dd (doublet of doublets), ddd (doublet of doublet of doublets), td (triplet of doublets), t (triplet), dt (doublet of triplets), q (quartet), and m (multiplet). Mass spectrometry was performed using an LTQ Orbitrap XL mass spectrometer (Thermo Scientific, Monza, Italy). Reaction monitoring was carried out on silica gel 60 F254 plates (Merck), with spot visualization under UV light at 254 and 365 nm. Semipreparative reversed‐phase HPLC was conducted using an Agilent Technologies 1200 Series system with a Synergi Fusion C18 column (250 × 10.0 mm, 4 μm, 80 Å) at a flow rate of 4 mL min^−1^ (Phenomenex). The binary solvent system (A/B) consisted of 0.1% TFA in water (A) and 0.1% TFA in CH_3_CN (B), with absorbance detection at 240 nm. All biologically tested compounds were confirmed to be >98% pure by HPLC analysis and NMR data. (See Figure S3–S42, Supporting Information).

### General Procedure (A) for the Synthesis of Intermediates **1a**–**1e**

An equimolar amount of the corresponding chlorides **a–** **e** (0.78 mmol, 1 equiv) and sodium azide (0.78 mmol, 1 equiv) were dissolved in dimethylacetamide (2.5 mL). To this mixture, K_2_CO_3_ (2.25 mmol, 2.88 equiv) was added, and the solution was stirred at room temperature for 4 h. The reaction mixture was then diluted with water (5 mL), resulting in a white precipitate. This precipitate was filtered and washed thoroughly with water, yielding the pure azides **1a**–**1e** with a 50%–99% yield. The products were used for the next step without further purification.^[^ ^29^ ^]^

### ((Azidomethoxy)methyl)benzene (**1a**)

White powder; 75% yield.^1^H NMR (400 MHz, CD_3_OD): *δ *= 7.27–7.21 (m, 4H), 7.20–7.15 (m, 1H), 4.72 (s, 2H), 4.52 (s, 2H).

### 2‐(Azidomethyl)anthracene‐9,10‐dione (**1b**)

White powder; 99% yield.^1^H NMR (400 MHz, CDCl_3_): *δ *= 8.30 (t, *J* = 6.5 Hz, 3H), 8.23 (s, 1H), 7.81 (dd, *J* = 5.9, 3.3 Hz, 2H), 7.75 (d, *J* = 8.0 Hz, 1H), 4.57 (s, 2H).

### 1‐(Azidomethyl)naphthalene (**1c**)

White powder; 74% yield. ^1^H NMR (400 MHz, CDCl_3_): *δ* = 8.19 (d, *J* = 8.1 Hz, 1H), 8.05 (dd, *J* = 7.6, 1.8 Hz, 1H), 8.00 (dd, *J* = 7.1, 2.4 Hz, 1H), 7.78–7.65 (m, 2H), 7.62–7.54 (m, 2H), 4.81 (s, 2H).

### 1‐(3‐(Azidomethyl)phenyl)‐3,5‐dimethyl‐1H‐pyrazole (**1d**)

Yellow oil; 99% yield. ^**1**^H NMR (400 MHz, CD_3_OD): *δ* = 7.52 (dd, *J* = 8.4, 7.1 Hz, 1H), 7.46–7.37 (m, 3H), 6.08 (s, 1H), 4.45 (s, 2H), 2.27 (d, *J* = 2.6 Hz, 6H).

### 2‐(Azidomethyl)quinoline (**1e**)

Orange oil; 50% yield.^1^H NMR (400 MHz, CDCl_3_): *δ* = 8.06 (dd, *J* = 8.4, 5.6 Hz, 2H), 7.71 (dd, *J* = 8.2, 1.4 Hz, 1H), 7.68–7.62 (m, 1H), 7.46 (td, *J* = 7.3, 6.8, 1.2 Hz, 1H), 7.34 (d, *J* = 8.4 Hz, 1H), 4.59 (s, 2H).

### General Procedure (B) for the Synthesis of Intermediates **2f**–**2i** and Compound **3**

A 25 mL round‐bottom flask was charged with a mixture of propargyl alcohol (1.86 mmol, 2 equiv), the corresponding carboxylic acids (**f–** **i**) (0.93–mmol, 1–equiv) *N*,*N*’‐diisopropylcarbodiimide (1.12 mmol, 1.2 equiv), 4‐dimethylaminopyridine (0.11 mmol, 0.12 equiv), and dichloromethane (6 mL). The reaction mixture was stirred at room temperature for 16 h while monitoring its progress by TLC. After completion of the reaction, the mixture was cooled to room temperature and diluted with ethyl acetate (20 mL). The organic phase was washed with an aqueous solution of citric acid (10%, 2 × 10 mL). The organic layer was then dried over anhydrous Na_2_SO_4_, filtered, and concentrated under reduced pressure to afford the crude product **2f**–**2i** and compound **10**. The products were used for the next step without further purification.^[^ ^30^ ^]^

### Prop‐2‐yn‐1‐yl Benzoate (**2f**)

White powder; 90% yield. ^1^H NMR (400 MHz, CD_3_OD) *δ* 8.85–8.81 (m, 1H), 8.52 (ddd, *J* = 8.3, 2.4, 1.1 Hz, 1H), 8.45–8.41 (m, 1H), 7.81 (t, *J* = 8.0 Hz, 2H), 5.04 (d, *J* = 2.5 Hz, 2H), 3.05 (t, *J* = 2.5 Hz, 1H).

### Prop‐2‐yn‐1‐yl 2‐chloroisonicotinate (**2g**)

White powder; 63% yield. ^1^H NMR (400 MHz, CD_3_OD): *δ* = 8.60 (dd, *J* = 5.1, 0.8 Hz, 1H), 7.96 (dd, *J* = 1.4, 0.8 Hz, 1H), 7.90 (dd, *J* = 5.1, 1.4 Hz, 1H), 5.02 (d, *J* = 2.5 Hz, 2H), 3.07 (t, *J* = 2.5 Hz, 1H).

### Prop‐2‐yn‐1‐yl 4‐amino‐3‐bromobenzoate (**2h**)

Yellowish powder; 70% yield. ^1^H NMR (400 MHz, CD_3_OD): *δ* = 7.93 (d, *J* = 2.0 Hz, 1H), 7.64 (dd, *J* = 8.5, 1.9 Hz, 1H), 6.72 (d, *J* = 8.5 Hz, 1H), 4.78 (d, *J* = 2.5 Hz, 2H), 2.85 (t, *J* = 2.5 Hz, 1H).

### Prop‐2‐yn‐1‐yl 2‐iodobenzoate (**2i**)

White powder; 85% yield. ^**1**^H NMR (400 MHz, CD_3_OD): *δ* = 7.96 (dd, *J* = 7.9, 1.2 Hz, 1H), 7.70 (dd, *J* = 7.8, 1.7 Hz, 1H), 7.41 (td, *J* = 7.6, 1.2 Hz, 1H), 7.17 (td, *J* = 7.7, 1.7 Hz, 1H), 4.86 (d, *J* = 2.5 Hz, 2H), 2.92 (t, *J* = 2.5 Hz, 1H).

### (1‐(Naphthalen‐1‐ylmethyl)‐1H‐1,2,3‐triazol‐4‐yl)methyl 3‐nitrobenzoate (**3**)

White powder; 51% yield after HPLC purification. RP‐HPLC *t*_R_ = 30 min, gradient condition: from 5% H_2_O ending to 100% CH_3_CN in 40 min, flow rate of 4 mL min^−1^, *λ *= 240 nm. ^1^H NMR (400 MHz, CD_3_OD): *δ* = 8.75 (t, *J* = 2.0 Hz, 1H), 8.47 (ddd, *J* = 8.3, 2.4, 1.1 Hz, 1H), 8.35 (dt, *J* = 7.8, 1.4 Hz, 1H), 8.14–8.10 (m, 1H), 8.02 (s, 1H), 7.97–7.92 (m, 2H), 7.74 (t, *J* = 8.0 Hz, 1H), 7.59–7.50 (m, 4H), 6.13 (s, 2H), 5.46 (s, 3H). ^13^C NMR (101 MHz, CD_3_OD): *δ *= 164.1, 152.4, 148.3, 134.8, 134.0, 131.3, 131.0, 130.3, 129.8, 129.5, 128.6, 127.6, 127.3, 126.6, 125.9, 125.1, 124.9, 123.8, 122.6, 58.0, 51.7. ESI‐HRMS: calculated for C_21_H_16_N_4_O_4_ 388.1172, found *m*/*z* = 411.1017 [M + Na]^+^.

### General Procedure (C) for the Synthesis of **1**, **2**, **4**–**10** and Intermediate **1c′**

An equimolar amount of Ugi azides **1a**–**1e** (0.40 mmol, 1 equiv) and intermediates **2f**–**2i** (0.40 mmol, 1 equiv) were dissolved in the minimum amount of DMSO (0.2 mL). To this solution, a mixture of t‐BuOH/H_2_O (2:1; 2 mL:1 mL), CuSO_4_·5H_2_O (1.28 mmol, 3.2 equiv), and sodium ascorbate (1.20 mmol, 3 equiv) was added. The reaction mixture was stirred at room temperature for 12 h and then poured into cold water. The precipitated click product was filtered, washed with water, and dried under vacuum, yielding compounds **1**, **2**, **4**–**10** and intermediate **1c’** in pure form.^[^ ^29^ ^]^

### (1‐((Benzyloxy)methyl)‐1H‐1,2,3‐triazol‐4‐yl)methyl 3‐nitrobenzoate (**1**)

White powder; 66% yield after HPLC purification. RP‐HPLC *t*_R_ = 26 min, gradient condition: from 5% H_2_O ending to 100% CH_3_CN in 40 min, flow rate of 4 mL min^−1^, *λ *= 240 nm. ^1^H NMR (400 MHz, CD_3_OD): *δ* = 8.83 (t, *J* = 2.0 Hz, 1H), 8.50 (ddd, *J* = 8.3, 2.4, 1.1 Hz, 1H), 8.43 (dt, *J* = 7.8, 1.4 Hz, 1H), 8.30 (s, 1H), 7.79 (t, *J* = 8.0 Hz, 1H), 7.34–7.23 (m, 5H), 5.85 (s, 2H), 5.55 (s, 2H), 4.62 (s, 2H). ^13^C NMR (101 MHz, CD_3_OD): *δ *= 165.3, 148.2, 144.9, 136.7, 134.9 (2C), 131.9, 129.9, 127.7 (3C), 127.3 (2C), 123.8, 121.1, 77.9, 71.0, 57.8. ESI‐HRMS: calculated for C_18_H_16_N_4_O_5_ 368.1121, found *m*/*z* = 391.1001 [M + Na]^+^.

### (1‐((9,10‐Dioxo‐9,10‐dihydroanthracen‐2‐yl)methyl)‐1H‐1,2,3‐triazol‐4‐yl)methyl 3‐nitrobenzoate (**2**)

White powder; 32% yield after precipitation in methanol. ^1^H NMR (400 MHz, (CD_3_)_2_SO): *δ *= 8.61 (t, *J* = 2.0 Hz, 1H), 8.52–8.47 (m, 2H), 8.37 (dt, *J* = 7.8, 1.3 Hz, 1H), 8.26–8.17 (m, 3H), 8.13 (d, *J* = 1.8 Hz, 1H), 7.97–7.91 (m, 2H), 7.88–7.81 (m, 2H), 5.90 (s, 2H), 5.51 (s, 2H). ^13^C NMR (101 MHz, (CD_3_)_2_SO): *δ *= 182.7, 182.6, 164.2, 148.4, 143.0, 142.4, 135.8, 135.1, 135.1, 134.2, 133.8, 133.5 (2C), 133.2, 131.4, 131.3, 128.4, 128.0, 127.3 (2C), 126.4, 126.2, 124.1, 59.1, 52.7. ESI‐HRMS: calculated for C_25_H_16_N_4_O_6_ 468.1070 found *m*/*z* = 491.0945 [M + Na]^+^.

### (1‐(3‐(3,5‐Dimethyl‐1H‐pyrazol‐1‐yl)benzyl)‐1H‐1,2,3‐triazol‐4‐yl)methyl 3‐nitrobenzoate (**4**)

White powder; 32% yield after HPLC purification. RP‐HPLC *t*_R_ = 27 min, gradient condition: from 5% H_2_O ending to 100% CH_3_CN in 40 min, flow rate of 4 mL min^−1^, *λ *= 240 nm. ^1^H NMR (600 MHz, CD_3_OD): *δ* = 8.67 (t, *J* = 2.0 Hz, 1H), 8.37 (ddd, *J* = 8.3, 2.4, 1.1 Hz, 1H), 8.28 (dt, *J* = 7.8, 1.4 Hz, 1H), 8.13 (s, 1H), 7.65 (t, *J* = 8.0 Hz, 1H), 7.44 (t, *J* = 7.8 Hz, 1H), 7.33 (ddt, *J* = 7.7, 3.2, 1.1 Hz, 2H), 7.30 (t, *J* = 1.9 Hz, 1H), 5.96 (s, 1H), 5.62 (s, 2H), 5.41 (s, 2H), 2.13–2.11 (m, 6H). ^13^C NMR (151 MHz, CD_3_OD): *δ* = 164.1, 149.2, 148.3, 142.7, 140.6, 139.7, 136.8, 134.9, 131.4, 129.9, 129.6, 127.3, 127.3, 125.0, 124.8, 124.4, 123.8, 106.8, 58.0, 53.00 11.7, 10.7. ESI‐HRMS**:** calculated for C_22_H_20_N_6_O_4_ 432.1546, found *m*/*z* = 455.1530 [M + Na]^+^.

### (1‐(Quinolin‐2‐ylmethyl)‐1H‐1,2,3‐triazol‐4‐yl)methyl 3‐nitrobenzoate (**5**)

White powder; 86% yield after HPLC purification. RP‐HPLC *t*_R_ = 24 min, gradient condition: from 5% H_2_O ending to 100% CH_3_CN in 40 min, flow rate of 4 mL min^−1^, *λ* = 240 nm. ^1^H NMR (400 MHz, (CD_3_)_2_SO) *δ* 8.63–8.61 (m, 1H), 8.51 (dd, *J* = 8.2, 2.2 Hz, 1H), 8.47 (s, 1H), 8.42 (d, *J* = 8.5 Hz, 1H), 8.38 (d, *J* = 7.7 Hz, 1H), 8.00 (d, *J* = 8.0 Hz, 1H), 7.95 (d, *J* = 8.4 Hz, 1H), 7.85 (t, *J* = 8.0 Hz, 1H), 7.78 (t, *J* = 7.7 Hz, 1H), 7.63 (t, *J* = 7.5 Hz, 1H), 7.44 (d, *J* = 8.5 Hz, 1H), 5.96 (s, 2H), 5.52 (s, 2H). ^13^C NMR (151 MHz, (CD_3_)_2_SO): *δ* = 164.3, 158.4, 155.9, 148.4, 147.4, 142.1, 138.1, 135.8, 131.4, 131.3, 130.6, 129.0, 128.4, 127.6, 127.4, 126.7, 124.1, 120.5, 59.2, 55.5. ESI‐HRMS: calculated for C_20_H_15_N_5_O_4_ 389.1124, found *m*/*z* = 412.0997 [M + Na]^+^.

### (1‐(Quinolin‐2‐ylmethyl)‐1H‐1,2,3‐triazol‐4‐yl)methyl 2‐chloroisonicotinate (**6**)

White powder; 98% yield after HPLC purification. RP‐HPLC *t*_R_ = 22 min, gradient condition: from 5% H_2_O ending to 100% CH_3_CN in 40 min, flow rate of 4 mL min^−1^, *λ* = 240 nm. ^1^H NMR (400 MHz, (CD_3_)_2_SO): *δ* = 8.64 (t, *J* = 4.0 Hz, 1H), 8.48 (s, 1H), 8.42 (d, *J* = 8.5 Hz, 1H), 7.98 (dd, *J* = 16.0, 8.3 Hz, 2H), 7.86 (dd, *J* = 9.3, 4.9 Hz, 2H), 7.78 (t, *J* = 7.6 Hz, 1H), 7.63 (t, *J* = 7.6 Hz, 1H), 7.44 (d, *J* = 8.4 Hz, 1H), 5.96 (s, 2H), 5.50 (s, 2H). ^13^C NMR (101 MHz, (CD_3_)_2_SO): *δ* = 163.6, 155.8, 151.8, 151.7, 147.4, 142.0, 140.6, 138.1, 130.6, 129.1, 128.4, 127.6, 127.4, 126.7, 123.8, 122.6, 120.5, 59.5, 55.5. ESI‐HRMS: calculated for C_19_H_14_ClN_5_O_2_ 379.0836, found *m*/*z* = 402.0768 [M + Na]^+^.

### (1‐(Quinolin‐2‐ylmethyl)‐1H‐1,2,3‐triazol‐4‐yl)methyl 4‐amino‐3‐bromobenzoate (**7**)

Yellowish powder; 65% yield after HPLC purification. RP‐HPLC *t*_R_ = 20 min, gradient condition: from 5% H_2_O ending to 100% CH_3_CN in 40 min, flow rate of 4 mL min^−1^, *λ* = 240 nm. ^1^H NMR (600 MHz, CD_3_OD): *δ* = 8.41 (d, *J* = 8.5 Hz, 1H), 8.27 (s, 1H), 8.04–8.01 (m, 2H), 7.97 (dd, *J* = 8.1, 1.4 Hz, 1H), 7.81 (ddd, *J* = 8.4, 6.9, 1.5 Hz, 1H), 7.73 (dd, *J* = 8.5, 2.0 Hz, 1H), 7.65 (ddd, *J* = 8.1, 6.8, 1.1 Hz, 1H), 7.44 (dd, *J* = 8.5, 3.2 Hz, 1H), 6.79 (d, *J* = 8.6 Hz, 1H), 5.96 (s, 2H), 5.41 (s, 2H). ^13^C NMR (151 MHz, CD_3_OD): *δ* = 165.42, 154.72, 150.34 (2C), 146.87, 138.39, 134.16, 130.31, 129.92, 127.76, 127.73, 127.70, 127.08, 119.60 (2C), 118.12, 113.59, 106.33, 56.96, 55.04. ESI‐HRMS: calculated for C_20_H_16_BrN_5_O_2_ 437.0487, found *m*/*z* = 460.0426 [M + Na]^+^.

### (1‐(Quinolin‐2‐ylmethyl)‐1H‐1,2,3‐triazol‐4‐yl)methyl 2‐iodobenzoate (**8**)

White powder; 38% yield after HPLC purification. RP‐HPLC *t*_R_ = 27 min, gradient condition: from 5% H_2_O ending to 100% CH_3_CN in 40 min, flow rate of 4 mL min^−1^, *λ* = 240 nm. ^1^H NMR (500 MHz, (CD_3_)_2_SO): *δ* = 8.47–8.40 (m, 2H), 7.99 (dt, *J* = 20.8, 8.1 Hz, 3H), 7.79 (t, *J* = 7.6 Hz, 1H), 7.73–7.67 (m, 1H), 7.64 (t, *J* = 7.4 Hz, 1H), 7.52 (dt, *J* = 15.0, 7.6 Hz, 1H), 7.43 (t, *J* = 7.6 Hz, 1H), 7.29 (t, *J* = 7.6 Hz, 1H), 5.96 (s, 2H), 5.44 (d, *J* = 4.3 Hz, 2H). ^13^C NMR (126 MHz, (CD_3_)_2_SO): *δ* = 166.6, 155.9, 147.4, 141.1, 138.0, 135.7, 133.6, 130.8, 130.6, 129.7, 129.3, 129.1, 128.8, 128.4, 127.6, 127.4, 120.4, 94.7, 59.0, 55.5. ESI‐HRMS: calculated for C_20_H_15_IN_4_O_2_ 470.0240, found *m*/*z* = 493.0114 [M + Na]^+^.

### (1‐(3‐(3,5‐Dimethyl‐1H‐pyrazol‐1‐yl)benzyl)‐1H‐1,2,3‐triazol‐4‐yl)methyl 4‐amino‐3‐bromobenzoate (**9**)

Yellowish powder, 80% yield after HPLC purification. RP‐HPLC *t*_R_ = 25 min, gradient condition: from 5% H_2_O ending to 100% CH_3_CN in 40 min, flow rate of 4 mL min^−1^, *λ* = 240 nm. ^1^H NMR (400 MHz, (CD_3_)_2_SO) *δ* 8.35 (s, 1H), 7.87 (d, *J* = 2.0 Hz, 1H), 7.63 (dd, *J* = 8.5, 2.0 Hz, 1H), 7.53–7.43 (m, 3H), 7.33 (dt, *J* = 7.2, 1.6 Hz, 1H), 6.80 (d, *J* = 8.5 Hz, 1H), 6.06 (s, 1H), 5.69 (s, 2H), 5.30 (s, 2H), 2.25 (s, 3H), 2.17 (s, 3H). ^13^C NMR (101 MHz, (CD_3_)_2_SO): *δ* = 164.8, 151.0, 148.5, 143.0, 140.3, 139.5, 137.5, 134.3, 130.5, 129.8, 128.8, 126.9, 125.5, 123.9, 117.5, 114.6, 107.8, 106.2, 57.8, 52.8, 13.7, 12.6. ESI‐HRMS: calculated for C_22_H_21_BrN_6_O_2_ 480.0909, found *m*/*z* = 503.0787 [M + Na]^+^.

### (1‐(Naphthalen‐1‐ylmethyl)‐1H‐1,2,3‐triazol‐4‐yl)methyl 4‐amino‐3‐bromobenzoate (**10**)

Yellowish powder; 82% yield after HPLC purification. RP‐HPLC *t*_R_ = 28 min, gradient condition: from 5% H_2_O ending to 100% CH_3_CN in 40 min, flow rate of 4 mL min^−1^, *λ* = 240 nm. ^1^H NMR (400 MHz, CD_3_OD): *δ *= 8.14–8.08 (m, 1H), 7.98–7.91 (m, 4H), 7.67 (dd, *J* = 8.5, 2.0 Hz, 1H), 7.59–7.47 (m, 4H), 6.77 (d, *J* = 8.5 Hz, 1H), 6.11 (s, 2H), 5.31 (s, 2H). ^13^C NMR (151 MHz, CD_3_OD): *δ* = 165.3, 150.3, 134.1, 134.0, 131.0, 130.4, 129.9, 129.4, 128.5 (2C), 127.5, 126.6, 125.9, 125.1, 124.6, 122.6, 118.1, 113.6, 106.3, 56.9, 51.6. ESI‐HRMS: calculated for C_21_H_17_BrN_4_O_2_ 436.0535, found *m*/*z* = 459.0411 [M + Na]^+^.

### (1‐(Naphthalen‐1‐ylmethyl)‐1H‐1,2,3‐triazol‐4‐yl)methanol (**1c′**)

White powder, 73% yield. ^1^H NMR (400 MHz, CD_3_OD): *δ* = 8.15–8.11 (m, 1H), 7.97–7.91 (m, 2H), 7.77 (s, 1H), 7.59–7.50 (m, 4H), 6.10 (s, 2H), 4.63 (s, 2H).

### SPR

Recombinant human BAG3 protein (Bcl2‐associated athanogene 3) was obtained from Novus Biologicals (Littleton, Colorado), while the BAG3 domain (BAG3‐BD) was sourced from ARETA International S.r.l. (Gerenzano, Italy). We used (*Z*)‐ethyl 2‐(5‐(3,4‐dihydroxybenzylidene)‐2,4‐dioxothiazolidin‐3‐yl)acetate (**LK4**), a BAG3 inhibitor previously identified by our group.^[^ ^13^ ^]^ SPR spectroscopy was performed using a Biacore T200 optical biosensor with CM5 sensor chips (Cytiva, Marlborough, USA) to assess the affinity of synthesized molecules for BAG3 and BAG‐BD. BAG3 and BAG‐BD were immobilized on CM5 sensor chip using standard amine‐coupling protocol.^[^ ^15^ ^,^ ^42^ ^,^ ^43^ ^]^ BAG3 protein (0.067 μg μL^−1^ in 10 mM CH_3_COONa, pH 4.5) was immobilized at a flow rate of 10 μL min^−1^, achieving a surface density of 14 kRU, while BAG3‐BD (0.38 μg μL^−1^ in 10 mM CH_3_COONa, pH 4.5) reached a density of 4 kRU. For the experiments, BAG3 and BAG‐BD surfaces, as well as an unmodified reference surface, were prepared for simultaneous analysis. Compounds **1**–**10** and **LK4** were initially dissolved to 10 mM in 100% DMSO, then diluted 1:20 (v/v) in PBS‐P buffer (0.2 M phosphate buffer, 27 mM KCl, 1.37 M NaCl, 0.5% surfactant P20) resulting in a final DMSO concentration of 5.0%. The compounds were injected in a concentration series (1:2 dilution, 10 different concentrations) ranging from 0 to 100 μM, using 96‐well plates. SPR experiments were conducted at 25 °C with a flow rate of 20 μL min^−1^, featuring a 90 s association phase followed by a 400 s dissociation phase. Changes in mass, recorded as resonance units (RU), were used to quantify binding responses, while *K*_D_ values were determined using Biaevaluation software by globally fitting the double‐referenced association and dissociation data to a 1:1 interaction model. (See Figure S43–S56, Supporting Information).

### Computational Details

Molecular modeling studies were performed using software belonging to the Schrödinger Suite. Maestro was used for drawing the structures of the test compounds and to manage the different steps required for the in silico experiments (Schrödinger Release 2021‐1: Maestro, Schrödinger, LLC, New York, NY, 2021). First, the murine solution NMR BAG3 structure (PDB: 1UK5) was retrieved from the Protein Data Bank. The PDB entry contains an ensemble of 20 conformers obtained by solution NMR spectroscopy. Model 1, the lowest‐energy conformer and the first in the ensemble, was selected as the representative structure for all analyses and molecular docking calculations. BAG3 protein was then processed using the Protein Preparation Wizard workflow (Schrödinger Release 2021‐1: Protein Preparation Wizard, Epik, Schrödinger, LLC, New York, NY, 2021; Impact, Schrödinger, LLC, New York, NY; Prime, Schrödinger, LLC, New York, NY, 2021). Specifically, water molecules were removed, cap termini were added, bond orders were assigned, and hydrogen atoms were included. The docking grid was generated considering the entire protein surface, given the absence of known BAG3 modulators and the small size of the protein. The final grid center coordinates were set as 0.0 Å (*x*); −8.0 Å (*y*); and 3.0 Å (*z*), with inner and outer box dimensions of 40 and 46 Å, respectively. The 2D structures of **SP18** and of the other test compounds (see paragraph 2.4.1) were drawn employing Maestro 2D sketcher tool and subsequently prepared with LigPrep tool (Schrödinger Release 2021‐1: LigPrep, Schrödinger, LLC, New York, NY, 2021). All possible tautomers and protonation states at pH = 7.4 ± 1.0 were generated, followed by energy minimization with the OPLS2005 force field. Molecular docking calculations were then performed using Glide at the Standard Precision (SP) level, saving a maximum of 20 docking poses (Schrödinger Release 2021‐1: Glide, Schrödinger, LLC, New York, NY, 2021) (See Figure 4 and Figure S2, Supporting Information).

### Cell Culture

Human cell lines of lung adenocarcinoma (A549), cervical carcinoma (HeLa), malignant melanoma (A375), breast adenocarcinoma (MCF7), and immortalized human keratinocytes (HaCaT) were cultured in high‐glucose DMEM supplemented with FBS (10%, v/v), glutamine (2 mM L^−1^), penicillin (100 U mL^−1^), and streptomycin (100 mg mL^−1^). Cells were maintained at 37 °C in a humidified 5% CO_2_ atmosphere and subcultured every 2 days to maintain logarithmic growth. The human cell lines were kindly donated by Prof. Ornella Moltedo, Department of Pharmacy, University of Salerno.^[^ ^15^ ^,^ ^44^ ^]^

### Cell Viability

Cell viability was evaluated using a colorimetric assay to measure mitochondrial metabolic activity through the reduction of 3‐[4,5‐dimethylthiazol‐2,5‐diphenyl‐2H‐tetrazolium bromide (MTT) to purple formazan. Stock solutions of **SP18** and compounds **1**, **2**, **5**–**8** (50 mM in DMSO) were stored at −20 °C in the dark and diluted immediately before addition to the sterile culture medium. The final concentration of DMSO for the tested compounds was 0.1%, which was consistent with the percentage of DMSO used in the negative control. A549 (3.5 × 10³ cells well^−1^), HeLa (3.5 × 10³ cells well^−1^), A375 (5.0 × 10³ cells well^−1^), and MCF7 (3.5 × 10³ cells well^−1^) cells were seeded in triplicate in 96‐well plates. After 24 h to allow for attachment, cells were treated with various concentrations of the compounds (10 and 50 µM), using 0.1% (v/v) DMSO as a negative control and 10% (v/v) DMSO as a positive control following treatment, 20 μL of MTT solution (5 mg mL^−1^ in PBS) were added to each well, and cells were incubated for 3 h at 37 °C. Formazan crystals were then dissolved in 100 μL of a buffer containing 50% (v/v) *N*,*N*‐dimethylformamide and 20% SDS (pH 4.5), and absorbance was measured at 570 nm using a Multiskan GO Microplate Spectrophotometer (Thermo Fisher Scientific, USA).^[^ ^44^ ^]^ For **SP18** and compound **2**, which showed significant antiproliferative activity in HeLa cells, dose–response proliferation assays were conducted at seven concentrations (1.56–100 μM) to determine IC_50_ values at 48 and 72 h. IC_50_ values, defined as the concentration causing 50% inhibition of cell survival, were calculated and compared to DMSO‐treated controls using GraphPad Prism 8.0 software via nonlinear regression. IC_50_ values for **SP18** and compound **2** were also determined in HaCaT cells (5.0 × 10³ cells well^−1^) after 48 or 72 h of treatment.^[^ ^15^ ^]^

### Apoptosis and Cell Cycle

Hypodiploid nuclei were analyzed by propidium iodide (PI) staining using flow cytometry. HeLa cells (1 × 10^5^ cells well^−1^) were cultured in 12‐well plates and allowed to adhere for 24 h. The medium was then refreshed, and cells were treated with **SP18** (0.75, 1.5, 3, 6, and 12 µM) for 48 h or compound **2** (10, 30, and 50 µM) for 72 h, with 0.1% (v/v) DMSO as a control. The final concentration of DMSO for the tested compounds was 0.1%, which is consistent with the percentage of DMSO used in the negative control. Following treatment, the culture medium was replaced, and cells were washed twice with PBS before being resuspended in 500 μL of a solution containing 50 mg L^−1^ PI, 0.1% (w/v) sodium citrate, and 0.1% Triton X‐100. To include both dead and live cells in the analysis, the culture medium and PBS washes were centrifuged, and the resulting cell pellets were combined with the main cell suspension. Cells were then incubated at 4 °C for 30 min in the dark, and nuclei were analyzed using a Becton Dickinson FACScan flow cytometer (Franklin Lakes, USA) with Cell Quest software. DNA content was recorded, and cellular debris was excluded by adjusting the forward scatter threshold. The percentage of cells in the hypodiploid region (sub‐G0) and those in the G1, S, and G2 phases were calculated.^[^ ^45^ ^]^ (See Figure S57–S58, Supporting Information).

### Annexin V‐FITC/PI Staining

Apoptosis in HeLa cells was evaluated using Annexin V‐FITC/PI staining. Hela cells (20 × 10^3^) were seeded into 24‐well plates and incubated for 72 h with compound **2**. The final concentration of DMSO for the tested compound was 0.1%, which was consistent with the percentage of DMSO used in the negative control. After treatments, cells were resuspended in a 100 μL of assay buffer per well, after which 5 μL of Annexin V‐FITC and 1 μL of PI reagents were added. The mixture was then incubated for 20 min at room temperature, as previously showed.^[^ ^28^ ^]^ The cells were analyzed with a Becton Dickinson FACScan flow cytometer using the Cell Quest software, version 4 (Franklin Lakes, NJ, USA).

### Thioflavin T (ThT) Staining

Protein misfolding was analyzed by using Thioflavin T (ThT, Sigma Aldrich, St. Louis, MO, USA) staining.^[^ ^39^ ^]^ HeLa cells (4 × 10^3^ cells well^−1^) were grown in 96‐well plates and allowed to adhere for 24 h. The medium was then replaced, and the cells were treated with compound **2** (3, 6, and 12 µM) for 72 h. The final concentration of DMSO for the tested compound was 0.1%, which is consistent with the percentage of DMSO used in the negative control. After treatments, the culture medium was replaced, cells were washed with PBS and 100 μL well^−1^ of ThT was added (final concentration, 20 µM). After incubation at 37 °C for 20 min in the dark, live cells were analyzed by using ZOE Fluorescent Cell Imager microscope (Biorad, Hercules, California, USA). Magnification, 20×. Scale bar: 100 μm (*N* ≥ 5). Quantitative analyses were performed in end point mode using a PerkinElmer EnSpire multimode plate reader (excitation/emission 450 nm/482 nm).

## Conflict of Interest

The authors declare no conflict of interest.

## Author Contributions

**Dafne Ruggiero**: conceptualization (supporting); formal analysis (supporting); investigation (supporting); methodology (supporting); writing—original draft (supporting). **Eleonora Boccia**: formal analysis (supporting); investigation (supporting); software (supporting); writing—original draft (supporting). **Emis Ingenito**: formal analysis (supporting); investigation (supporting). **Vincenzo Vestuto**: formal analysis (supporting); investigation (supporting). **Gilda D’Urso**: formal analysis (supporting); investigation (supporting). **Alessandra Capuano**: formal analysis (supporting); investigation (supporting). **Agostino Casapullo**: conceptualization (supporting); methodology (supporting). **Stefania Terracciano**: conceptualization (supporting); methodology (supporting). **Giuseppe Bifulco**: methodology (supporting); resources (lead); software (supporting). **Gianluigi Lauro**: conceptualization (lead); methodology (lead); software (lead). **Ines Bruno**: conceptualization (lead); investigation (lead); methodology (lead); supervision (lead); writing—original draft (lead). Dafne Ruggiero and Eleonora Boccia contributed equally to this work.

## Supporting information

Supplementary Material

## Data Availability

The data that support the findings of this study are available from the corresponding author upon reasonable request.
