# Supplementary material for: Identification of a New Interesting BAG3 Modulator Able to Disrupt Cancer‐Related Pathways
Source: ChemMedChem. 2025 Aug 11;20(20):e202500310. doi: 10.1002/cmdc.202500310 (PMC12530843; doi:10.1002/cmdc.202500310)
Supplement: Supplementary file 1 — Supplementary Material [file CMDC-20-e202500310-s001.pdf]

Supporting information

Identification of a New Interesting BAG3 Modulator Able to Disrupt Cancer-Related Pathways

Dafne Ruggiero [a,†], Eleonora Boccia [a,†], Emis Ingenito [a,b], Vincenzo Vestuto [a], Gilda D'Urso [a], Alessandra Capuano [a], Agostino Casapullo [a], Stefania Terracciano [a], Giuseppe Bifulco [a], Gianluigi Lauro [a,*], Ines Bruno [a,*]

[a] Department of Pharmacy, University of Salerno, Via Giovanni Paolo II 132, 84084 Fisciano, Italy
[b] Institute of Molecular and Translational Medicine, Faculty of Medicine and Dentistry and Czech Advanced Technology and Research Institute, Palacky University in Olomouc, Krížkovského 511/8, 779 00 Olomouc, Czech Republic

druggiero@unisa.it; eboccia@unisa.it; eingenito@unisa.it; vvestuto@unisa.it; gidurso@unisa.it; acapuano@unisa.it, casapullo@unisa.it, sterracciano@unisa.it, bifulco@unisa.it, glauro@unisa.it.

† These authors contributed equally to this work.
* Correspondence: brunoin@unisa.it; Tel: +39 089969743; glauro@unisa.it; Tel: +39 089969124.

Summary

Figure S1: Structures of the 60 in-house compounds screened for BAG3 binding via SPR....... 7

Figure S2: 2D representation of ligands at the protein binding site. (a) SP18, (b) compound 1, (c) compound 2, (d) compound 5, (e) compound 6, (f) compound 7, (g) compound 8. 9

Figure S3. Intermediate 1a ^{1}H NMR (400 MHz, CD_3OD) 10

Figure S4. Intermediate 1b ^{1}H NMR (400 MHz, $CDCl_3$) 10

Figure S5. Intermediate 1c ^{1}H NMR (400 MHz, $CDCl_3$)........ 11

Figure S6. Intermediate 1d ^{1}H NMR (400 MHz, CD_3OD) 11

Figure S7. Intermediate 1e ^{1}H NMR (400 MHz, $CDCl_3$)........ 12

Figure S8. Intermediate 1c' ^{1}H NMR (400 MHz, CD_3OD) 12

Figure S9. Intermediate 2f ^{1}H NMR (400 MHz, CD_3OD)........ 13

Figure S10. Intermediate 2g ^{1}H NMR (400 MHz, CD_3OD) 13

Figure S11. Intermediate 2h ^{1}H NMR (400 MHz, CD_3OD)........ 14

Figure S12. Intermediate 2i ^{1}H NMR (400 MHz, CD_3OD) 14

Figure S13. Compound 1 ^{1}H NMR (400 MHz, CD_3OD)........ 15

Figure S14. Compound 1 ^{13}C NMR (101 MHz, CD_3OD) 15

Figure S15. Compound 1 ESI-MS spectrum 16

Figure S16. Compound 2 ^{1}H NMR (400 MHz, $(CD_3)_2SO$) 17

Figure S17. Compound 2 ^{13}C NMR (101 MHz, $(CD_3)_2SO$)........ 17

Figure S18. Compound 2 ESI-MS spectrum 18

Figure S19. Compound 3 ^{1}H NMR (400 MHz, CD_3OD)........ 19

Figure S20. Compound 3 ^{13}C NMR (101 MHz, CD_3OD) 19

Figure S21. Compound 3 ESI-MS spectrum 20

Figure S22. Compound 4 ^{1}H NMR (600 MHz, CD_3OD)........ 21

Figure S23. Compound 4 ^{13}C NMR (151 MHz, CD_3OD) 21

Figure S24. Compound 4 ESI-MS spectrum 22

Figure S25. Compound 5 ^{1}H NMR (400 MHz, $(CD_3)_2SO$) 23

Figure S26. Compound 5 ^{13}C NMR (151 MHz, $(CD_3)_2SO$)........ 23

Figure S27. Compound 5 ESI-MS spectra........ 24

Figure S28. Compound 6 ^{1}H NMR (400 MHz, $(CD_3)_2SO$) 25

Figure S29. Compound 6 ^{13}C NMR (101 MHz, $(CD_3)_2SO$)........ 25

Figure S30. Compound 6 ESI-MS spectrum 26

Figure S31. Compound 7 ^{1}H NMR (600 MHz, CD_3OD)........ 27

Figure S32. Compound 7 ^{13}C NMR (151 MHz, CD_3OD) 27

Figure S33. Compound 7 ESI-MS spectrum 28

Figure S34. Compound 8 ^{1}H NMR (500 MHz, $(CD_3)_2SO$) 29
Figure S35. Compound 8 ^{13}C NMR (126 MHz, $(CD_3)_2SO$) 29
Figure S36. Compound 8 ESI-MS spectrum 30
Figure S37. Compound 9 ^{1}H NMR (400 MHz, $(CD_3)_2SO$) 31
Figure S38. Compound 9 ^{13}C NMR (101 MHz, $(CD_3)_2SO$) 31
Figure S39. Compound 9 ESI-MS spectrum 32
Figure S40. Compound 10 ^{1}H NMR (400 MHz, CD_3OD) 33
Figure S41. Compound 10 ^{13}C NMR (151 MHz, CD_3OD) 33
Figure S42. Compound 10 ESI-MS spectrum 34
Figure S43. Sensorgram of compound 1 on BAG3 full 35
Figure S44. Sensorgram of compound 2 on BAG3 full 35
Figure S45. Sensorgram of compound 5 on BAG3 full 35
Figure S46. Sensorgram of compound 6 on BAG3 full 36
Figure S47. Sensorgram of compound 7 on BAG3 full 36
Figure S48. Sensorgram of compound 8 on BAG3 full 36
Figure S49. Sensorgram of LK4 on BAG3 full 37
Figure S50. Sensorgram of compound 1 on BAG3-BD 38
Figure S51. Sensorgram of compound 2 on BAG3-BD 38
Figure S52. Sensorgram of compound 5 on BAG3-BD 39
Figure S53. Sensorgram of compound 6 on BAG3-BD 39
Figure S54. Sensorgram of compound 7 on BAG3-BD 39
Figure S55. Sensorgram of compound 8 on BAG3-BD 39
Figure S56. Sensorgram of LK4 on BAG3-BD 40
Figure S57: FACS histograms for cell cycle analysis of cells treated with SP18 at various concentrations for 48 hours. 40
Figure S58: FACS histograms for cell cycle analysis of cells treated with compound 2 at various concentrations for 72 hours. 41

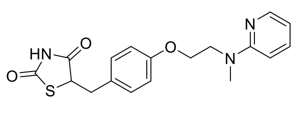
ROSIGLITAZONE

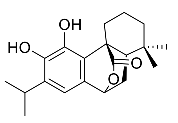
CARNOSOLE

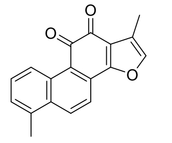
TANSHINONE

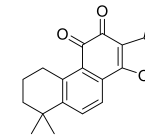
CRYPTOTANSHINONE

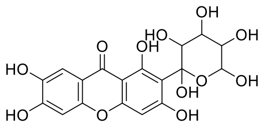
MANGIFERIN

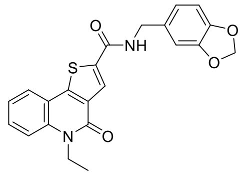
PM1

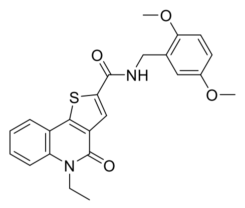
PM2

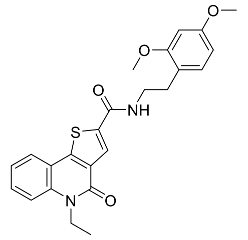
PM3

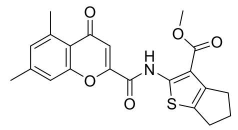
PM4

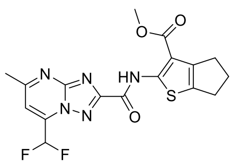
PM5

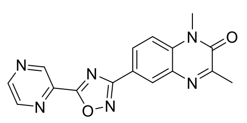
PM6

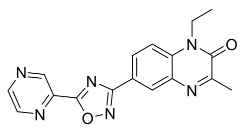
PM7

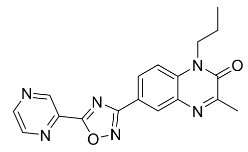
PM8

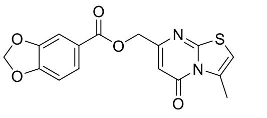
PM9

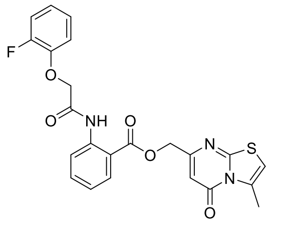
PM10

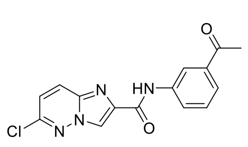
PM11

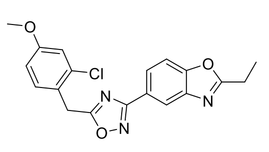
MD1

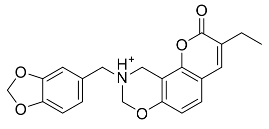
MD2

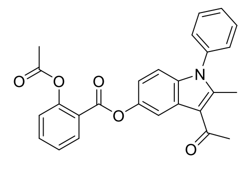
MD3

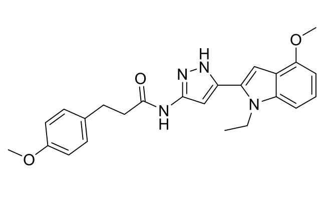
MD4

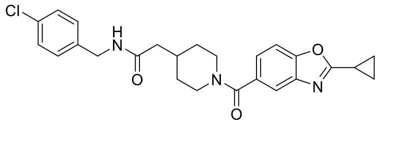
MD5

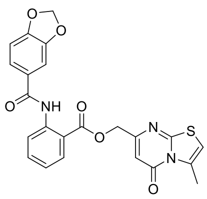

MD6

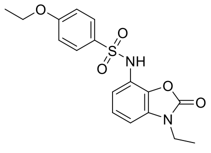

MD7

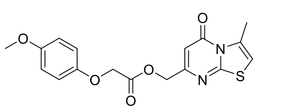

MD8

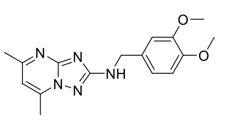

MD9

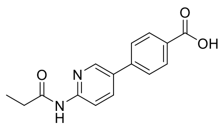

JOC1

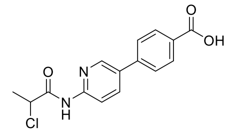

JOC2

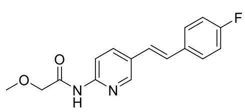

JOC3

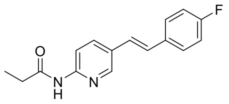

JOC4

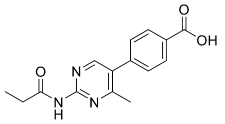

JOC5

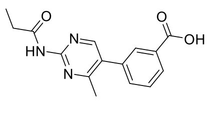

JOC6

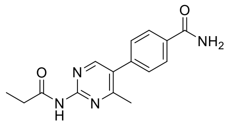

JOC7

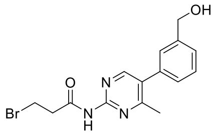

JOC8

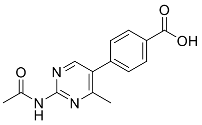

JOC9

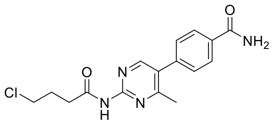

JOC10

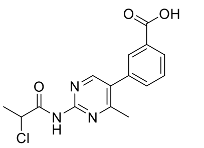

JOC11

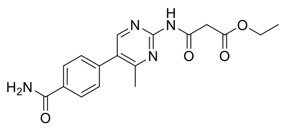

JOC12

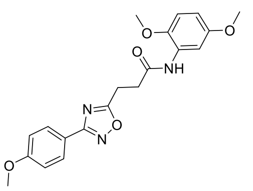

CDH1

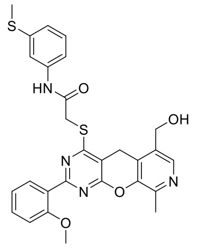

CDH2

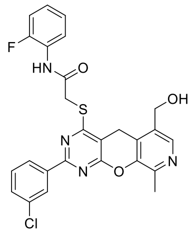

CDH3

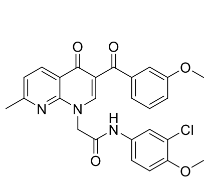

CDH4

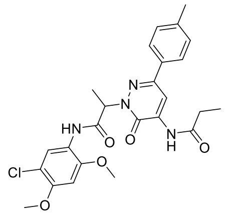

CDH5

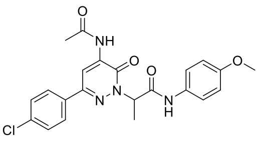

CDH6

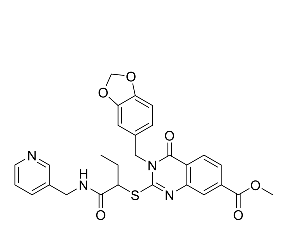

CDH7

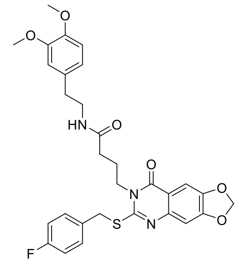

CDH8

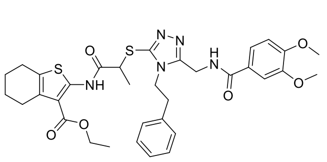

CDH9

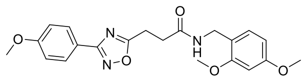

CDH10

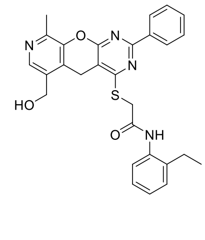

CDH11

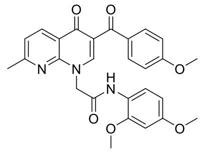

CDH12

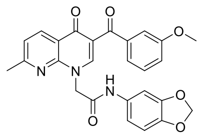

CDH13

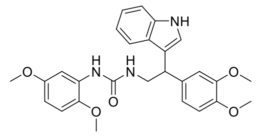

CDH14

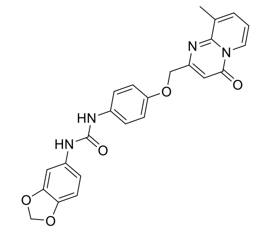

CDH15

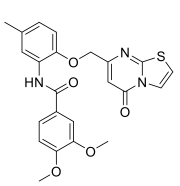

CDH16

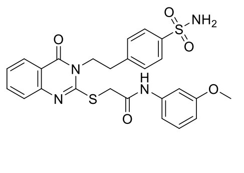

CDH$_{17}$

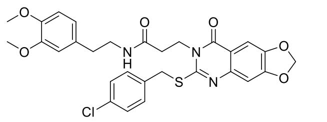

CDH$_{18}$

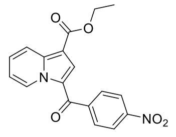

SAL$_1$

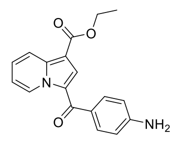

SAL2

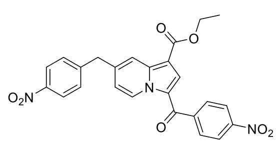

SAL3

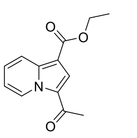

SALBR2

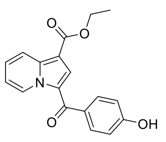

SAL7

Figure S1: Structures of the 60 in-house compounds screened for BAG3 binding via SPR.

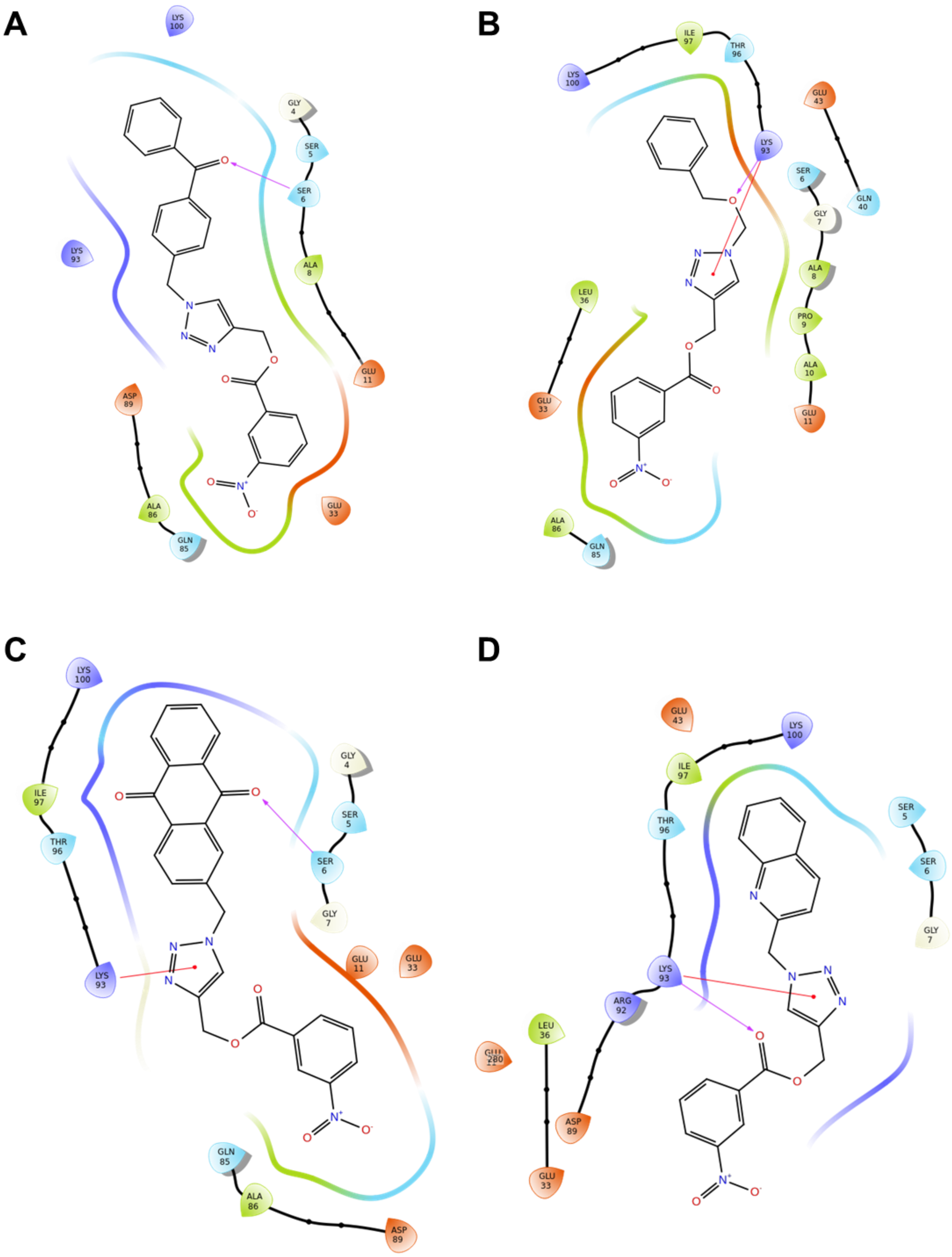
A
LYS 100
GLY 4
SER 5
SER 6
LYS 93
ALA 8
GLU 11
ASP 89
ALA 86
GLN 85
GLU 33
B
ILE 97
THR 96
LYS 100
GLU 43
LYS 93
SER 6
GLN 40
GLY 7
ALA 8
LEU 36
PRO 9
ALA 10
GLU 33
GLU 11
ALA 86
GLN 85
C
LYS 100
GLY 4
ILE 97
SER 5
THR 96
SER 6
GLY 7
GLU 11
GLU 33
LYS 93
GLN 85
ALA 86
ASP 89
D
GLU 43
LYS 100
ILE 97
SER 5
THR 96
SER 6
GLY 7
LYS 93
ARG 92
LEU 36
ASP 89
GLU 33

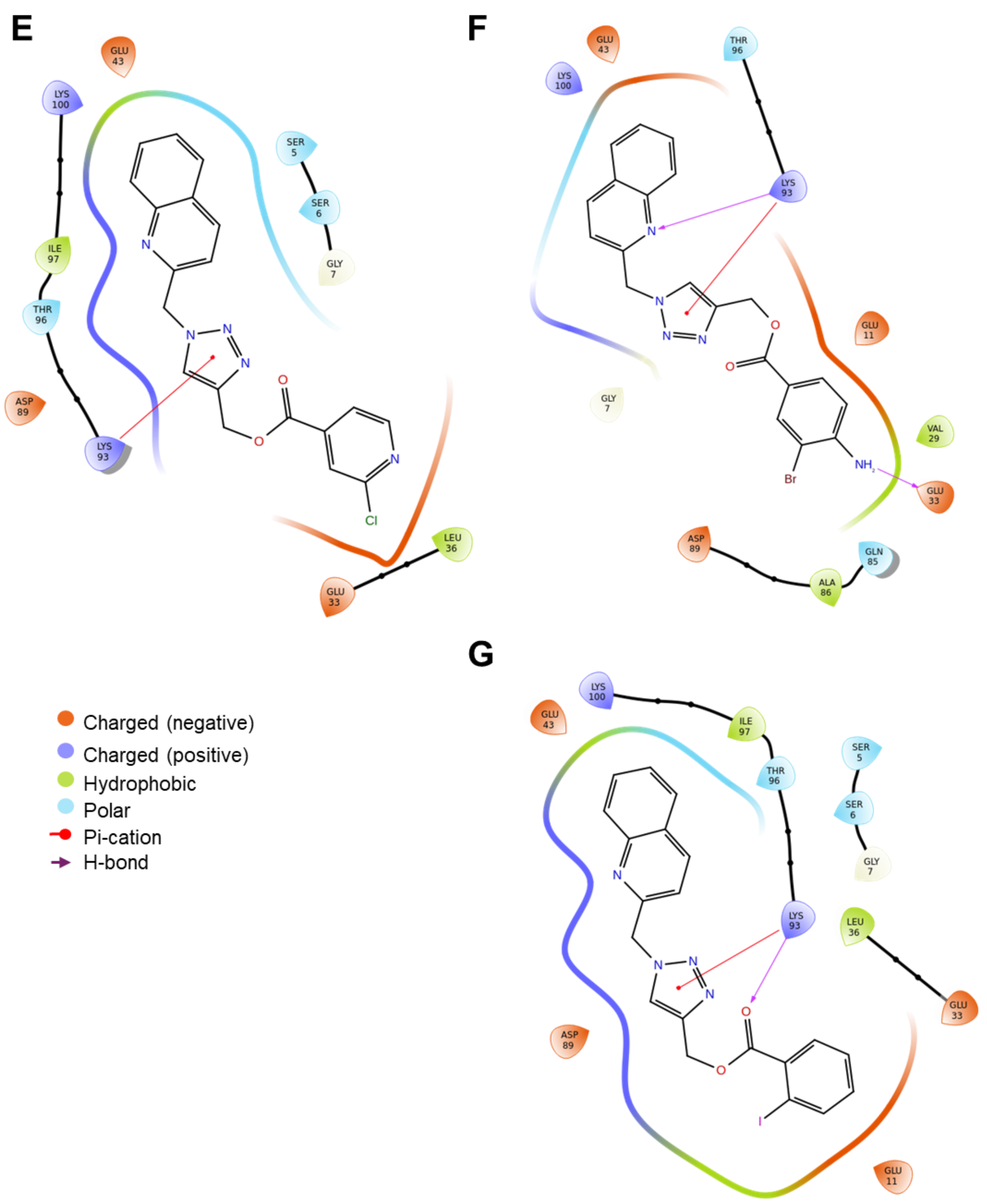

Figure S2: 2D representation of ligands at the protein binding site. (a) SP18, (b) compound 1, (c) compound 2, (d) compound 5, (e) compound 6, (f) compound 7, (g) compound 8.

INTERMEDIATES

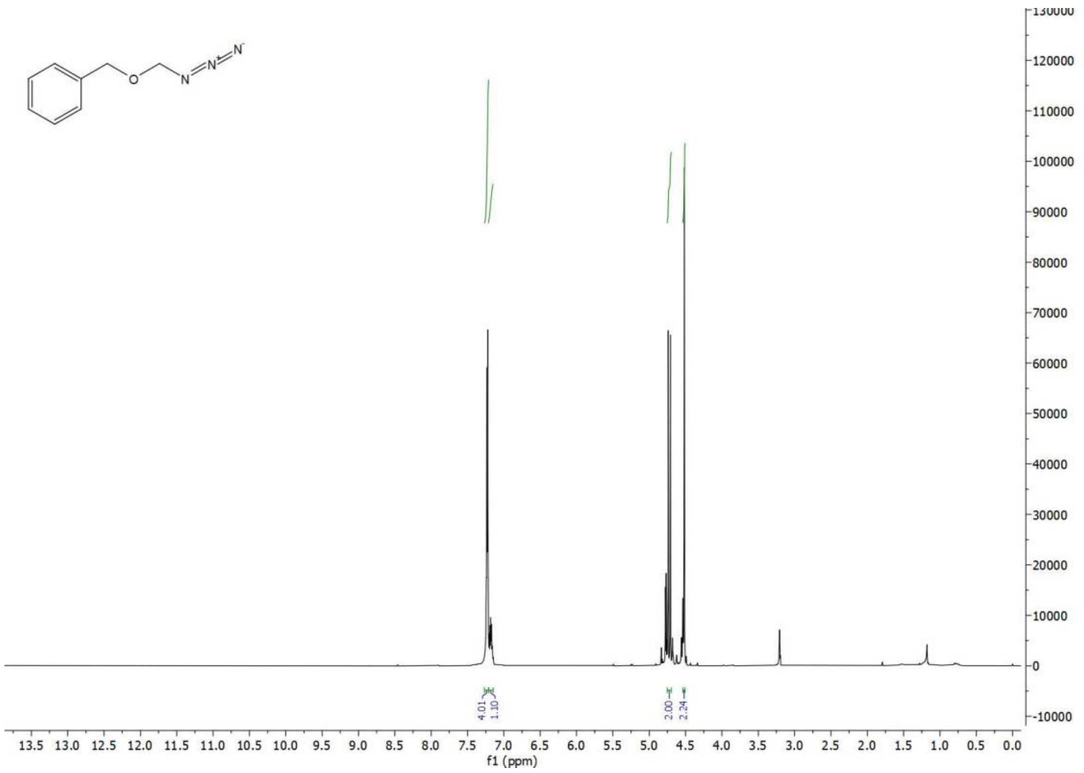

Figure S3. Intermediate 1a ^{1}H NMR (400 MHz, CD_3OD)

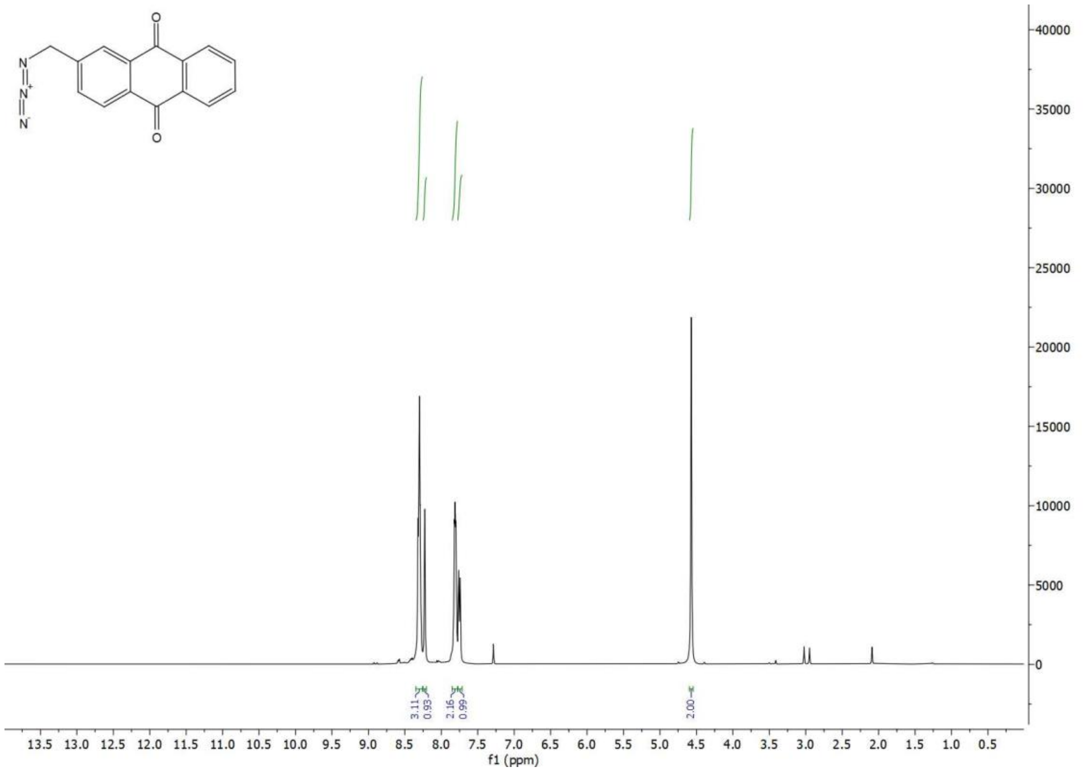

Figure S4. Intermediate 1b ^{1}H NMR (400 MHz, $CDCl_3$)

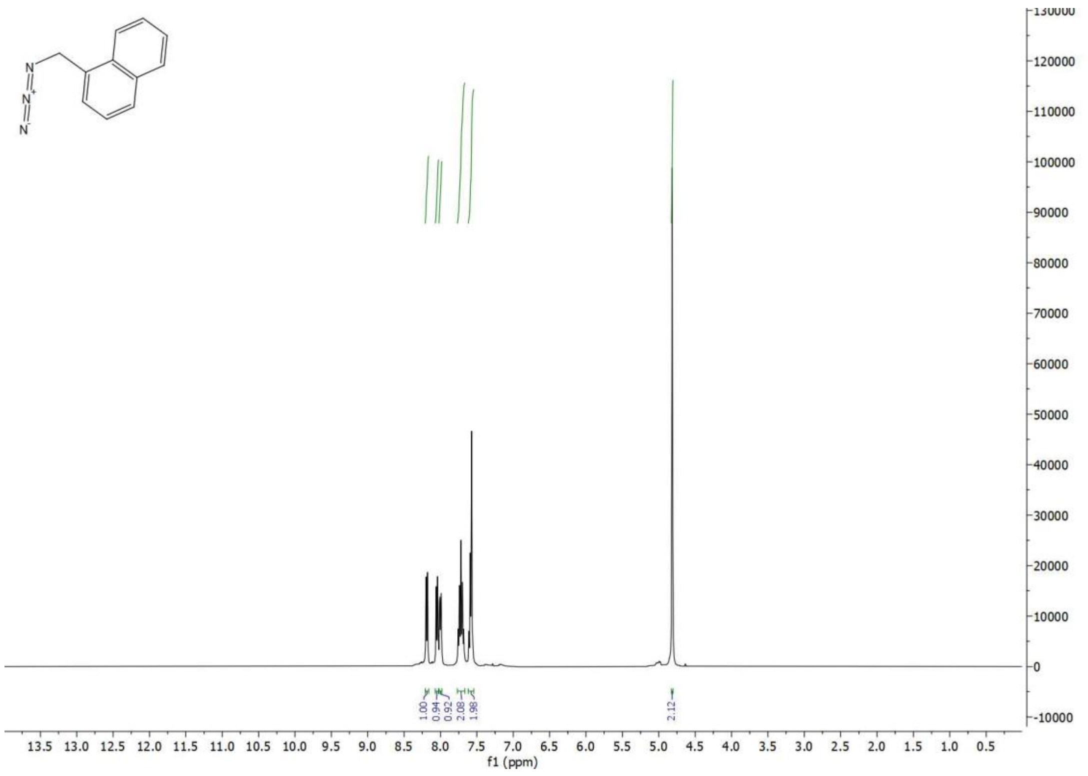

Figure S5. Intermediate 1c ^{1}H NMR (400 MHz, $CDCl_3$)

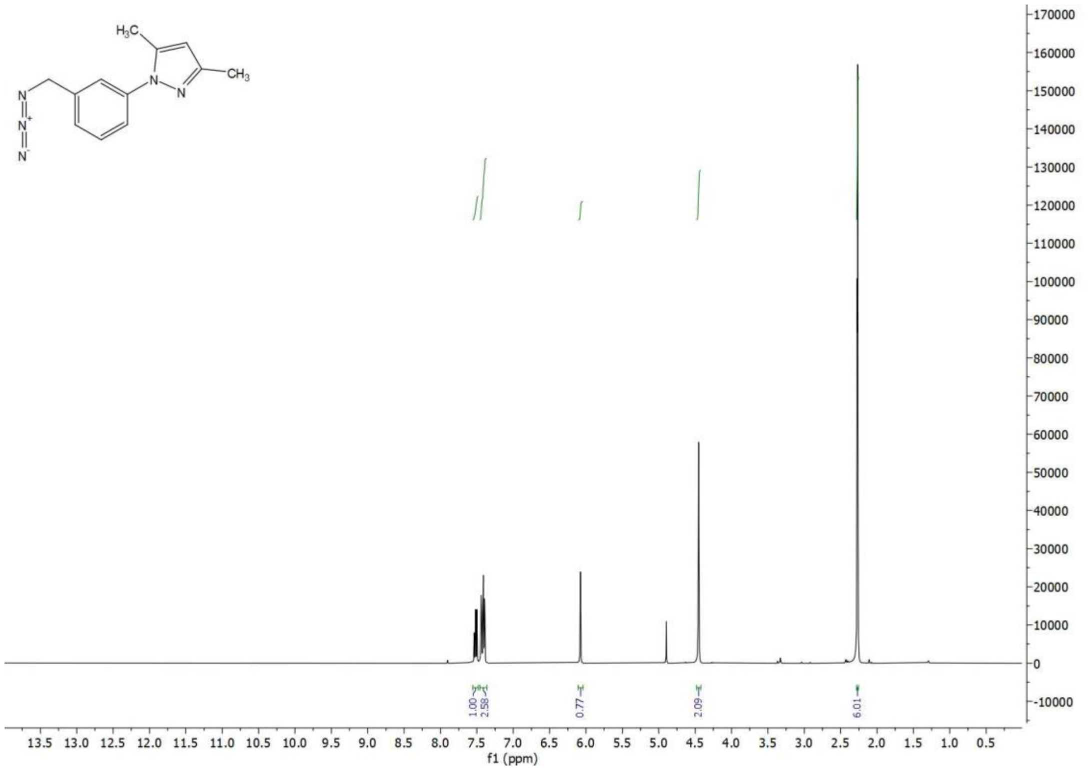

Figure S6. Intermediate 1d ^{1}H NMR (400 MHz, CD_3OD)

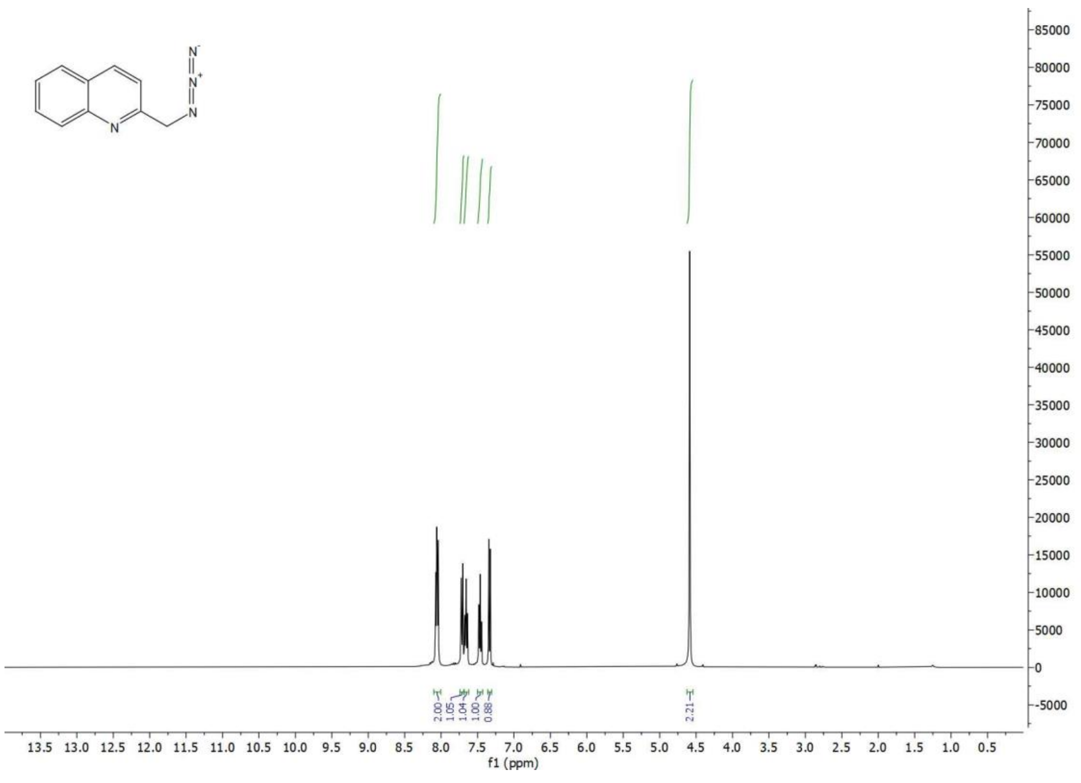

Figure S7. Intermediate 1e ^{1}H NMR (400 MHz, $CDCl_3$)

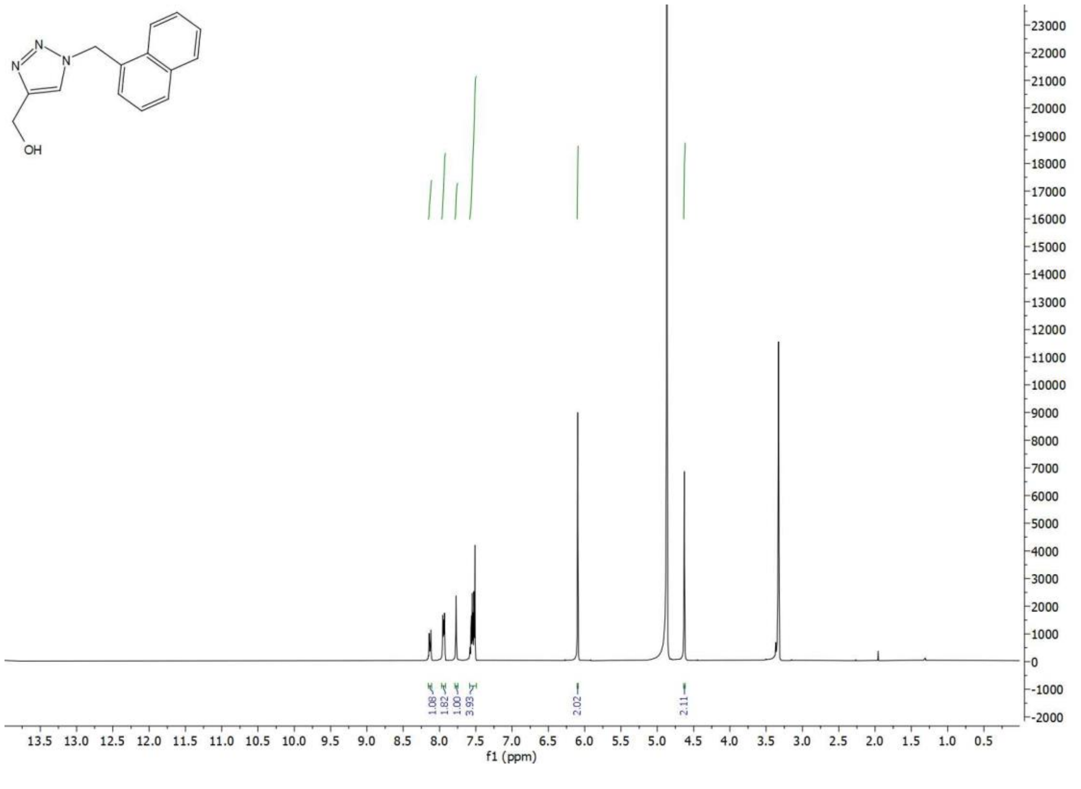

Figure S8. Intermediate 1c' ^{1}H NMR (400 MHz, CD_3OD)

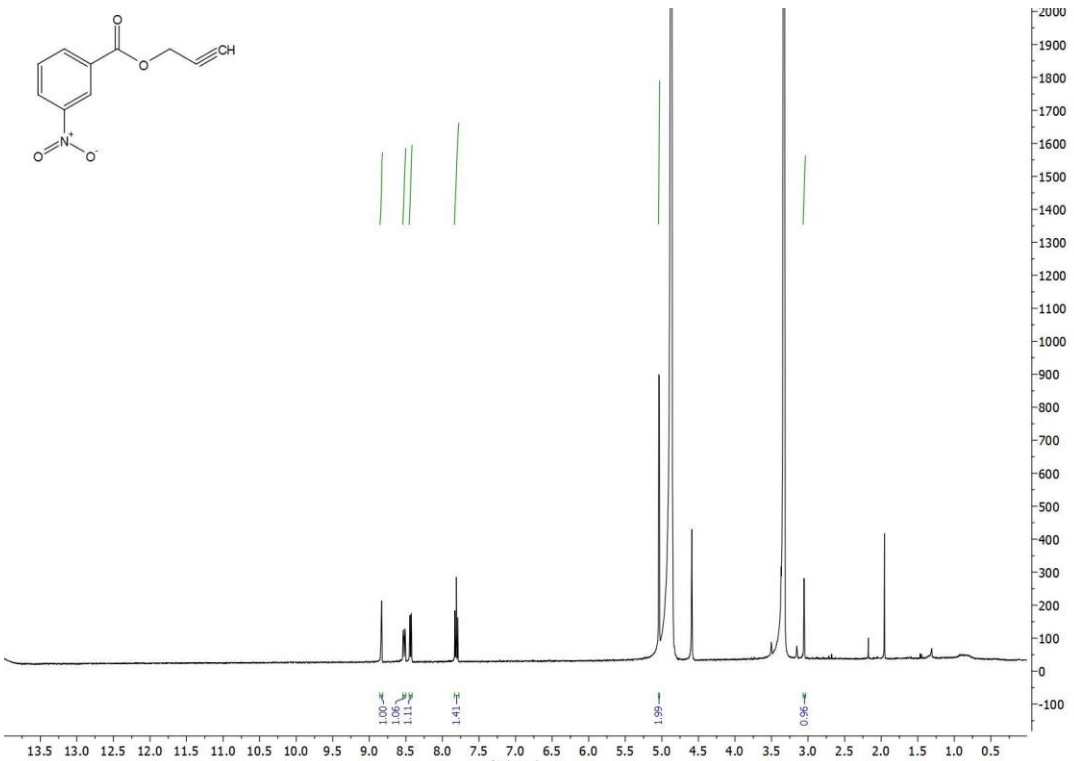

Figure S9. Intermediate 2f ^{1}H NMR (400 MHz, CD_3OD)

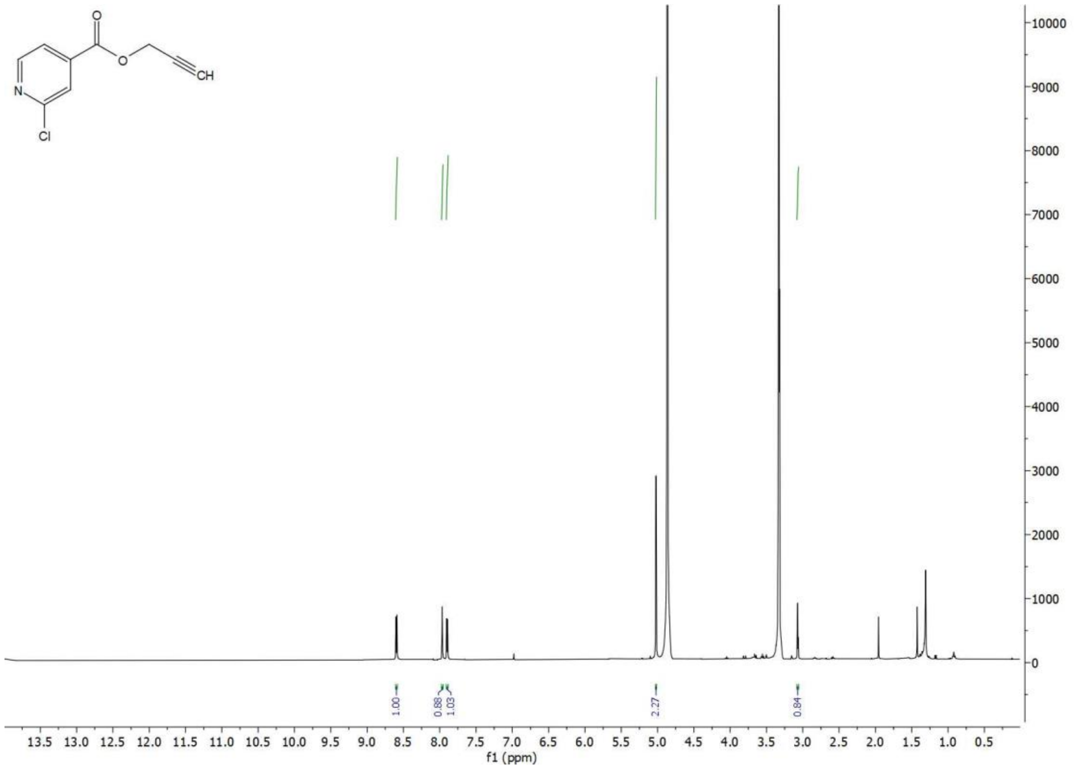

Figure S10. Intermediate 2g ^{1}H NMR (400 MHz, CD_3OD)

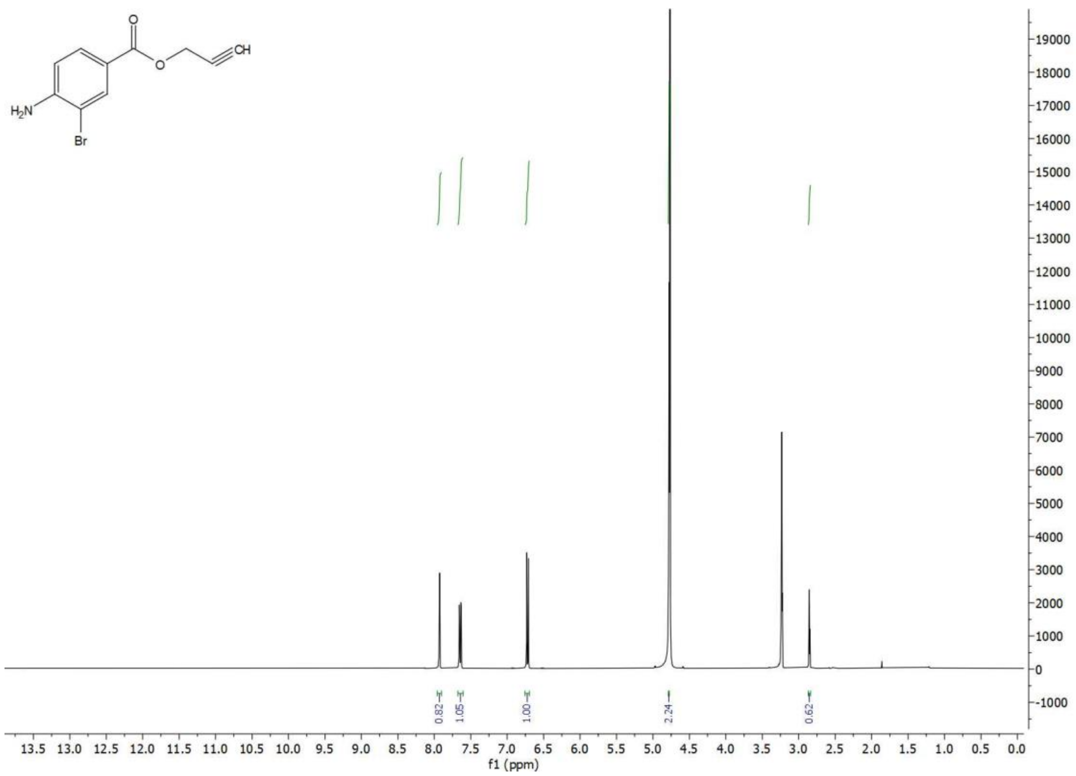

Figure S11. Intermediate 2h ^{1}H NMR (400 MHz, CD_3OD)

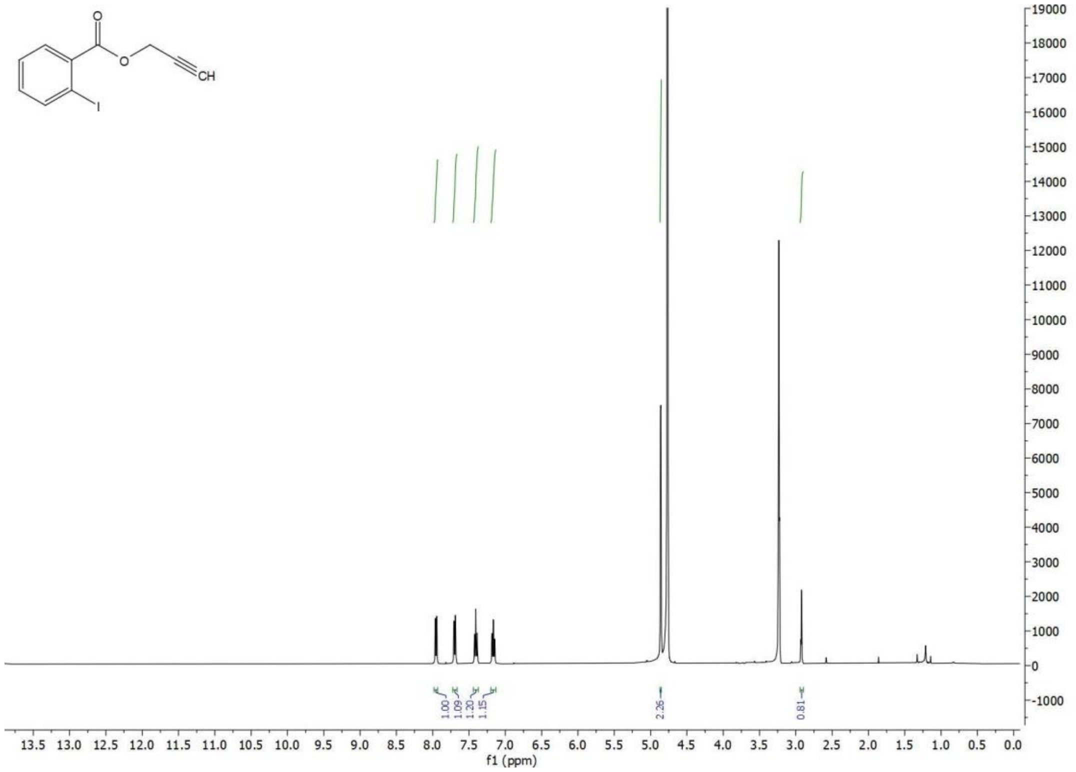

Figure S12. Intermediate 2i ^{1}H NMR (400 MHz, CD_3OD)

FINAL PRODUCTS

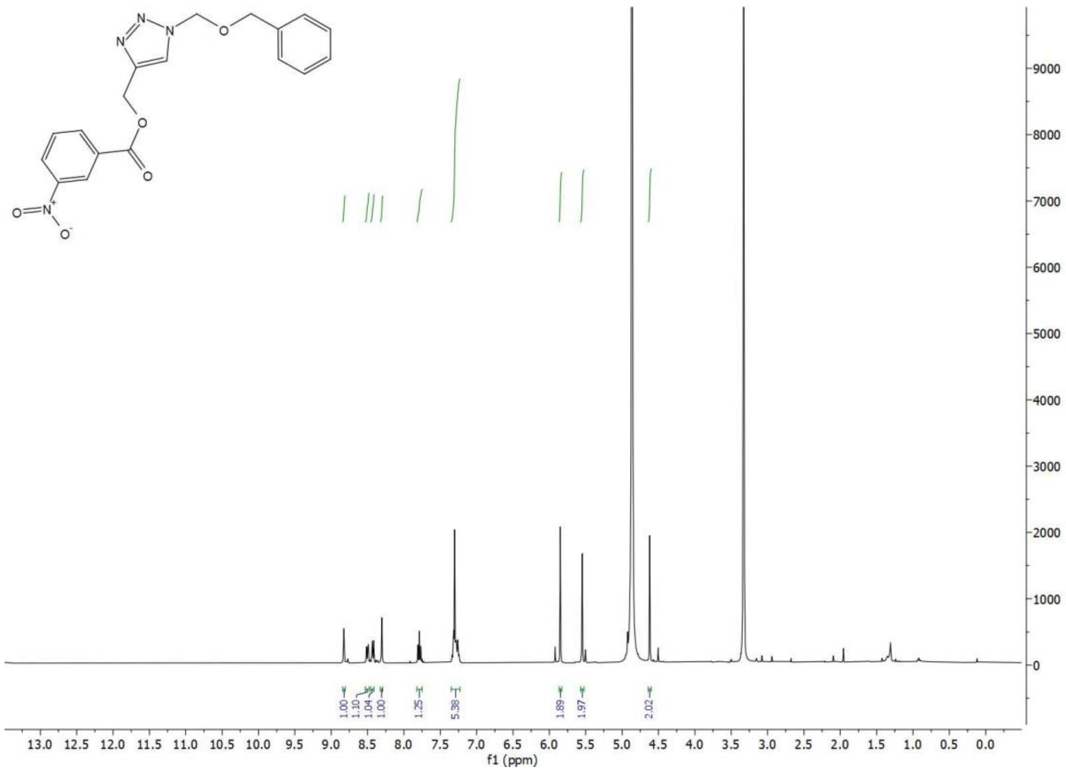

Figure S13. Compound 1 ^{1}H NMR (400 MHz, CD_3OD)

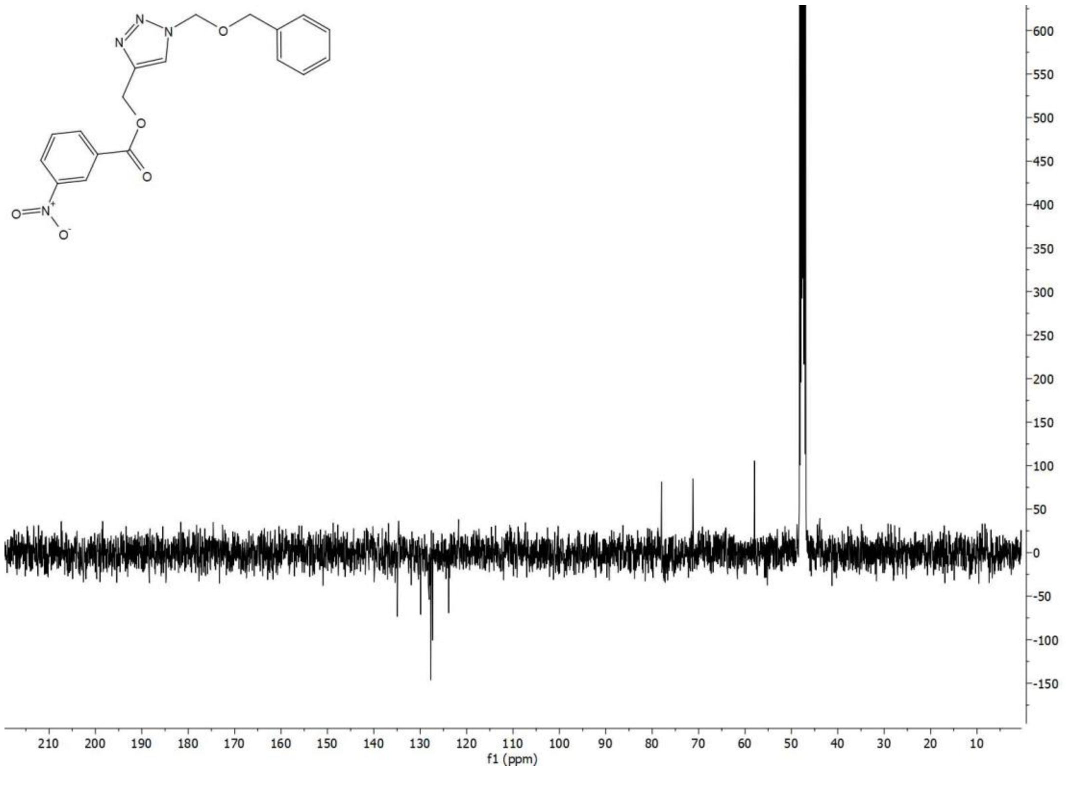

Figure S14. Compound 1 ^{13}C NMR (101 MHz, CD_3OD)

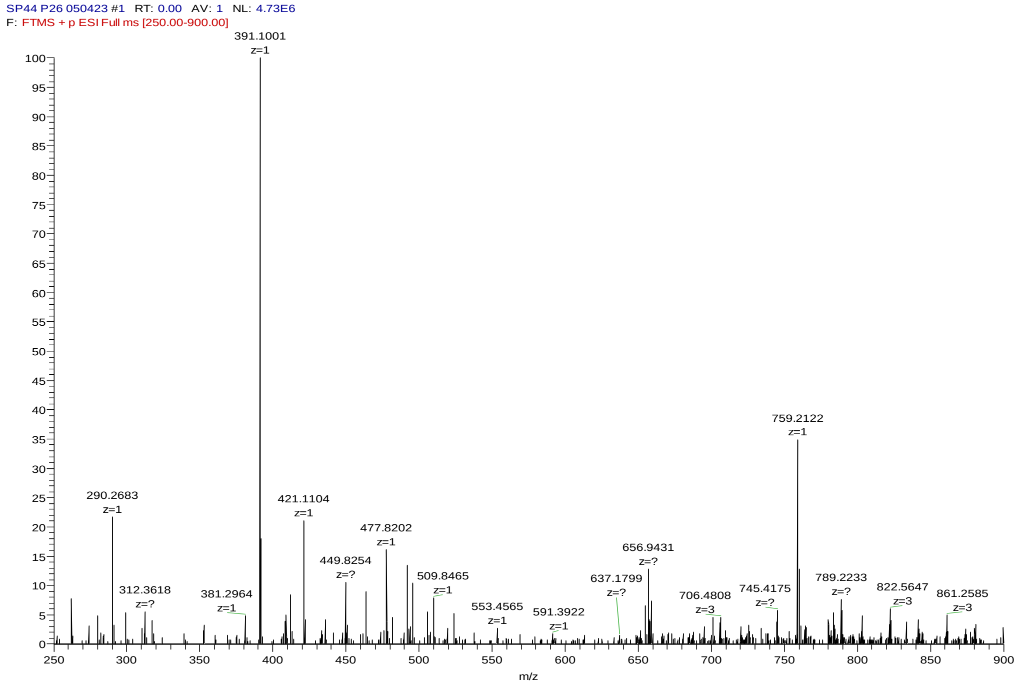

Figure S15. Compound 1 ESI-MS spectrum

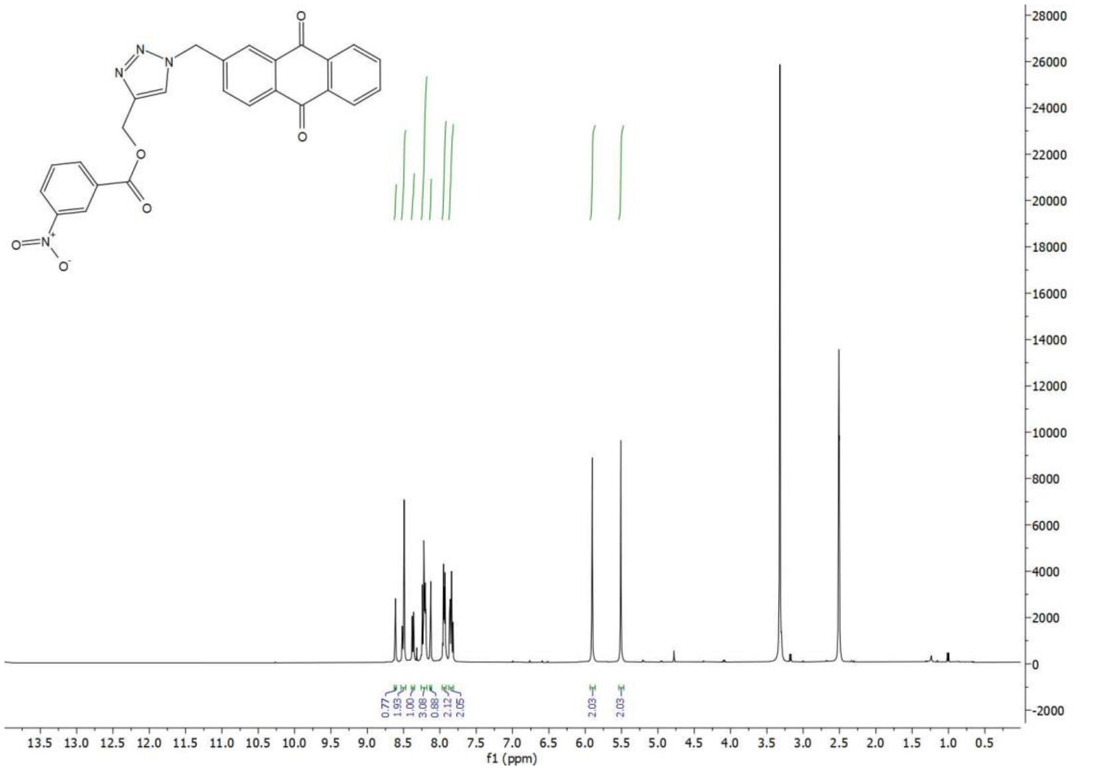

Figure S16. Compound 2 ^{1}H NMR (400 MHz, $(CD_3)_2SO$)

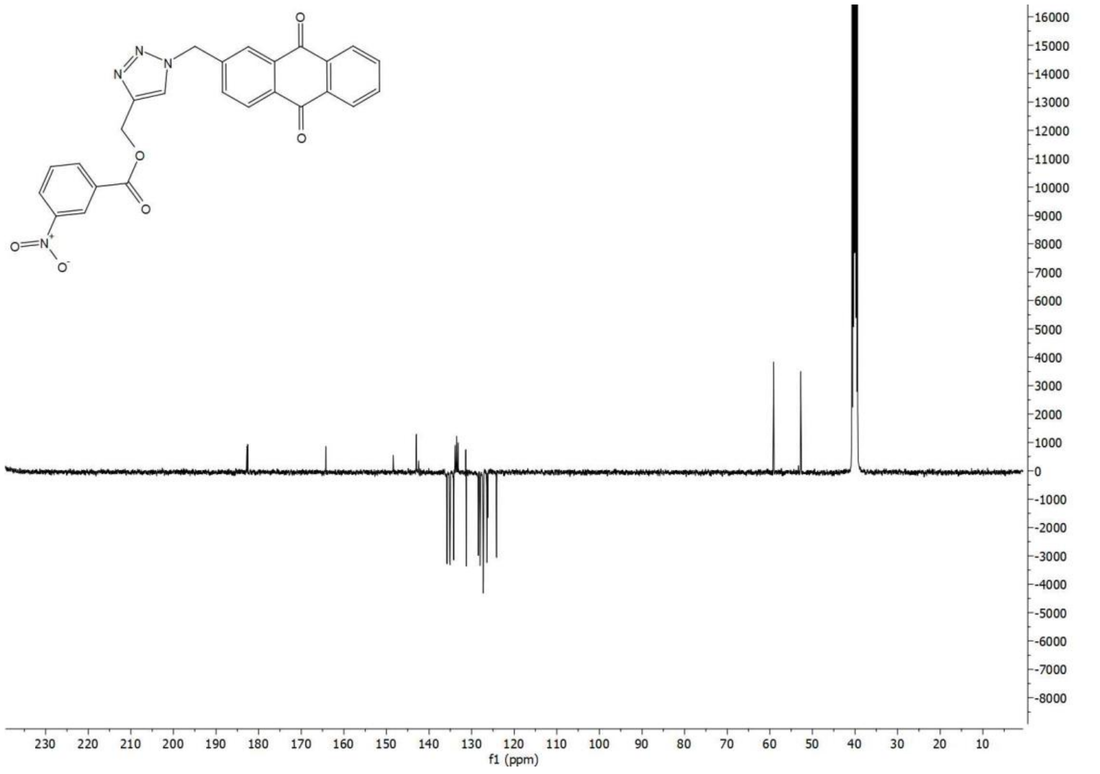

Figure S17. Compound 2 ^{13}C NMR (101 MHz, $(CD_3)_2SO$)

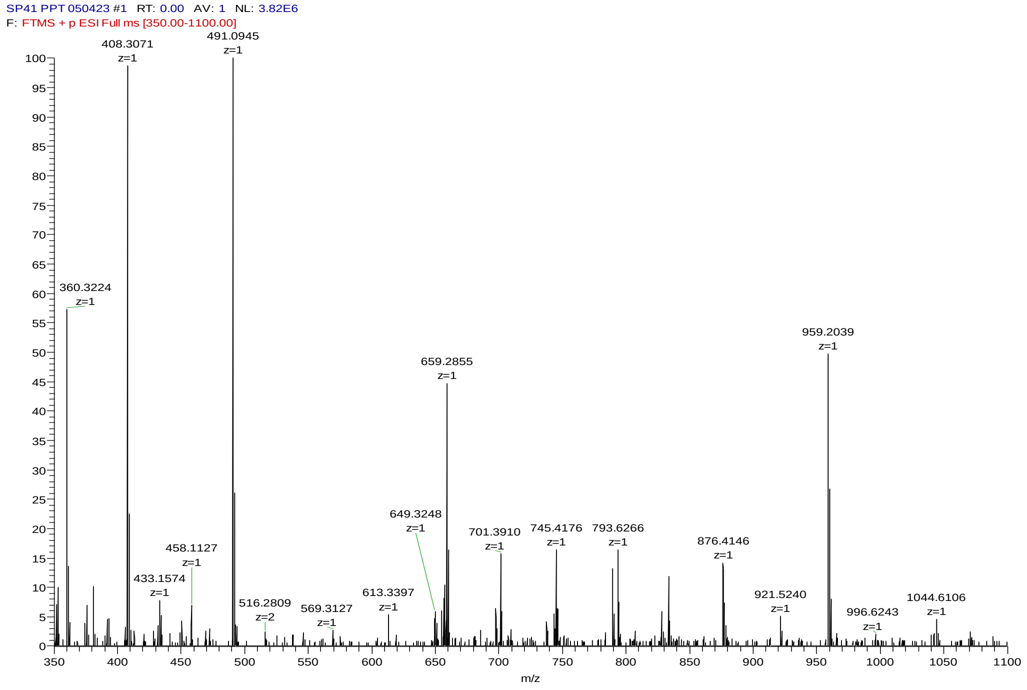

Figure S18. Compound 2 ESI-MS spectrum

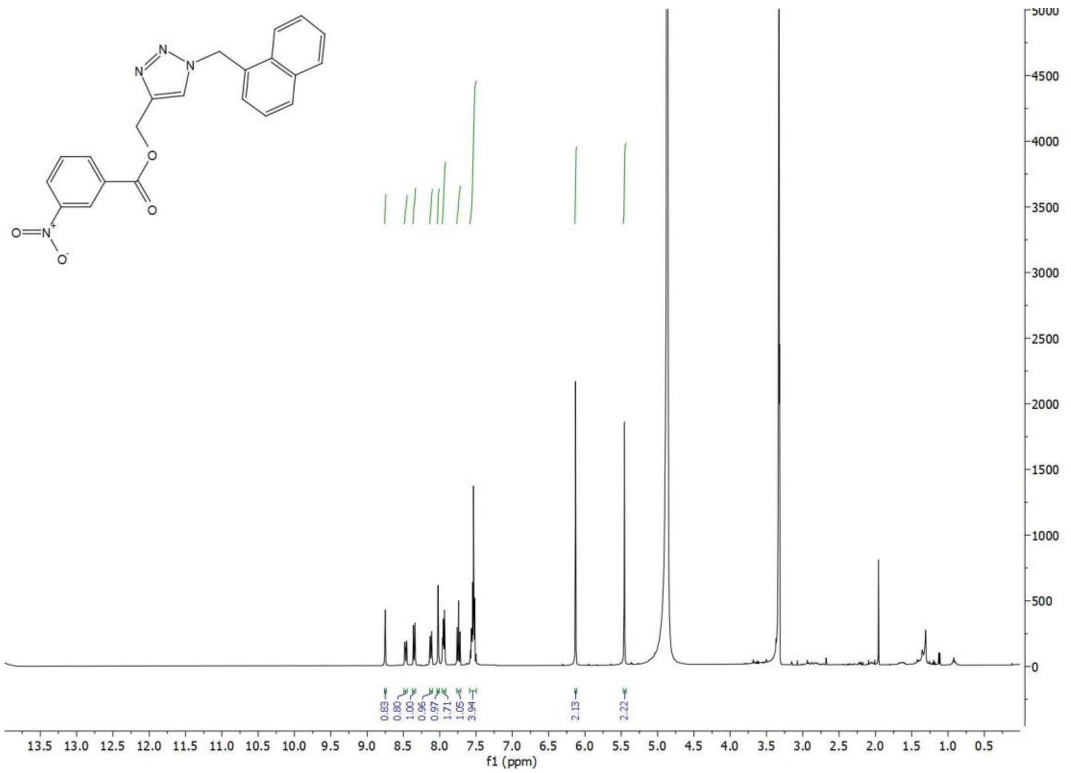

Figure S19. Compound 3 ^{1}H NMR (400 MHz, CD_3OD)

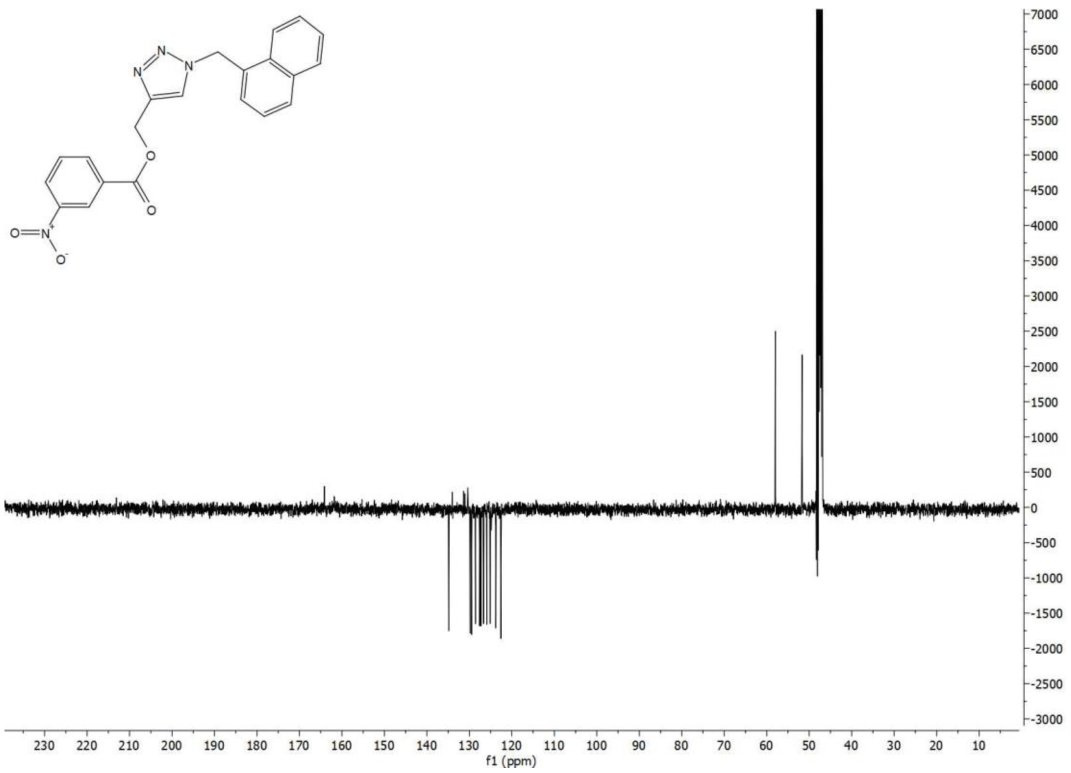

Figure S20. Compound 3 ^{13}C NMR (101 MHz, CD_3OD)

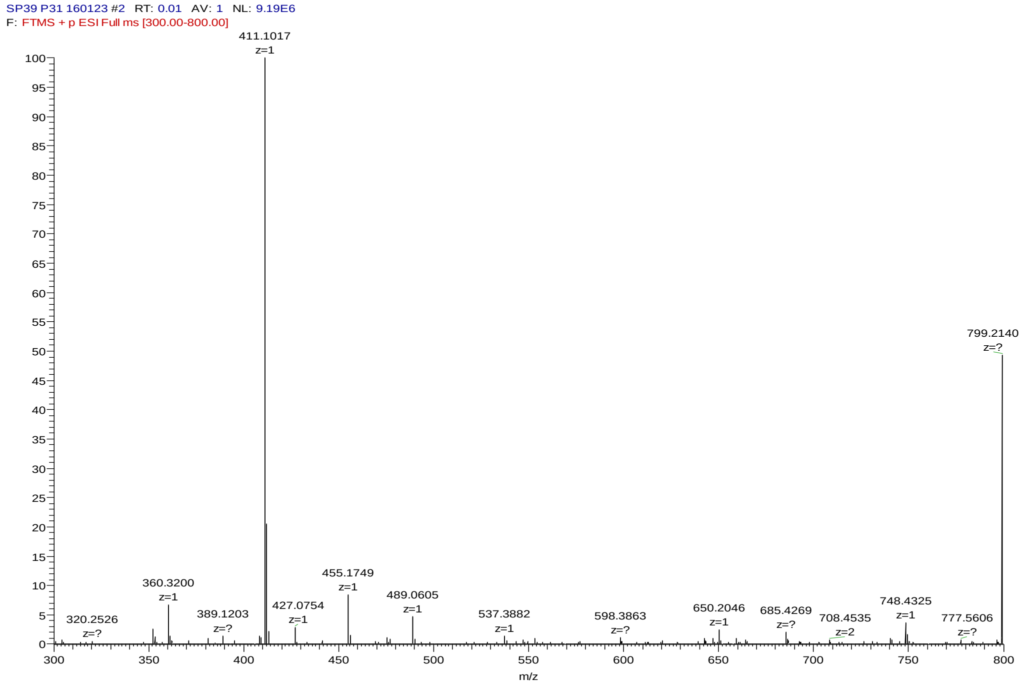

Figure S21. Compound 3 ESI-MS spectrum

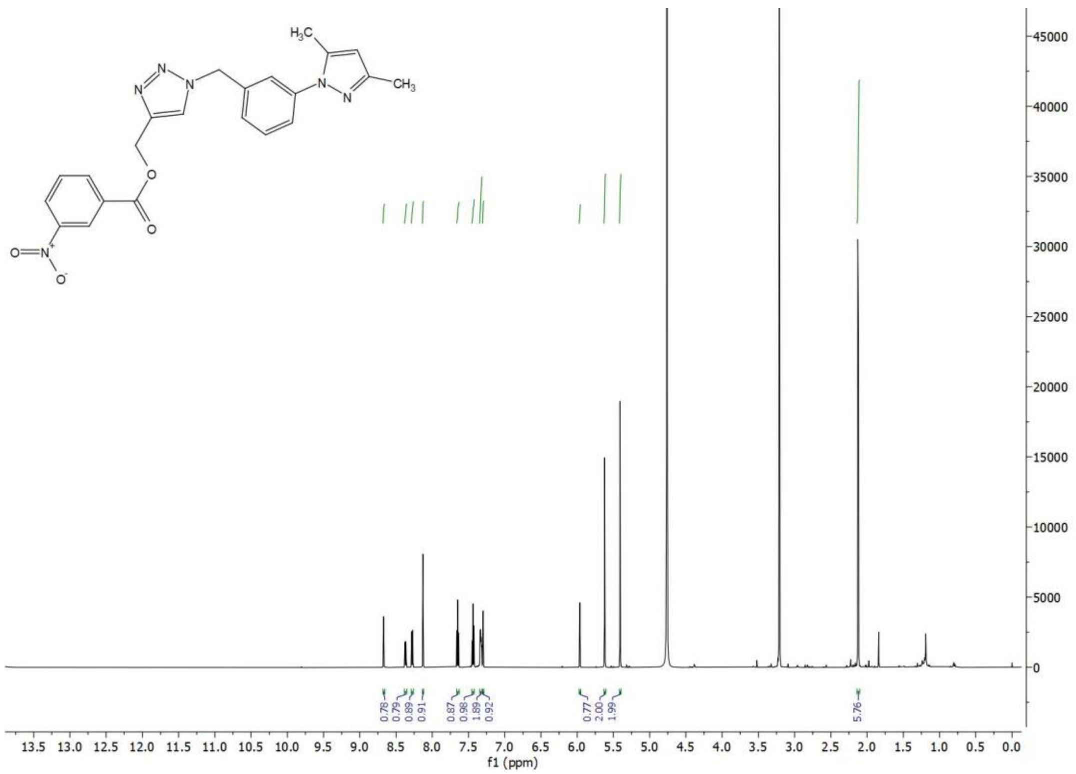

Figure S22. Compound 4 ^{1}H NMR (600 MHz, CD_3OD)

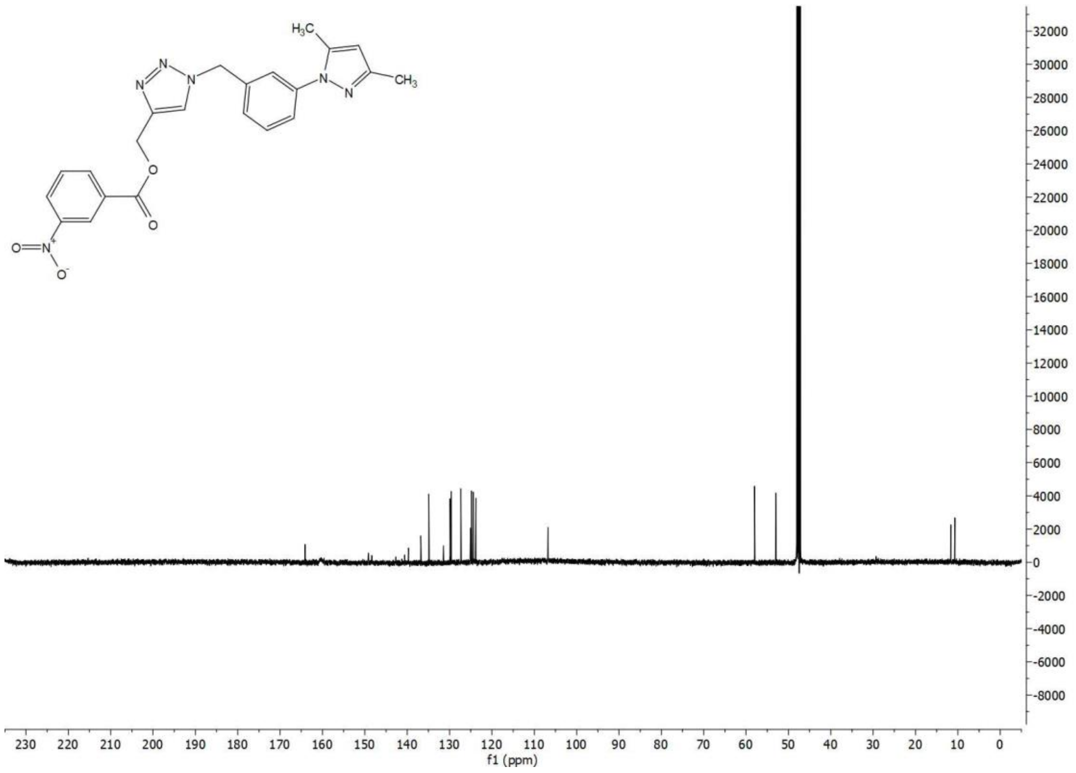

Figure S23. Compound 4 ^{13}C NMR (151 MHz, CD_3OD)

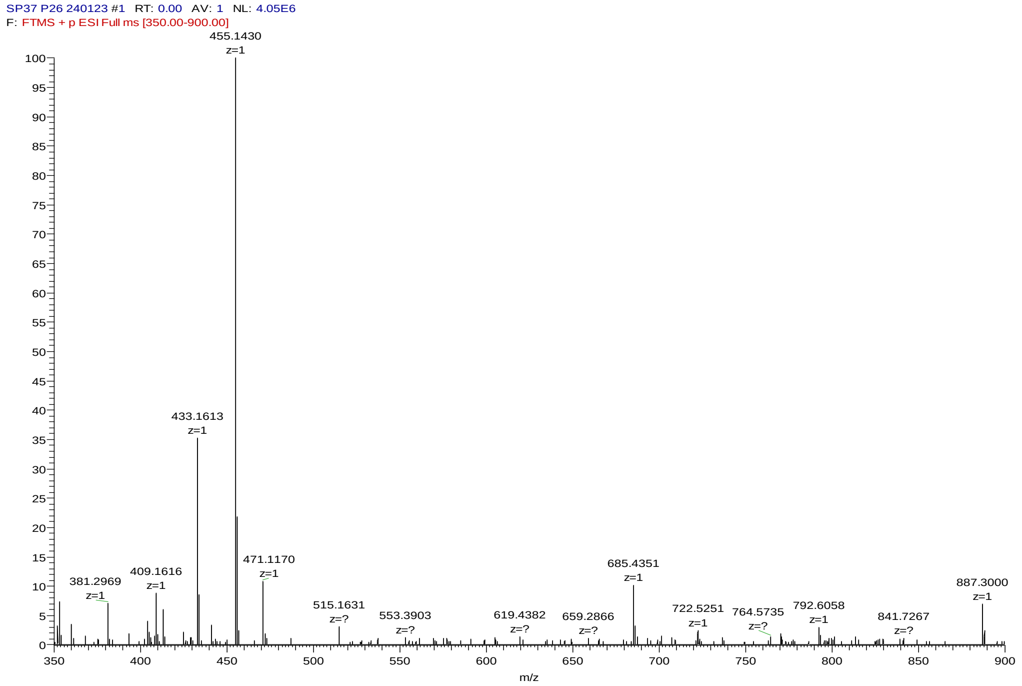

Figure S24. Compound 4 ESI-MS spectrum

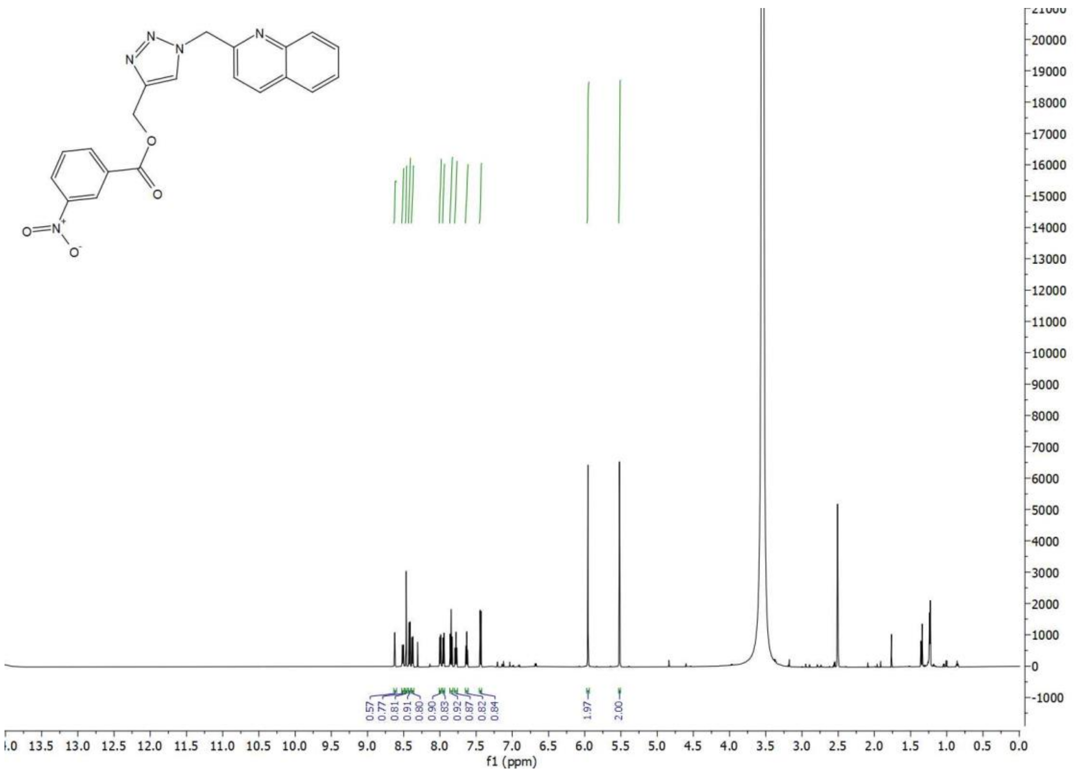

Figure S25. Compound 5 ^{1}H NMR (400 MHz, $(CD_3)_2SO$)

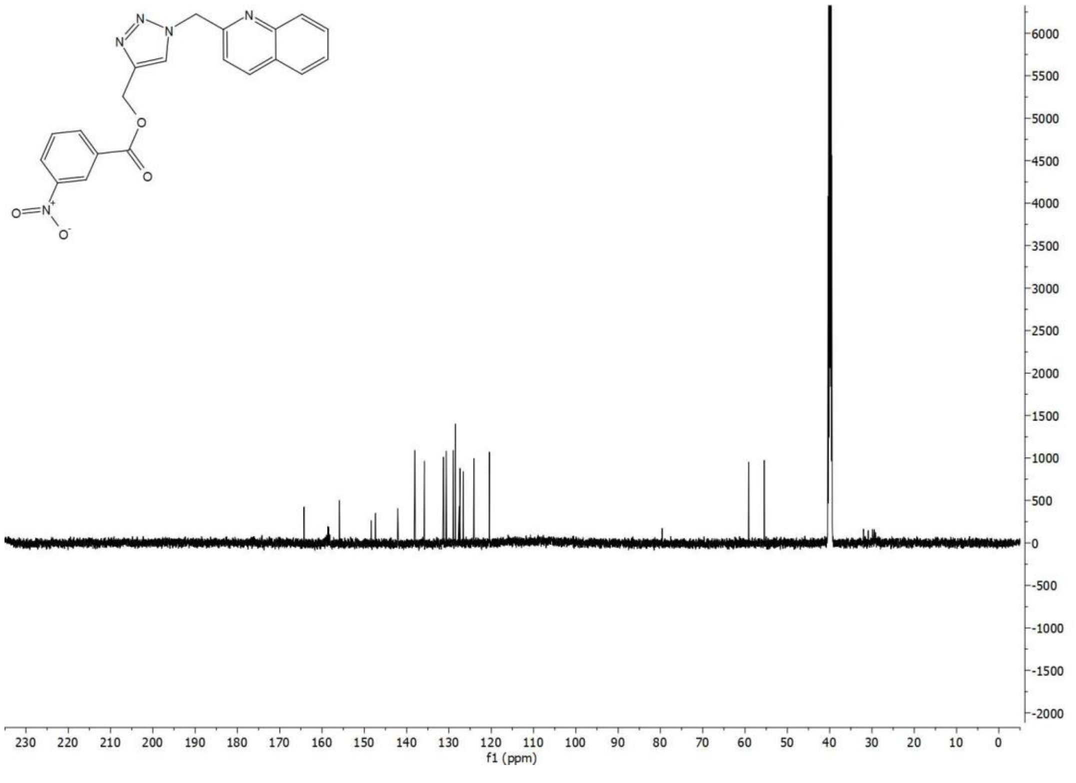

Figure S26. Compound 5 ^{13}C NMR (151 MHz, $(CD_3)_2SO$)

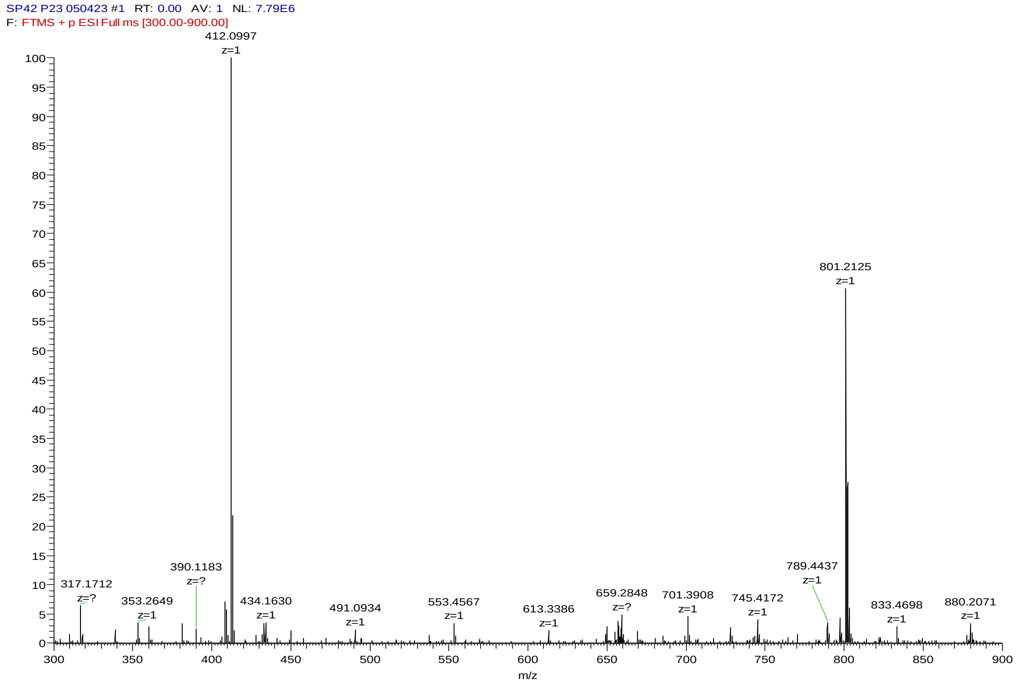

Figure S27. Compound 5 ESI-MS spectra

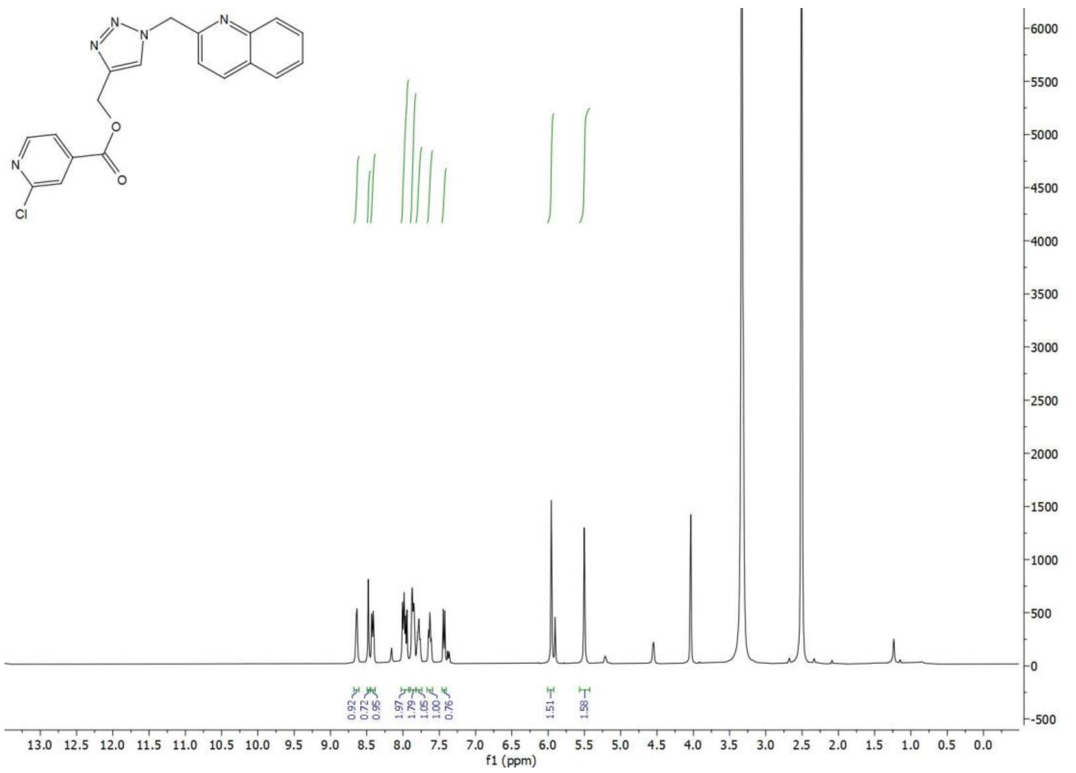

Figure S28. Compound 6 ^{1}H NMR (400 MHz, $(CD_3)_2SO$)

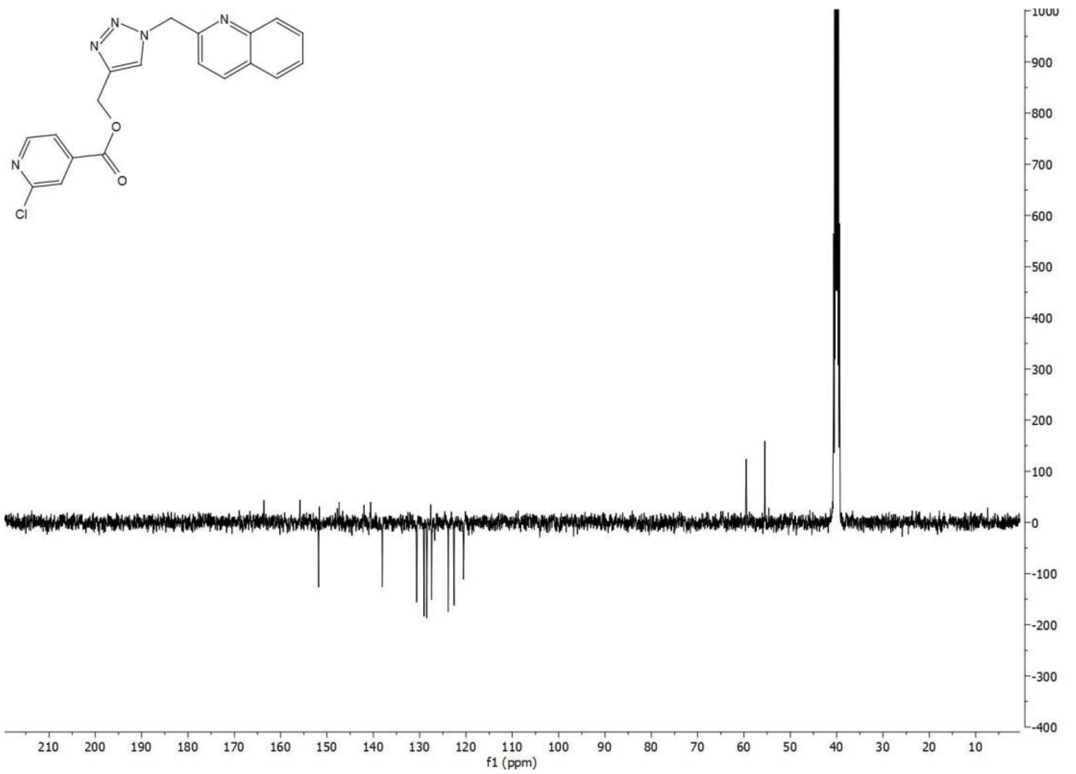

Figure S29. Compound 6 ^{13}C NMR (101 MHz, $(CD_3)_2SO$)

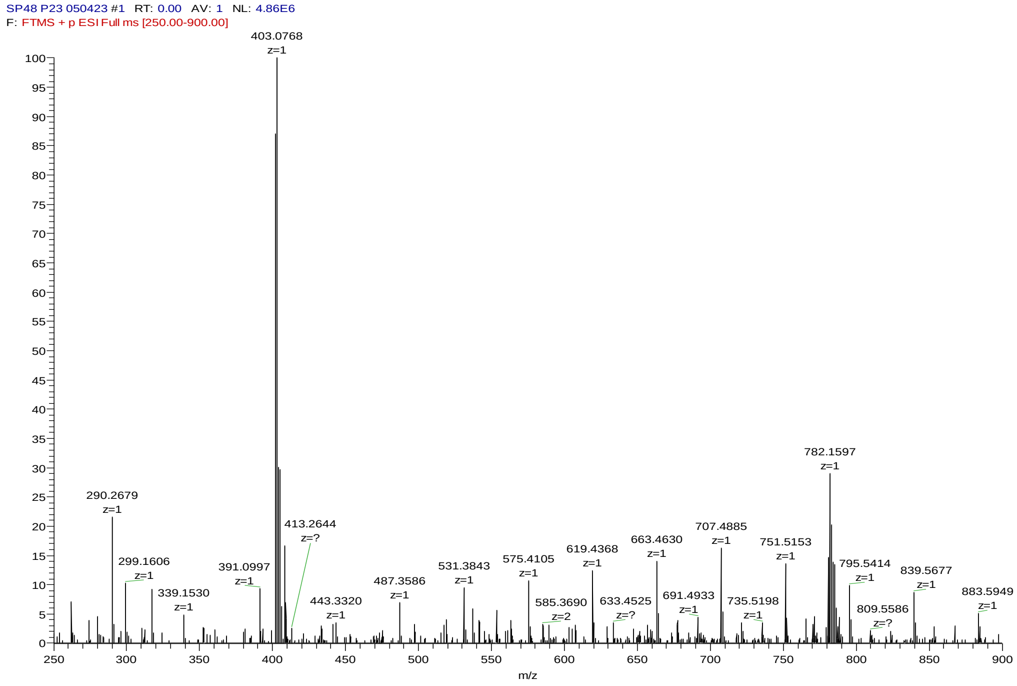

Figure S30. Compound 6 ESI-MS spectrum

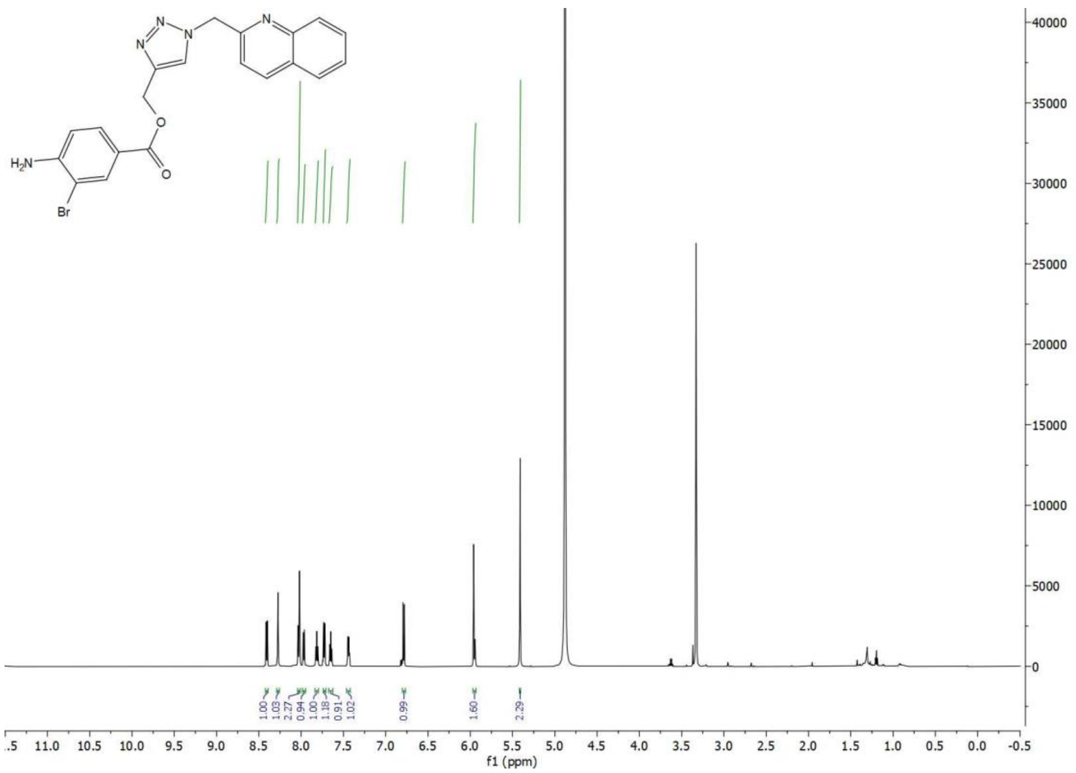

Figure S31. Compound 7 ^{1}H NMR (600 MHz, CD_3OD)

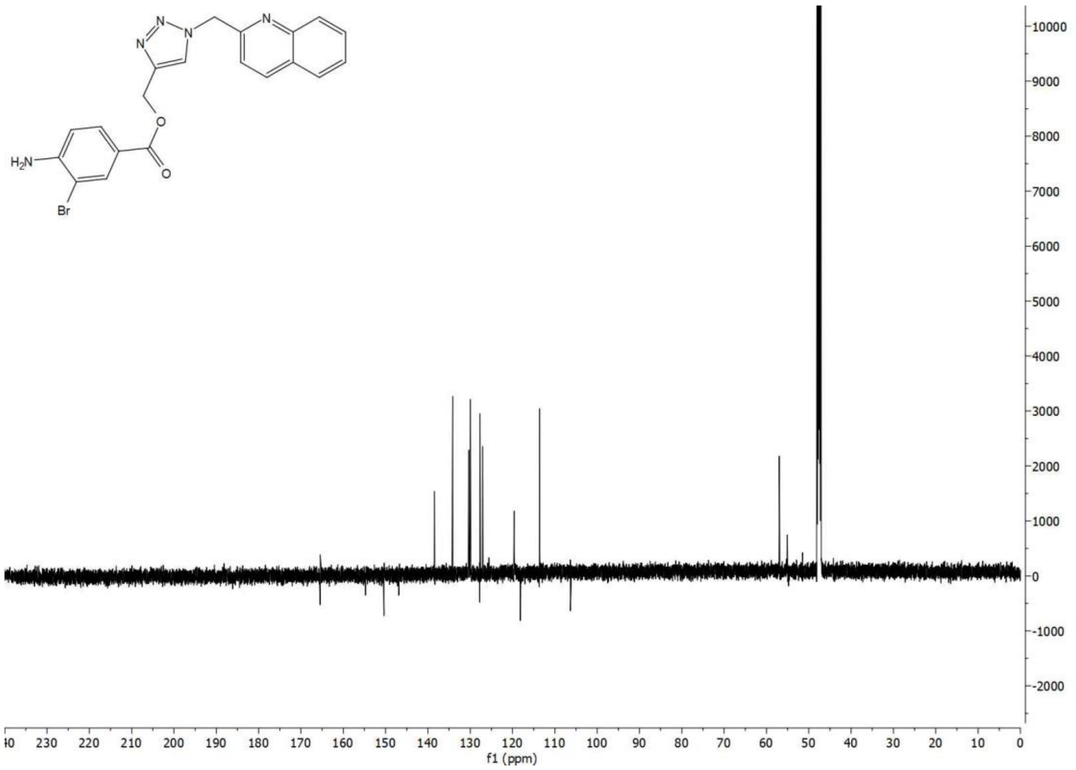

Figure S32. Compound 7 ^{13}C NMR (151 MHz, CD_3OD)

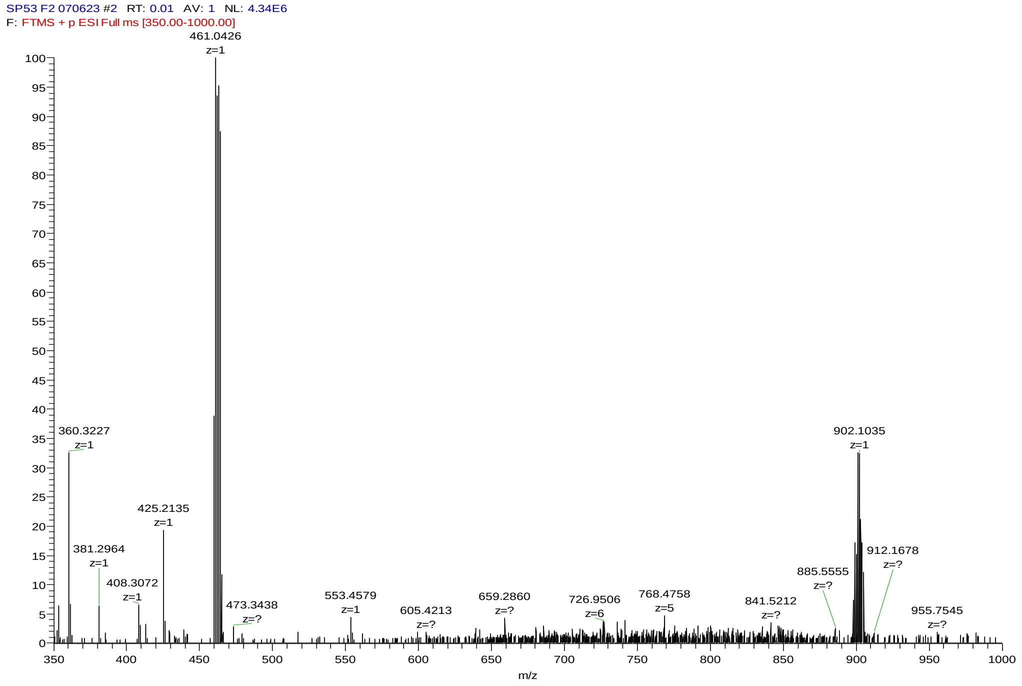

Figure S33. Compound 7 ESI-MS spectrum

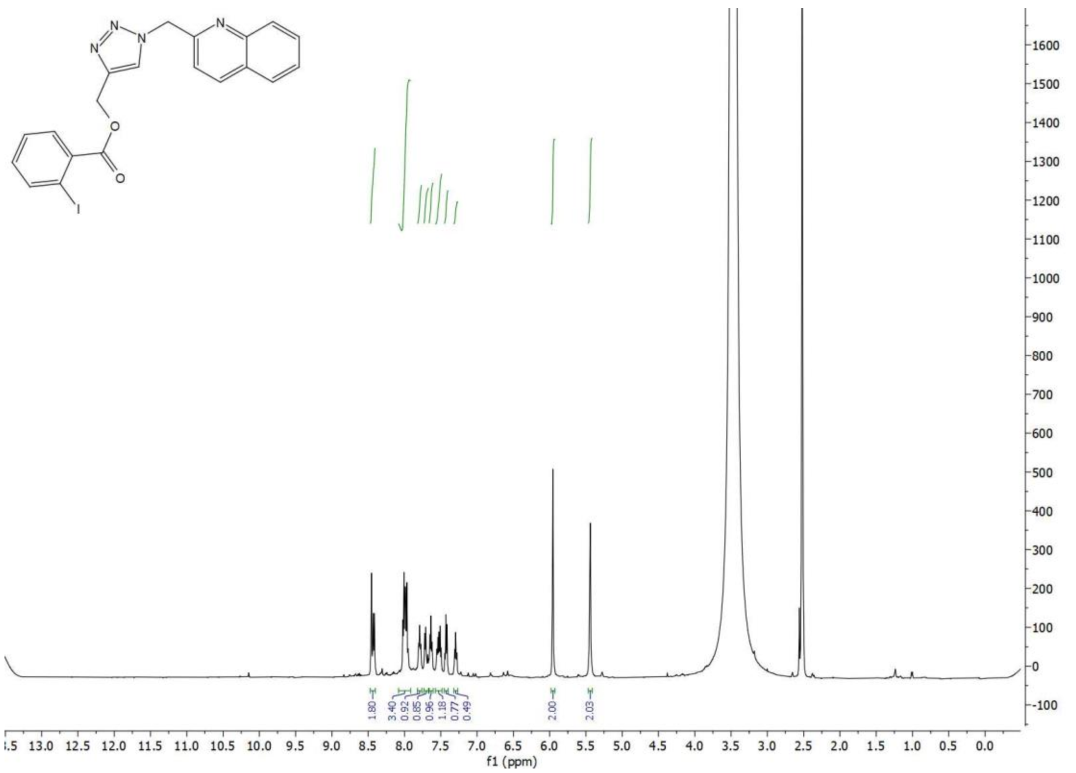

Figure S34. Compound 8 ^{1}H NMR (500 MHz, $(CD_3)_2SO$)

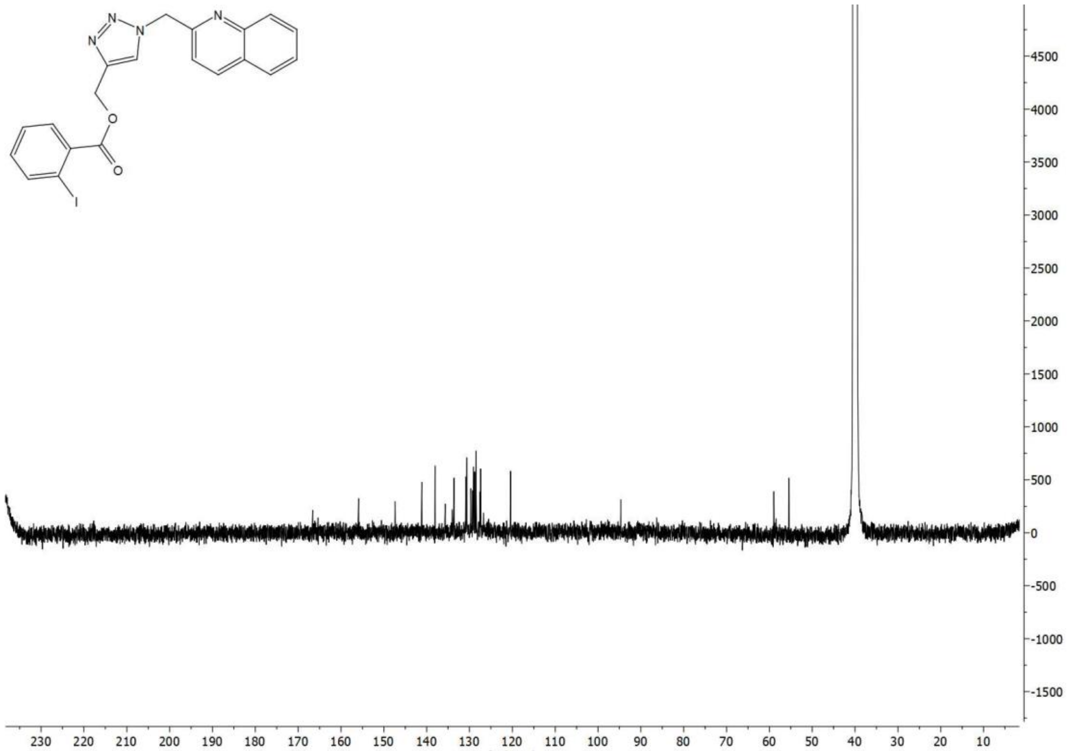

Figure S35. Compound 8 ^{13}C NMR (126 MHz, $(CD_3)_2SO$)

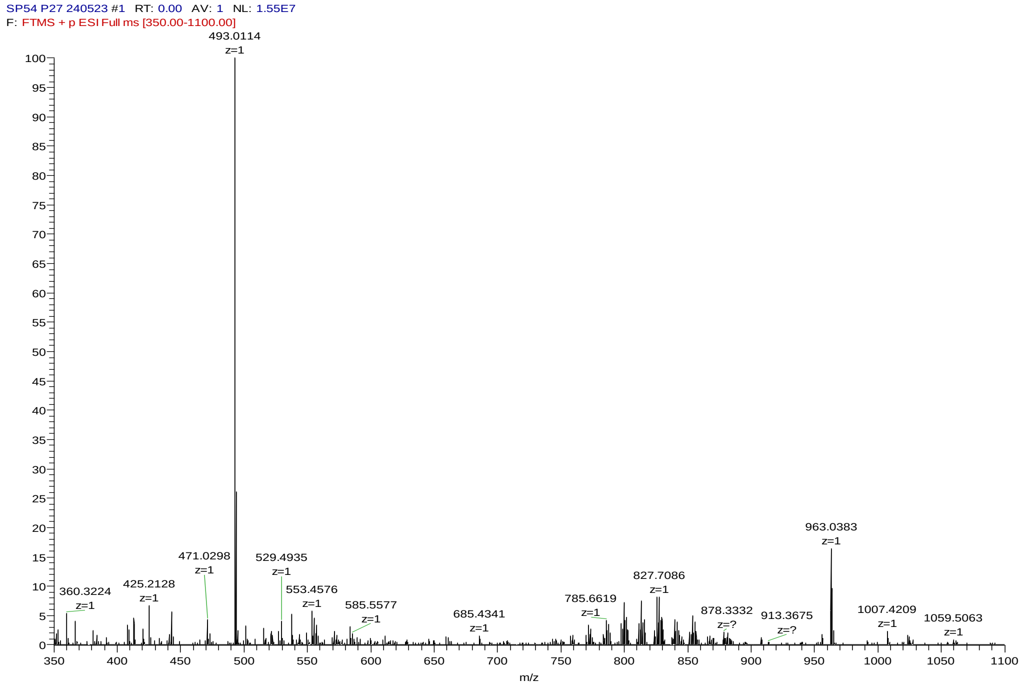

Figure S36. Compound 8 ESI-MS spectrum

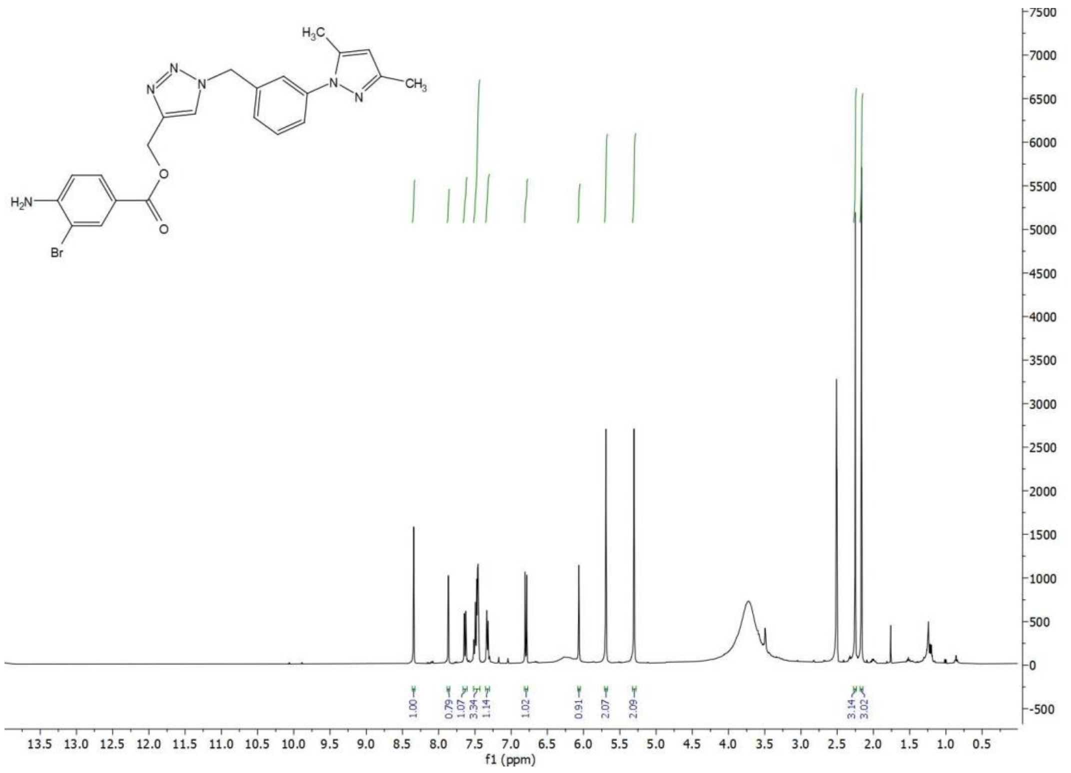

Figure S37. Compound 9 ^{1}H NMR (400 MHz, $(CD_3)_2SO$)

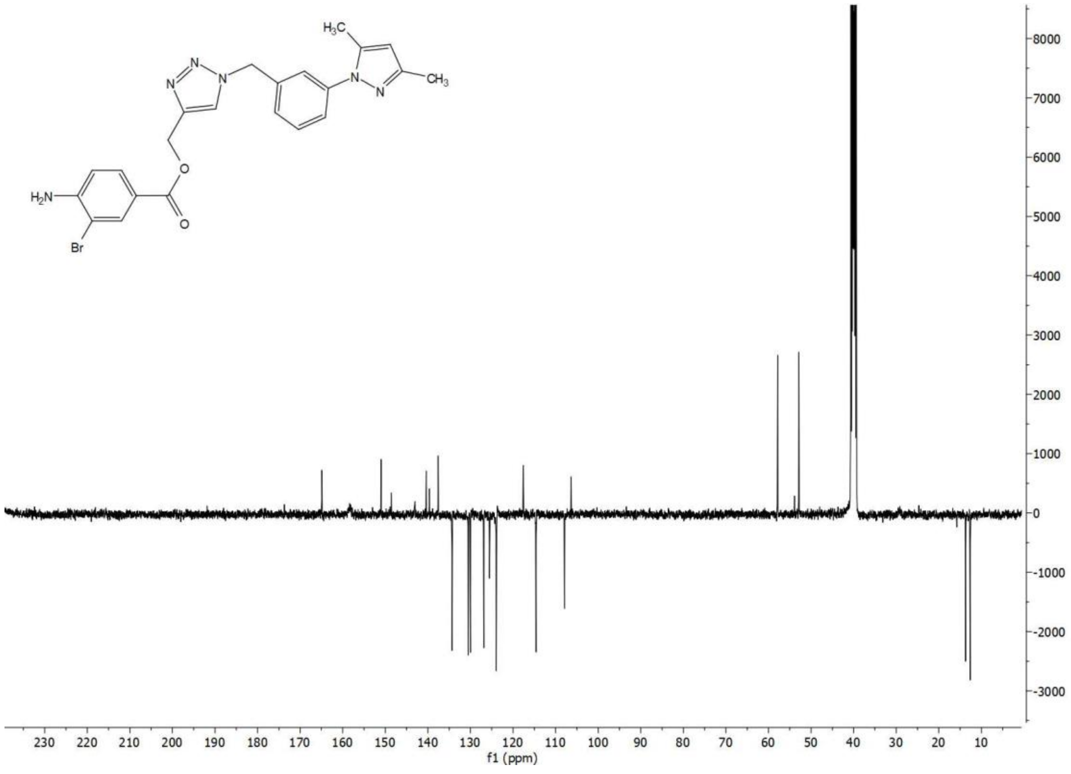

Figure S38. Compound 9 ^{13}C NMR (101 MHz, $(CD_3)_2SO$)

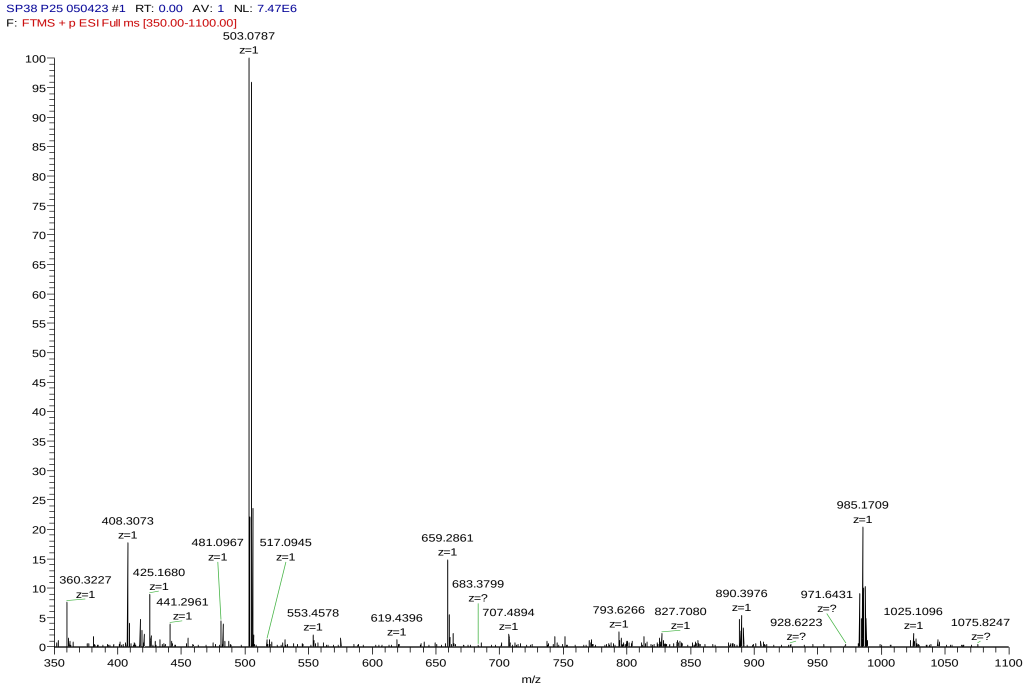

Figure S39. Compound 9 ESI-MS spectrum

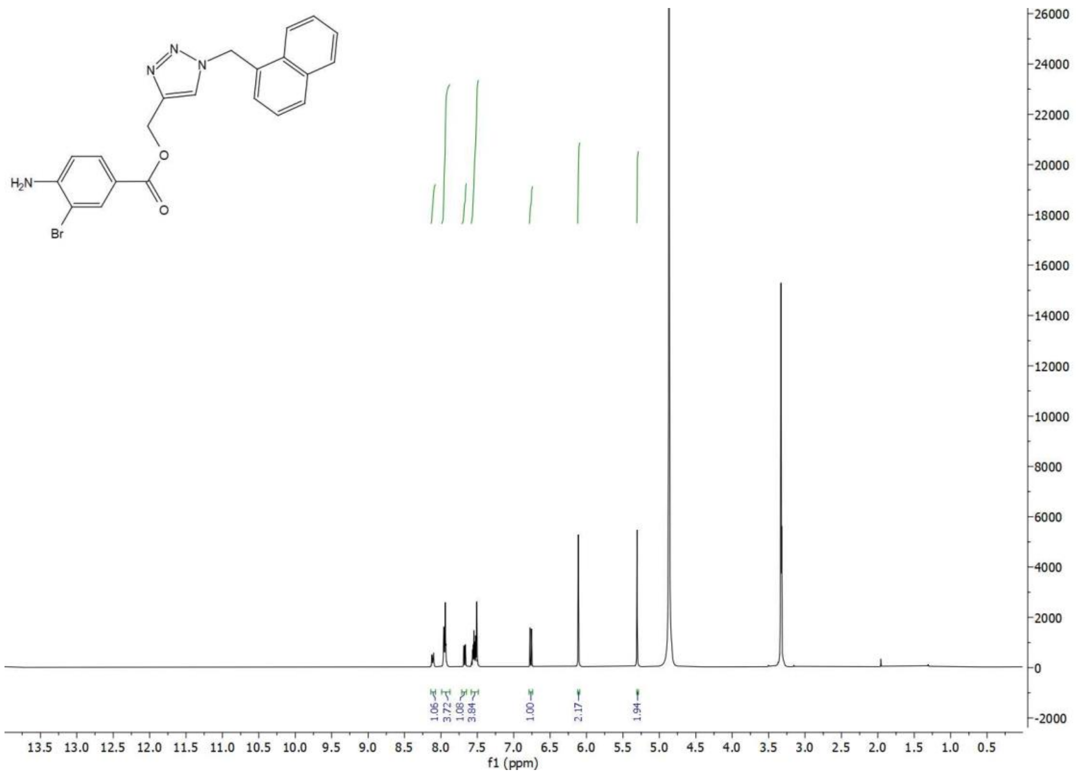

Figure S40. Compound 10 ^{1}H NMR (400 MHz, CD_3OD)

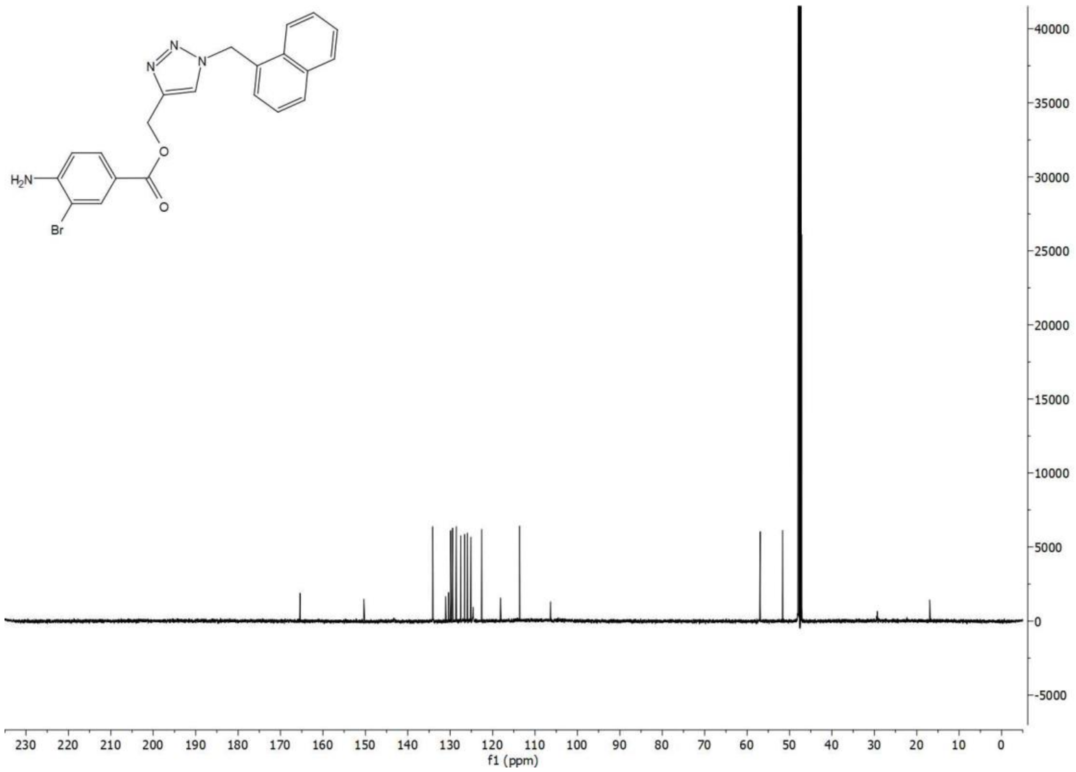

Figure S41. Compound 10 ^{13}C NMR (151 MHz, CD_3OD)

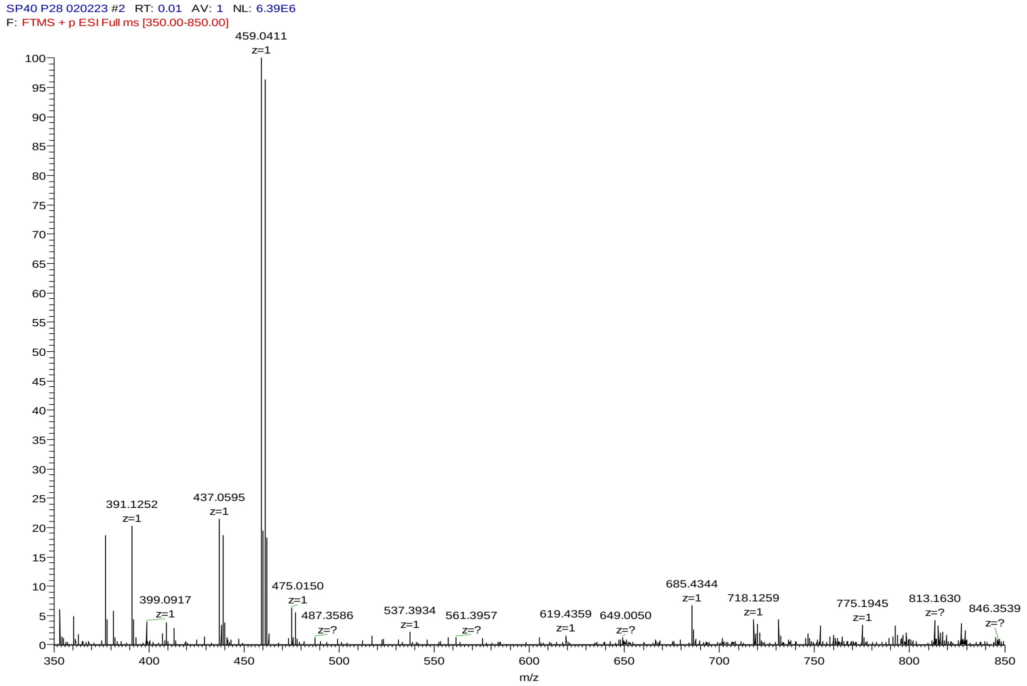

Figure S42. Compound 10 ESI-MS spectrum

SENSORGRAMS – BAG3 FULL

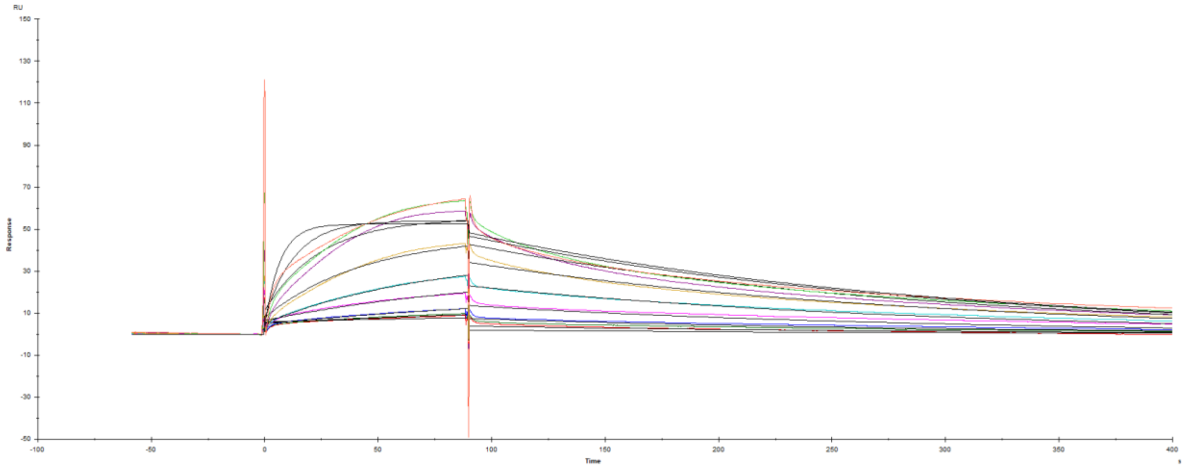

Figure S43. Sensorgram of compound 1 on BAG3 full

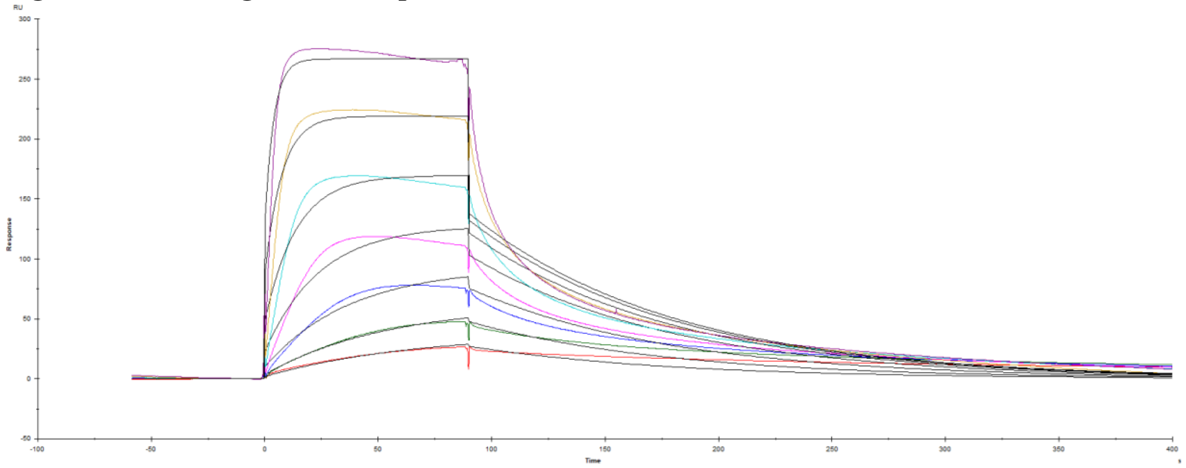

Figure S44. Sensorgram of compound 2 on BAG3 full

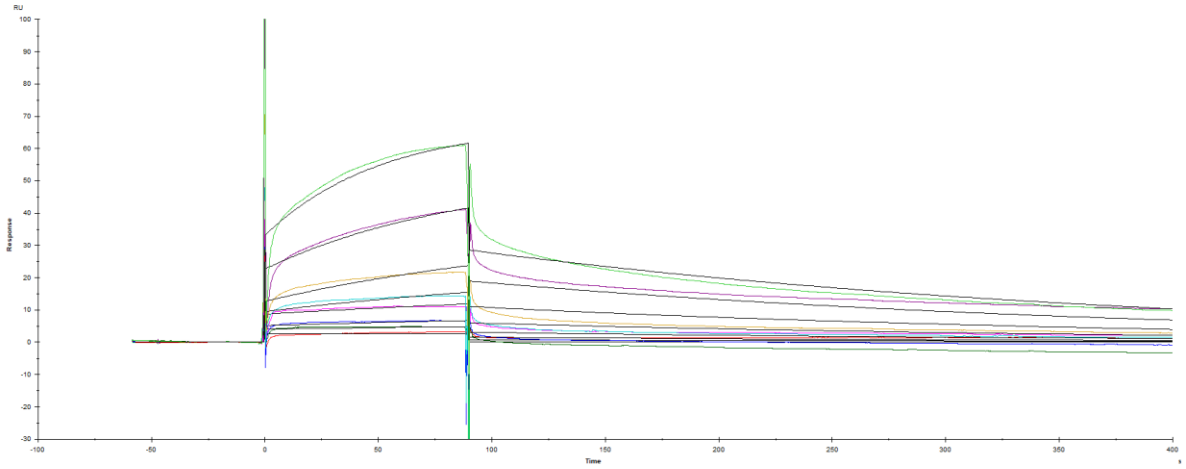

Figure S45. Sensorgram of compound 5 on BAG3 full

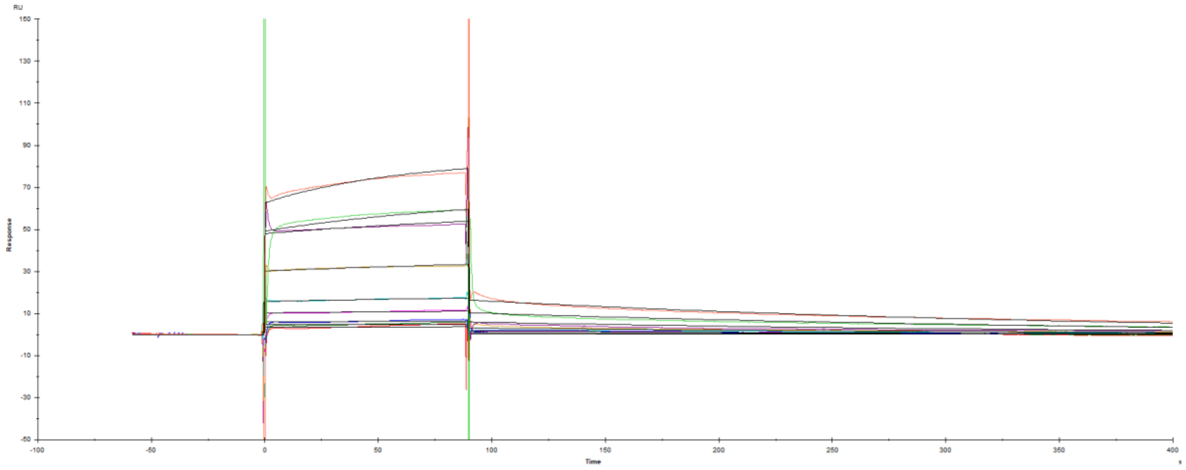

Figure S46. Sensorgram of compound 6 on BAG3 full

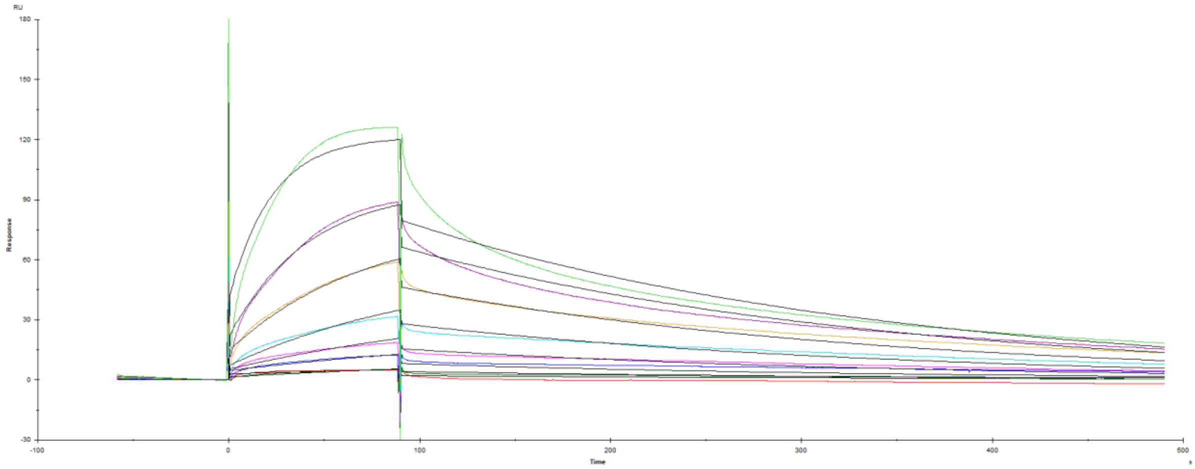

Figure S47. Sensorgram of compound 7 on BAG3 full

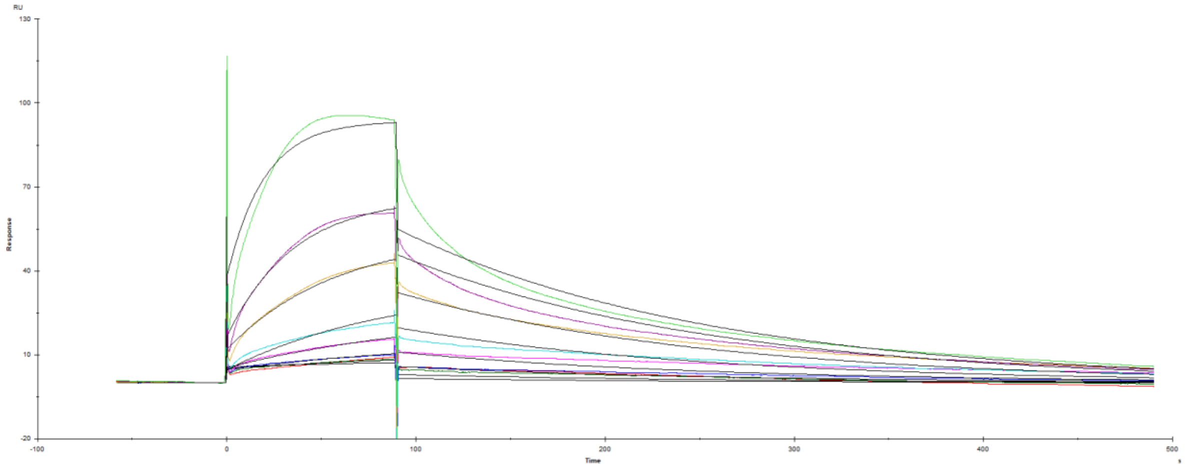

Figure S48. Sensorgram of compound 8 on BAG3 full

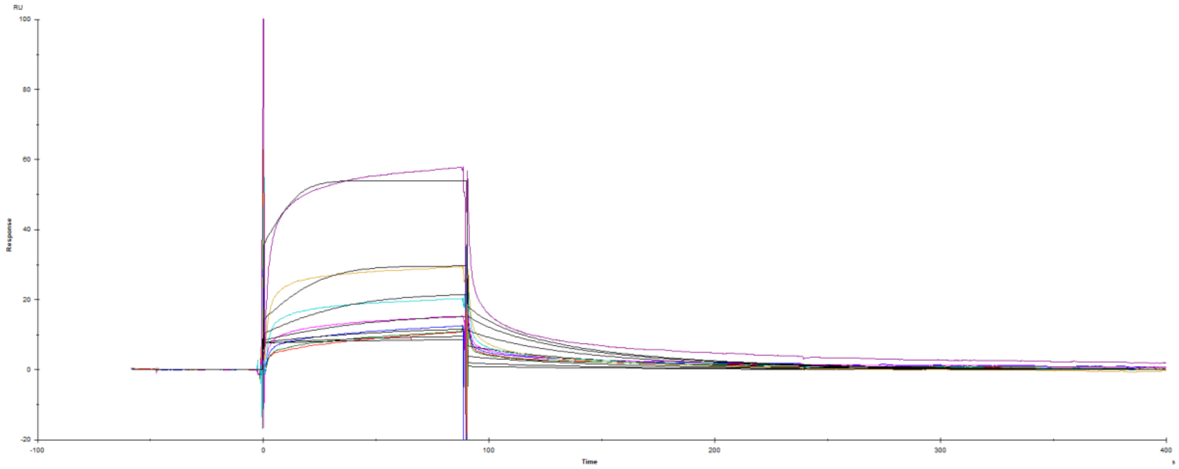

Figure S49. Sensorgram of LK4 on BAG3 full

SENSORGRAMS – BAG3 BD

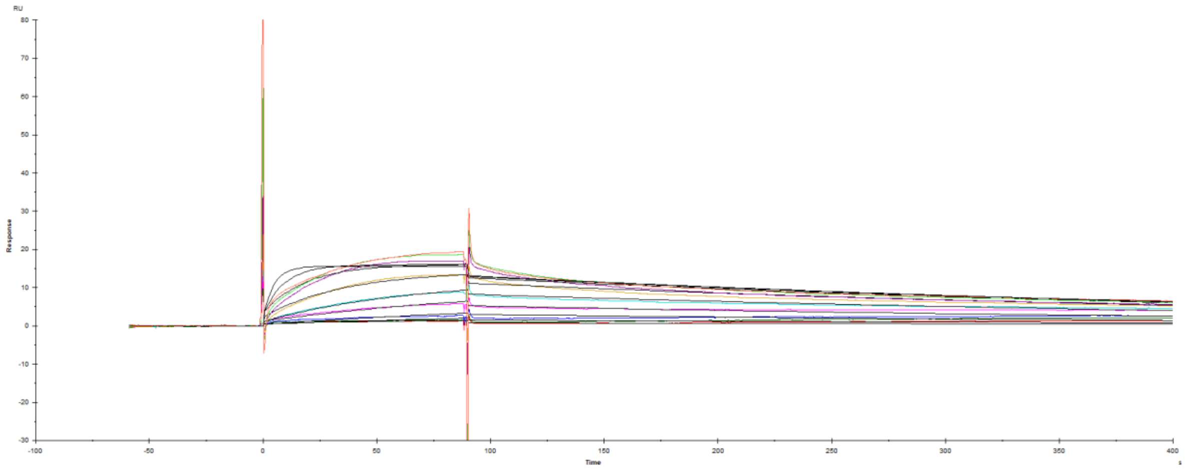

Figure S50. Sensorgram of compound 1 on BAG3-BD

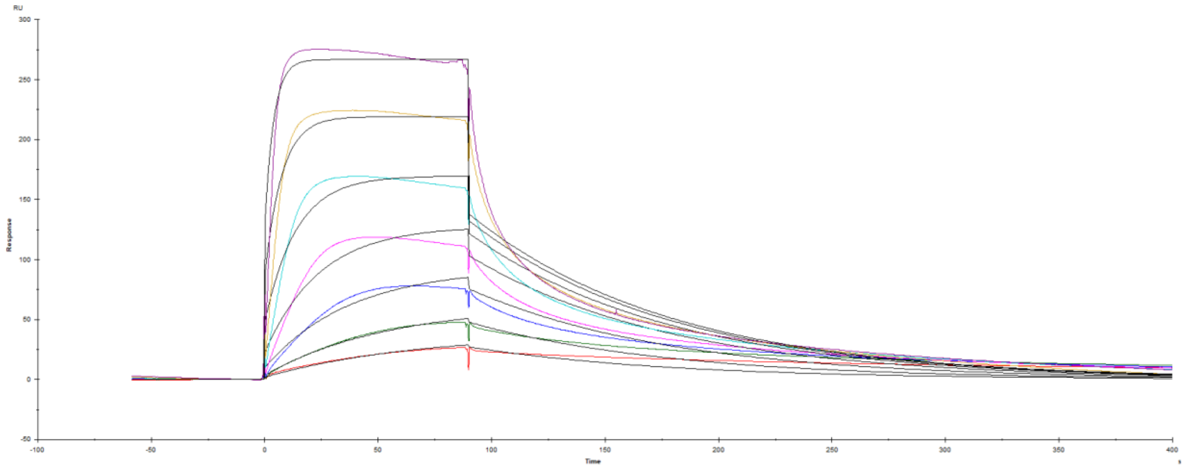

Figure S51. Sensorgram of compound 2 on BAG3-BD

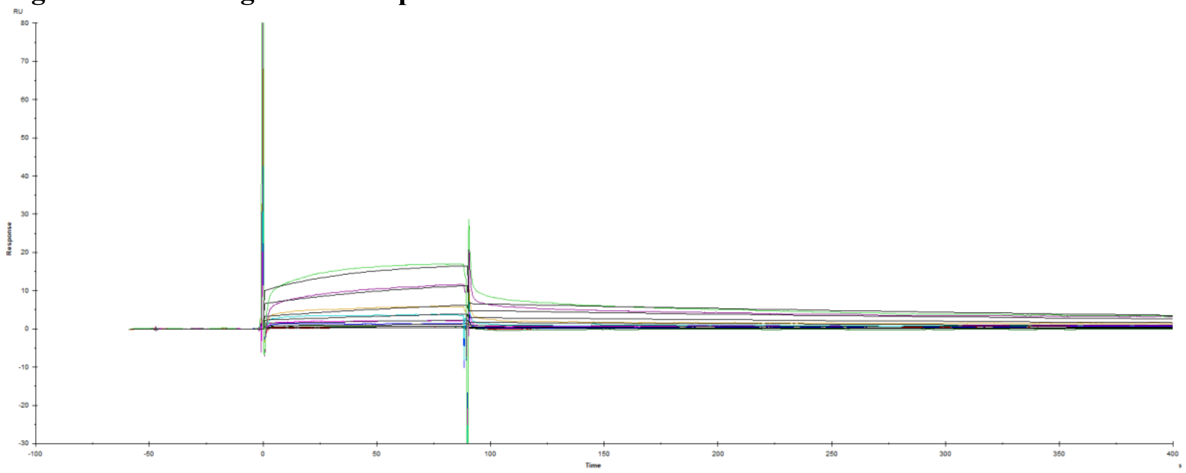

Figure S52. Sensorgram of compound 5 on BAG3-BD

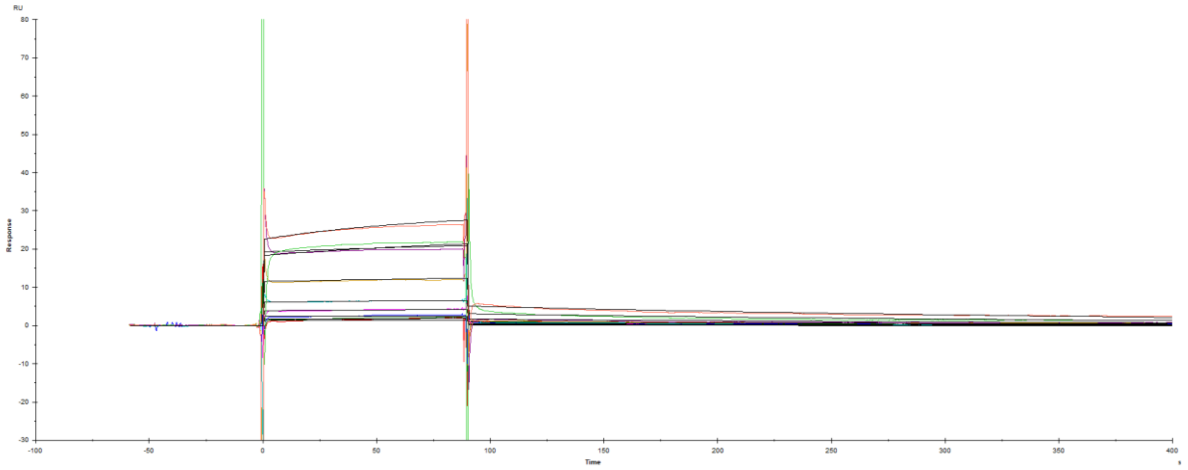

Figure S53. Sensorgram of compound 6 on BAG3-BD

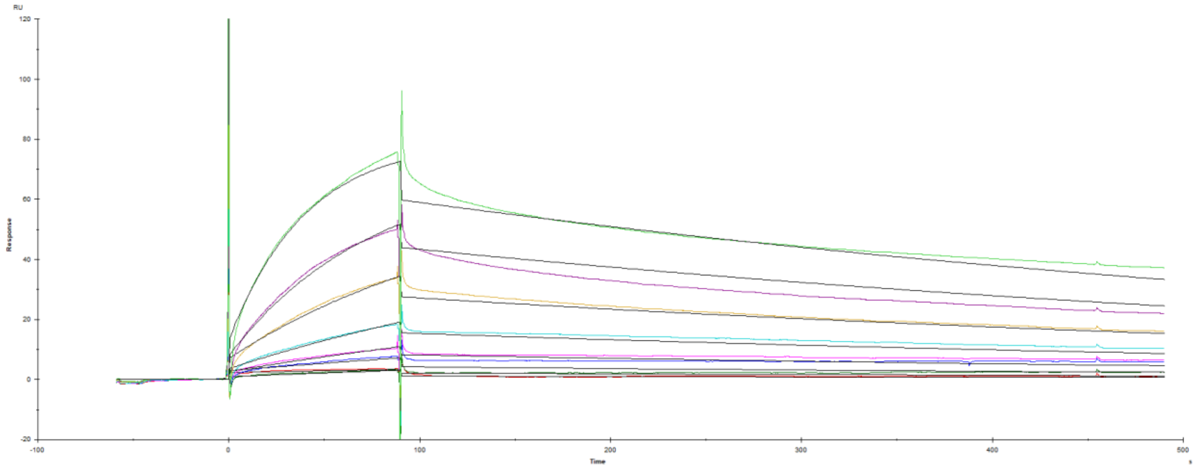

Figure S54. Sensorgram of compound 7 on BAG3-BD

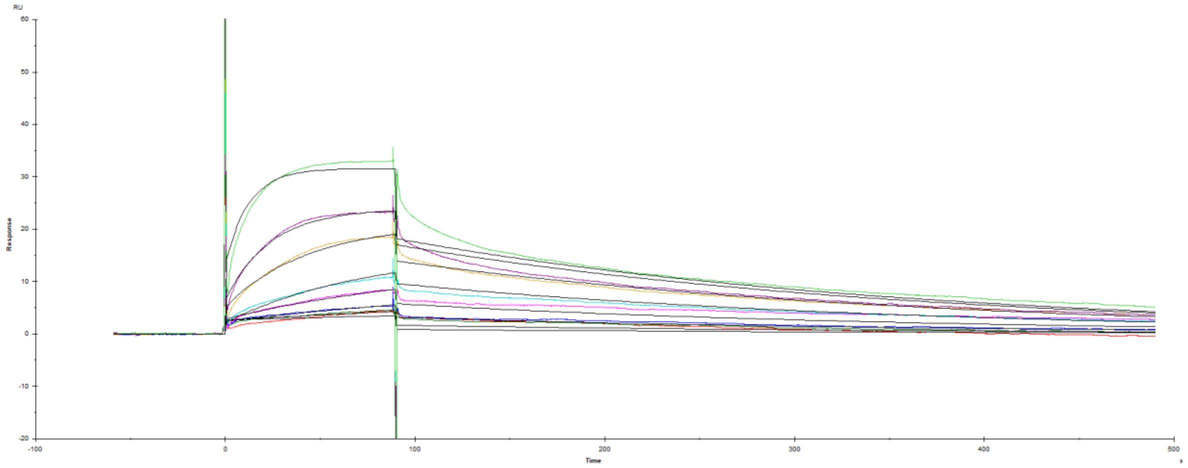

Figure S55. Sensorgram of compound 8 on BAG3-BD

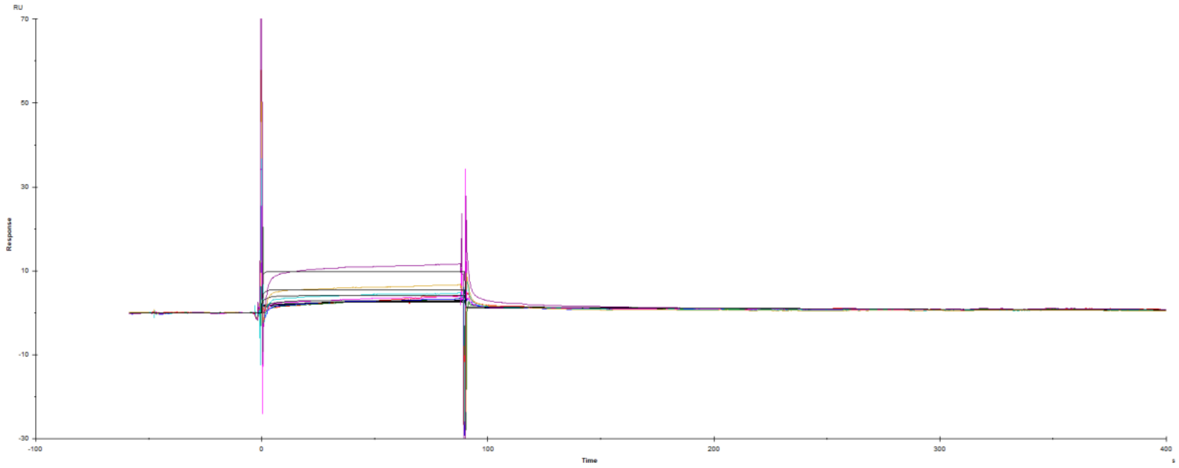

Figure S56. Sensorgram of LK4 on BAG3-BD

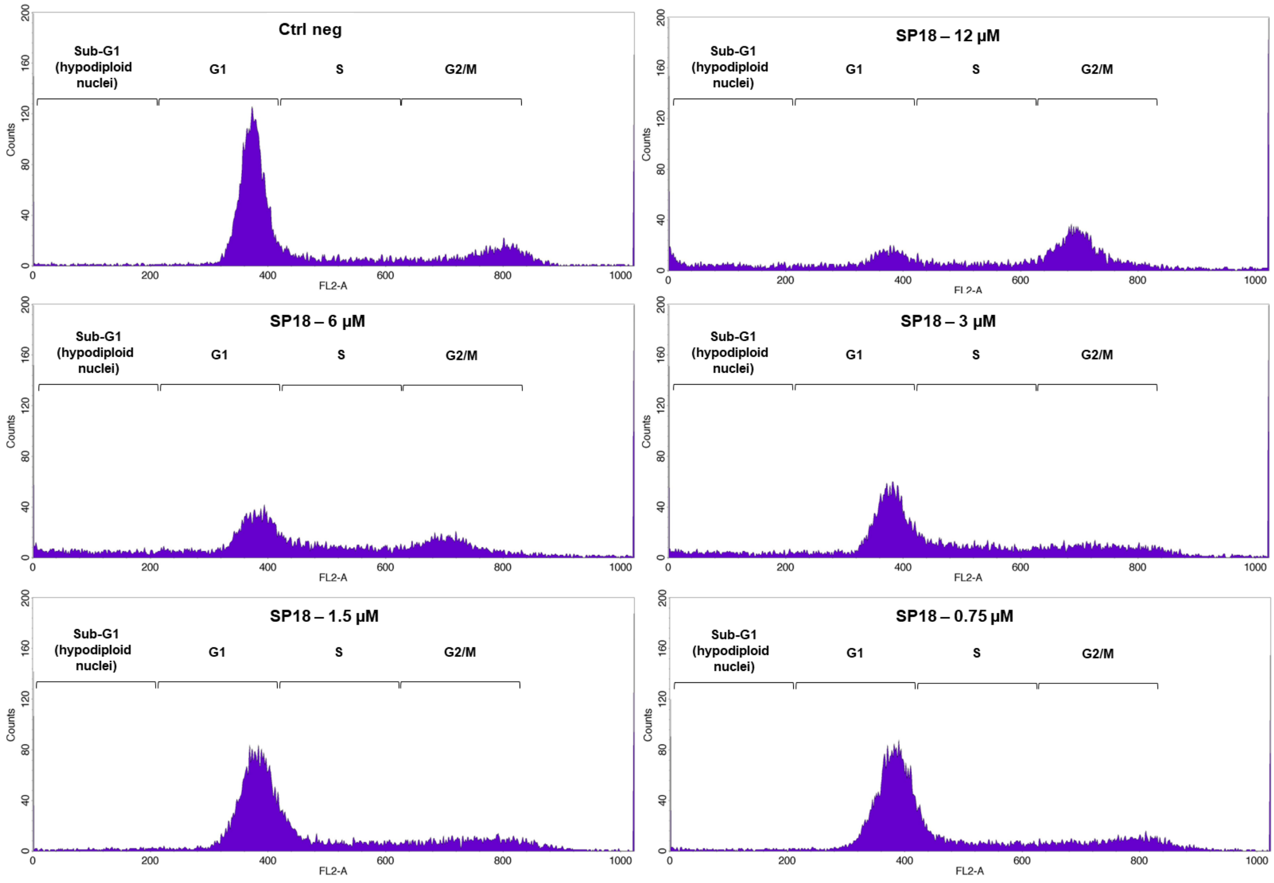

Figure S57: FACS histograms for cell cycle analysis of cells treated with SP18 at various concentrations for 48 hours.

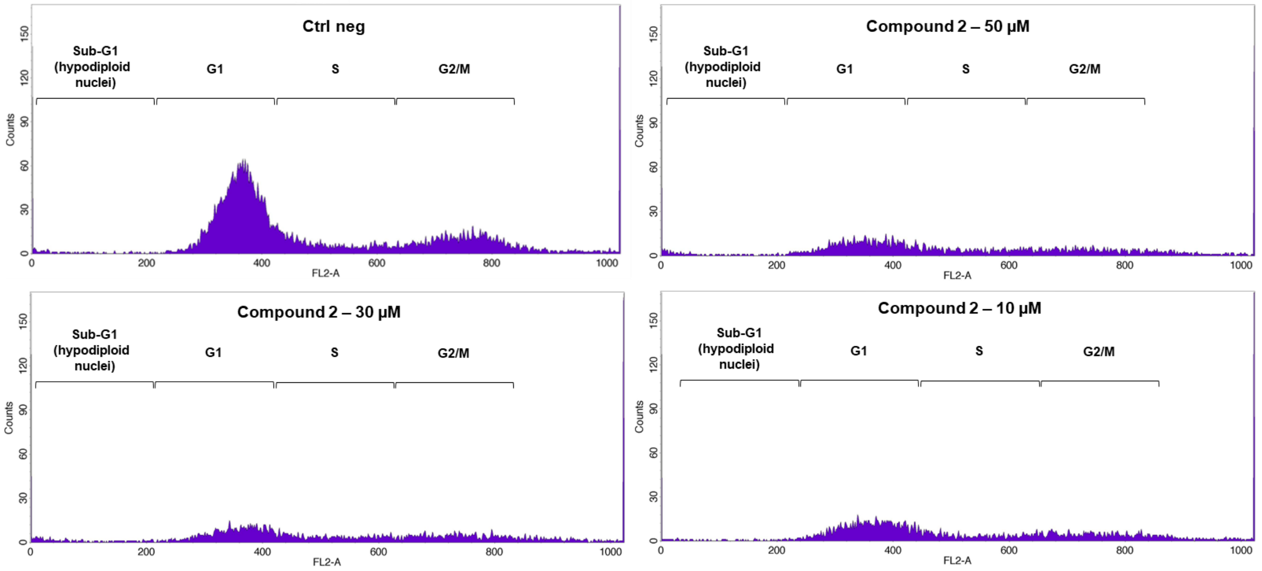

Figure S58: FACS histograms for cell cycle analysis of cells treated with compound 2 at various concentrations for 72 hours.
